# Comparative genomics of the tardigrades *Hypsibius dujardini* and *Ramazzottius varieornatus*

**DOI:** 10.1371/journal.pbio.2002266

**Published:** 2017-07-27

**Authors:** Yuki Yoshida, Georgios Koutsovoulos, Dominik R. Laetsch, Lewis Stevens, Sujai Kumar, Daiki D. Horikawa, Kyoko Ishino, Shiori Komine, Takekazu Kunieda, Masaru Tomita, Mark Blaxter, Kazuharu Arakawa

**Affiliations:** 1 Institute for Advanced Biosciences, Keio University, Tsuruoka, Yamagata, Japan; 2 Systems Biology Program, Graduate School of Media and Governance, Keio University, Kanagawa, Japan; 3 Institute of Evolutionary Biology, School of Biological Sciences, University of Edinburgh, Edinburgh, United Kingdom; 4 The James Hutton Institute, Dundee, United Kingdom; 5 Department of Biological Sciences, Graduate School of Science, University of Tokyo, Bunkyo-ku, Tokyo, Japan; The Wellcome Trust Sanger Institute, United Kingdom of Great Britain and Northern Ireland

## Abstract

Tardigrada, a phylum of meiofaunal organisms, have been at the center of discussions of the evolution of Metazoa, the biology of survival in extreme environments, and the role of horizontal gene transfer in animal evolution. Tardigrada are placed as sisters to Arthropoda and Onychophora (velvet worms) in the superphylum Panarthropoda by morphological analyses, but many molecular phylogenies fail to recover this relationship. This tension between molecular and morphological understanding may be very revealing of the mode and patterns of evolution of major groups. Limnoterrestrial tardigrades display extreme cryptobiotic abilities, including anhydrobiosis and cryobiosis, as do bdelloid rotifers, nematodes, and other animals of the water film. These extremophile behaviors challenge understanding of normal, aqueous physiology: how does a multicellular organism avoid lethal cellular collapse in the absence of liquid water? Meiofaunal species have been reported to have elevated levels of horizontal gene transfer (HGT) events, but how important this is in evolution, and particularly in the evolution of extremophile physiology, is unclear. To address these questions, we resequenced and reassembled the genome of *H*. *dujardini*, a limnoterrestrial tardigrade that can undergo anhydrobiosis only after extensive pre-exposure to drying conditions, and compared it to the genome of *R*. *varieornatus*, a related species with tolerance to rapid desiccation. The 2 species had contrasting gene expression responses to anhydrobiosis, with major transcriptional change in *H*. *dujardini* but limited regulation in *R*. *varieornatus*. We identified few horizontally transferred genes, but some of these were shown to be involved in entry into anhydrobiosis. Whole-genome molecular phylogenies supported a Tardigrada+Nematoda relationship over Tardigrada+Arthropoda, but rare genomic changes tended to support Tardigrada+Arthropoda.

## Author summary

Tardigrades are justly famous for their abilities to withstand environmental extremes. Many freshwater and terrestrial species can undergo anhydrobiosis—life without water—and thereby withstand desiccation, freezing, and other insults. We explored the comparative biology of anhydrobiosis in 2 species of tardigrade that differ in the mechanisms they use to enter anhydrobiosis. Using newly assembled and improved genomes, we find that *Ramazzottius varieornatus*, a species that can withstand rapid desiccation, differs from *Hypsibius dujardini*, a species that requires extended preconditioning, in not showing a major transcriptional response to anhydrobiosis induction. We identified a number of genetic systems in the tardigrades that likely play conserved, central roles in anhydrobiosis as well as species-unique components. Compared to previous estimates, our improved genomes show much reduced levels of horizontal gene transfer into tardigrade genomes, but some of the identified horizontal gene transfer (HGT) genes appear to be involved in anhydrobiosis. Using the improved genomes, we explored the evolutionary relationships of tardigrades and other molting animals, particularly nematodes and arthropods. We identified conflicting signals between sequence-based analyses, which found a relationship between tardigrades and nematodes, and analyses based on rare genomic changes, which tended to support the traditional tardigrade-arthropod link.

## Introduction

The superphylum Ecdysozoa emerged in the Precambrian, and ecdysozoans not only dominated the early Cambrian explosion but also are dominant (in terms of species, individuals, and biomass) today. The relationships of the 8 phyla within Ecdysozoa remain contentious, with morphological assessments, developmental analyses, and molecular phylogenetics yielding conflicting signals [1–3]. It has generally been accepted that Arthropoda, Onychophora (velvet worms), and Tardigrada (water bears or moss piglets) form a monophylum, Panarthropoda [2], and that Nematoda (roundworms) are closely allied to Nematomorpha (horsehair worms) and distinct from Panarthropoda. However, molecular phylogenies have frequently placed representatives of Tardigrada as sisters to Nematoda [1,3], invalidating Panarthropoda and challenging models of the evolution of complex morphological traits such as segmentation, serially repeated lateral appendages, the triradiate pharynx, and a tripartite central nervous system [4,5].

The key taxon in these disagreements is phylum Tardigrada. Nearly 1,200 species of tardigrades have been described [6]. All are members of the meiofauna—small animals that live in the water film and in interstices between sediment grains [6]. There are marine, freshwater, and terrestrial species. Many species of terrestrial tardigrades are cryptobiotic: they have the ability to survive extreme environmental challenges by entering a dormant state [7]. Common to these resistances is an ability to lose or exclude the bulk of body water, and anhydrobiotic tardigrades have been shown to have tolerance to high and low temperatures (including freezing), organic solvents, X- and gamma-rays, high pressure, and the vacuum of space [8–15]. The physiology of anhydrobiosis in tardigrades has been explored extensively, but little is currently known about its molecular bases [16,17]. Many other animals have cryptobiotic abilities, including some nematodes and arthropods [18], and comparison of the mechanisms in different independent acquisitions of this trait will reveal underlying common mechanisms.

Central to the development of tractable experimental models for cryptobiosis is the generation of high-quality genomic resources. Genome assemblies of 2 tardigrades, *H*. *dujardini* [19–21] and *R*. *varieornatus* [22], both in the family Hypsibiidae, have been published. *H*. *dujardini* is a limnoterrestrial tardigrade that is easy to culture [23], while *R*. *varieornatus* is a terrestrial tardigrade and highly tolerant of environmental extremes [24]. An experimental toolkit for *H*. *dujardini*, including RNA interference (RNAi) and in situ hybridization, is being developed [25]. *H*. *dujardini* is poorly cryptobiotic compared to *R*. *varieornatus*. *H*. *dujardini* requires 48 h of preconditioning at 85% relative humidity (RH) and a further 24 h in 30% RH [23] to enter cryptobiosis with high survival, while *R*. *varieornatus* can form a tun (the cryptobiotic form) within 30 min at 30% RH [26].

Several anhydrobiosis-related genes have been identified in Tardigrada. Catalases, superoxide dismutases (SODs), and glutathione reductases may protect against oxidative stress [27], and chaperones, such as heat shock protein 70 (HSP70) [28–30], may act to protect proteins from the denaturing effects of water loss [16,31,32]. Additionally, several tardigrade-specific gene families have been implicated in anhydrobiosis, based on their expression patterns. Cytosolic abundant heat soluble (CAHS), secretory abundant heat soluble (SAHS), late embryogenesis abundant protein mitochondrial (RvLEAM), mitochondrial abundant heat soluble protein (MAHS), and damage suppressor (Dsup) gene families have been implicated in *R*. *varieornatus* extremotolerance [22,33,34]. These gene families were named by their subcellular location or function, and expression of MAHS and Dsup in human tissue culture cell lines resulted in elevated levels of tolerance against osmotic stress and X-ray irradiation (approximately 4 Gy). Surprisingly, analyses of the *R*. *varieornatus* genome showed extensive gene loss in the peroxisome pathway and in stress signaling pathways, suggesting that this species is compromised in terms of reactive oxygen resistance and repair of cellular damage [22]. While loss of these pathways would be lethal for a normal organism, loss of these resistance pathways may be associated with anhydrobiosis.

Desiccation in some taxa induces the production of anhydroprotectants, small molecules that likely replace cellular water to stabilize cellular machinery. Trehalose, a disaccharide shown to contribute to anhydrobiosis in midges [35,36], nematodes [37], and artemia [38], is not present in the tardigrade *Milnesium tardigradum* [31]. Coupled with the ability of *R*. *varieornatus* to enter anhydrobiosis rapidly (i.e., without the need for extensive preparatory biosynthesis), this suggests that tardigrade anhydrobiosis does not rely on induced synthesis of protectants. Entry into anhydrobiosis in *H*. *dujardini* does require active transcription during preconditioning, suggesting the activation of a genetic program to regulate physiology. Inhibition of PP1/2A, an positive regulator of the FOXO transcription factor that induces antioxidative stress pathways, led to high lethality in *H*. *dujardini* during anhydrobiosis induction [23]. As *R*. *varieornatus* does not require preconditioning, systems critical to anhydrobiosis in *R*. *varieornatus* are likely to be constitutively expressed.

*H*. *dujardini* and *R*. *varieornatus* are relatively closely related (both are members of Hypsibiidae), and both have available genome sequences. The *R*. *varieornatus* genome has high contiguity and scores highly in all metrics of gene completeness [22]. For *H*. *dujardini*, 3 assemblies have been published. One has low contiguity (N50 length of 17 kb) and contains a high proportion of contaminating nontardigrade sequence, including approximately 40 Mb of bacterial sequence, and spans 212 Mb [19]. The other 2 assemblies, both at approximately 130 Mb [20,21], eliminated most contamination, but contained uncollapsed haploid segments because of unrecognized heterozygosity. The initial low-quality *H*. *dujardini* genome was published alongside a claim of extensive horizontal gene transfer (HGT) from bacteria and other taxa into the tardigrade genome and a suggestion that HGT might have contributed to the evolution of cryptobiosis [19]. The extensive HGT claim has been robustly challenged [20,21,39–41], but the debate as to the contribution of HGT to cryptobiosis remains open. The genomes of these species could be exploited for understanding the mechanisms of rapid-desiccation versus slow-desiccation strategies in tardigrades, the importance of HGT, and the resolution of the deep structure of the Ecdysozoa. However, the available genomes are not of equivalent quality.

We have generated a high-quality genome assembly for *H*. *dujardini*, from an array of data including single-tardigrade sequencing [42] and long, single-molecule reads, and using a heterozygosity-aware assembly method [43,44]. Gene finding and annotation with extensive RNA sequencing (RNA-Seq) data allowed us to predict a robust gene set. While most (60%) of the genes of *H*. *dujardini* had direct orthologues in an improved gene prediction for *R*. *varieornatus*, levels of synteny were very low. We identified an unremarkable proportion of potential HGTs. *H*. *dujardini* showed losses of peroxisome and stress signaling pathways, as described in *R*. *varieornatus*, as well as additional unique losses. Transcriptomic analysis of anhydrobiosis entry detected higher levels of regulation in *H*. *dujardini* compared to *R*. *varieornatus*, as predicted, including regulation of genes with antistress and apoptosis functions. Using single-copy orthologues, we reanalyzed the position of Tardigrada within Ecdysozoa and found strong support for a Tardigrade+Nematode clade, even when data from transcriptomes of a nematomorph, onychophorans, and other ecdysozoan phyla were included. However, rare genomic changes tended to support the traditional Panarthropoda. We discuss our findings in the context of how best to improve genomics of neglected species, the biology of anhydrobiosis, and conflicting models of ecdysozoan relationships.

## Results

### The genome of *H*. *dujardini*

The genome size of *H*. *dujardini* has been independently estimated by densitometry to be approximately 100 Mb [20,45], but the spans of existing assemblies exceed this, because of contamination with bacterial reads and uncollapsed heterozygosity of approximately 30%–60% of the span estimated from k-mer distributions. We generated new sequencing data (S1 Table) for *H*. *dujardini*. Tardigrades, originally purchased in mixed cultures from Sciento, were cultured with a single algal food source. Illumina short reads were generated from a single, cleaned tardigrade [42], and Pacific Biosciences (PacBio) long single-molecule reads from DNA from a bulk, cleaned tardigrade population (approximately 900,000 animals). We employed an assembly strategy that eliminated evident bacterial contamination [46] and eliminated residual heterozygosity. Our initial Platanus [44] genome assembly had a span of 99.3 Mb in 1,533 contigs, with an N50 length of 250 kb. Further scaffolding and gap filling [47] with PacBio reads and a Falcon [43] assembly of the PacBio reads produced a 104 Mb assembly in only 1,421 scaffolds and an N50 length of 342 kb and N90 count of 343 (Table 1). In comparison with previous assemblies, this assembly has improved contiguity and improved coverage of complete core eukaryotic genes [48,49]. Read coverage was relatively uniform throughout the genome (S1 Fig, S2 Table), with only a few short regions, likely repeats, with high coverage. We identified 29.6 Mb (28.5%) of the *H*. *dujardini* genome as being repetitive (S3 Table). Simple repeats covered 5.2% of the genome, with a longest repeat unit of 8,943 bp. Seven of the 8 longest repeats were of the same repeat unit (GATGGGTTTT)_n_, were found exclusively at 9 scaffold ends, and may correspond to telomeric sequence (S4 Table). The other long repeat was a simple repeat of (CAGA)_n_ and its complementary sequence (GTCT)_n_, and spanned 3.2 Mb (3% of the genome, longest unit 5,208 bp). We identified eighty-one 5.8S rRNA, two 18S rRNA, and three 28S rRNA loci with RNAmmer [50]. Scaffold0021 contains both 18S and 28S loci, and it is likely that multiple copies of the ribosomal RNA repeat locus have been collapsed in this scaffold, as it has very high read coverage (approximately 5,400-fold, compared to approximately 113-fold overall, suggesting approximately 48 copies). tRNAs for each amino acid were found (S2 Fig) [51]. Analysis of microRNA sequencing (miRNA-Seq) data with miRDeep [52] predicted 507 mature miRNA loci (S1 Data), of which 185 showed similarity with sequences in miRbase [53].

**Table 1:** Metrics of *H*. *dujardini* genome assemblies.

Data source	This work	Edinburgh [20]	North Carolina [19]
Sequencing technologies	Illumina and PacBio	Illumina	Illumina and PacBio
Genome version	nHd.3.0	nHd.2.3	tg
Scaffold number	1,421	13,202	16,175
Total scaffold length (bp)	104,155,103	134,961,902	212,302,995
Average scaffold length (bp)	73,297	10,222	13,125
Longest scaffold length (bp)	2,115,976	594,143	1,208,507*
Shortest scaffold length (bp)	1,000	500	2,002
N50 (bp) (number of scaffolds in N50)	342,180 (#85)	50,531 (#701)	17,496 (#3,422)
N90 (bp) (number of scaffolds in N90)	65,573 (#343)	6,194 (#3,280)	6,637 (#11,175)
CEGMA genes found (partial)	237 (240)	220 (241)	221 (235)
CEGMA gene duplication ratio	1.17 (1.23)	1.35 (1.56)	3.26 (3.53)
Complete BUSCO genes (%)	93.0	92.4	88.8

* The longest scaffolds in the North Carolina assembly are derived from bacterial contaminants.

BUSCO, Benchmarking Universal Single-Copy Orthologs; CEGMA, Core Eukaryotic Genes Mapping Approach; PacBio, Pacific Biosciences.

We generated RNA-Seq data from active and anhydrobiotic (“tun” stage) tardigrades and developmental stages of *H*. *dujardini* (S1 Table). Gene finding using BRAKER [54] predicted 19,901 genes, with 914 isoforms (version nHd3.0). This set of gene models had higher completeness and lower duplication scores compared to those predicted with MAKER [55], which uses RNA-Seq and protein evidence (Predicted proteome based BRAKER: 90.7% MAKER: 77.9%, genome based BRAKER: 86.3%, Metazoan lineage used). Minor manual editing of this gene set to break approximately 40 fused genes generated version nHd3.1. These coding sequence predictions lacked 5′ and 3′ untranslated regions. Mapping of RNA-Seq data to the predicted coding transcriptome showed an average mapping proportion of approximately 50%–70%, but the mapping proportion was over 95% against the genome (S5 Table). A similar mapping pattern for RNA-Seq data to predicted transcriptome was also observed for *R*. *varieornatus*. Over 70% of the *H*. *dujardini* transcripts assembled with Trinity [56] mapped to the predicted transcriptome, and a larger proportion to the genome (S6 Table). RNA-Seq reads that are not represented in the predicted coding transcriptome likely derived from UTR regions, unspliced introns, or promiscuous transcription. We inferred functional and similarity annotations for approximately 50% of the predicted proteome (Table 2).

**Table 2:** Comparison of the genomes of *H*. *dujardini* and *R*. *varieornatus*.

Assembly	*H*. *dujardini* 3.0		*R*. *varieornatus* 1.1		Difference	
**Genome**	Mb	%	bp	%	Mb	%
**Total span**	104.16	–	55.83	–	48.33	
**Genic**	59.03	56.67%	31.94	57.21%	27.09	56.06%
Exon span	25.25	24.24%	19.56	35.03%	5.69	11.78%
Intron span	33.78	32.43%	12.38	22.17%	21.40	44.28%
**Intergenic**	45.13	43.33%	23.89	42.79%	21.23	43.94%
Repeat	27.11	26.03%	10.11	18.12%	17.00	35.17%
**GENES**	# families	# genes	# families	# genes	#families	#genes
Number of genes	11,705	19,901	9,029	13,917	2,676	5,984
Number of proteins (including isoforms)		20,815		14,538		6,277
Species-specific singletons	4,364	4,364	1,995	1,995	2,369	2,369
Species-specific gene families	45	258	20	123	25	135
Shared gene families	7,296	15,279	7,014	11,799	258	3,480
Uniquely retained ancestral genes*	471	999	189	311	282	688
Genes with BLAST matches to SwissProt		8,337		6,978		
Genes with BLAST matches to TrEMBL		10,202		8,359		
Genes with InterPro domain matches		11,227		8,633		
Genes with Gene Ontology terms		7,804		6,030		
Eukaryote BUSCO completeness (%)		98.1		97.0		
All genes	Mean	Median	Mean	Median	Ratio of means	Ratio of medians
Gene length (bp)	2,966	2,131	2,295	1,641	1.29	1.30
Exon span (bp)	1,269	978	1,405	1074	0.90	0.91
Exon count (#)	5.94	4	6.02	4	0.99	1.00
Intron span (bp)	1,697	1,109	889	520	1.91	2.13
Intron count (#)	4.94	3	5.02	3	0.98	1.00
Single-copy orthologues**	Mean	Median	Mean	Median	Ratio of means	Ratio of medians
Gene length (bp)	3,716	2,776	2,579	1,929	1.44	1.44
Exon span (bp)	1,615	1,278	1,581	1,253	1.02	1.02
Exon count (#)	7.64	6	6.96	6	1.10	1.00
Intron span (bp)	2,101	1,475	998	635	2.11	2.32
Intron count (#)	3,716	2,776	2,579	1,929	1.44	1.44

* Uniquely retained ancestral genes include genes shared by only 1 tardigrade and at least 1 nontardigrade taxon.

** Single-copy orthologues: orthologues with coding sequence (CDS) lengths differing by more than 20% were not considered.

BLAST, Basic Local Alignment Search Tool; BUSCO, Benchmarking Universal Single-Copy Orthologs.

The *H*. *dujardini* nHd.3.0 genome assembly is available on a dedicated ENSEMBL [57] server, http://ensembl.tardigrades.org, where it can be compared with previous assemblies of *H*. *dujardini* and with the *R*. *varieornatus* assembly. The ENSEMBL database interface includes an application-programming interface (API) for scripted querying [58]. All data files (including supplementary data files and other analyses) are available from http://download.tardigrades.org, and a dedicated Basic Local Alignment Search Tool (BLAST) server is available at http://blast.tardigrades.org. All raw data files have been deposited in International Nucleotide Sequence Database Collaboration (INSDC) databases (National Center for Biotechnology Information [NCBI] and Sequence Read Archive [SRA], S1 Table), and the assembly (nHd3.1) has been submitted to NCBI under the accession ID MTYJ00000000.

### Comparisons with *R*. *varieornatus*

We compared this high-quality assembly of *H*. *dujardini* to that of *R*. *varieornatus* [22]. In initial comparisons, we noted that *R*. *varieornatus* had many single-exon loci that had no *H*. *dujardini* (or other) homologues. Reasoning that this might be a technical artifact, we updated gene models for *R*. *varieornatus* using BRAKER [54] with additional comprehensive RNA-Seq of developmental stages (S1 Table). The new prediction included 13,917 protein-coding genes (612 isoforms). This lower gene count compared to the original (19,521 genes) was largely due to a reduction in single-exon genes with no transcript support (from 5,626 in version 1 to 1,777 in the current annotation). Most (12,752, 90%) of the BRAKER-predicted genes were also found in the original set. In both species, some predicted genes may derive from transposons, as 2,474 *H*. *dujardini* and 626 *R*. *varieornatus* proteins matched Dfam domains [59]. While several of these putatively transposon-derived predictions have a Swiss-Prot [60] homologue (*H*. *dujardini*: 915, 37%; *R*. *varieornatus*: 274, 44%), most had very low expression levels.

**Fig 1:**
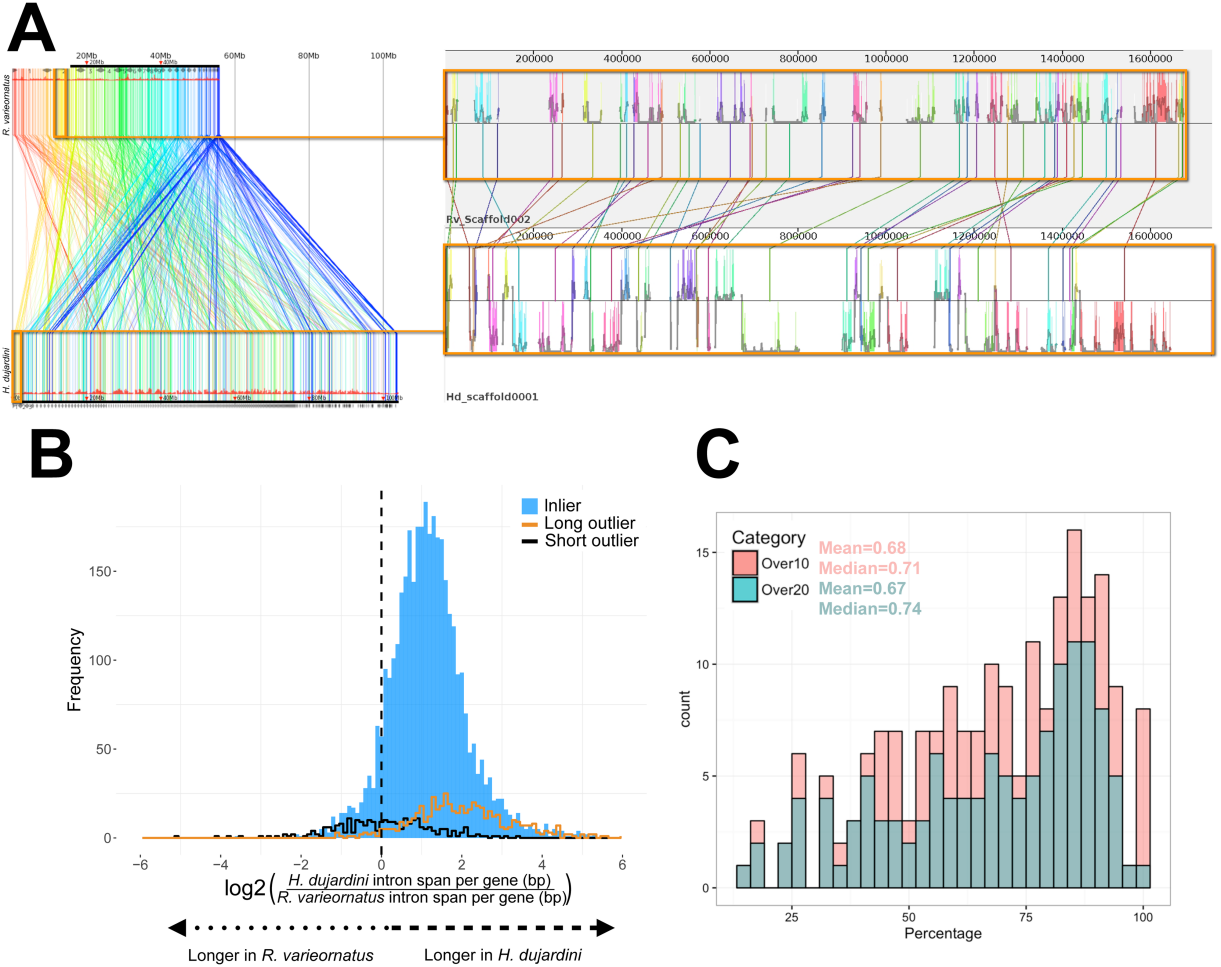
The genomes of *H*. *dujardini* and *R*. *varieornatus*. (A) Linkage conservation but limited synteny between *H*. *dujardini* and *R*. *varieornatus*. Whole genome alignment was performed with Murasaki [61]. The left panel shows the whole genome alignment. Similar regions are linked by a line colored following a spectrum based on the start position in *R*. *varieornatus*. To the right is a realignment of the initial segment of *H*. *dujardini* scaffold0001 (lower), showing matches to several portions of *R*. *varieornatus* Scaffold0002 (above), illustrating the several inversions that must have taken place. The histograms show pairwise nucleotide sequence identity between these 2 segments. (B) Increased intron span in *H*. *dujardini*. *H*. *dujardini* genes are longer because of expanded introns. Frequency histogram of log_2_ ratio of intron span per gene in 4,728 *H*. *dujardini* genes compared to their orthologues in *R*. *varieornatus*. Outliers are defined as genes in *H*. *dujardini* whose coding sequences (CDSs) are 20% longer (long outliers; orange; 576 genes) or 20% shorter (short outliers; black; 294 genes) than their orthologues in *R*. *varieornatus*. Data available at https://github.com/abs-yy/Hypsibius_dujardini_manuscript/blob/master/data/Fig1B_HDUJA_RVARI.gene_structure_matrix.ONE-TO-ONE.txt. (C) Gene neighborhood conservation between *H*. *dujardini* and *R*. *varieornatus*. To test conservation of gene neighborhoods, we asked whether genes found together in *H*. *dujardini* were also found close together in *R*. *varieornatus*. Taking the set of genes on each long *H*. *dujardini* scaffold, we identified the locations of the reciprocal best Basic Local Alignment Search Tool (BLAST) hit orthologues in *R*. *varieornatus* and counted the maximal proportion mapping to 1 *R*. *varieornatus* scaffold. *H*. *dujardini* scaffolds were binned and counted by this proportion. As short scaffolds, with fewer genes, might bias this analysis, we performed analyses independently on scaffolds with >10 genes and scaffolds with >20 genes. Data available at https://github.com/abs-yy/Hypsibius_dujardini_manuscript/blob/master/data/Fig1C_Gene-neighborhoods-conservation.txt.

One striking difference between the 2 species was in genome size, as represented by assembly span: the *R*. *varieornatus* assembly had a span of 55 Mb, half that of *H*. *dujardini* (Table 2). This difference could have arisen through whole genome duplication, segmental duplication, or more piecemeal processes of genome expansion or contraction. *H*. *dujardini* had 5,984 more predicted genes than *R*. *varieornatus*. These spanned approximately 23 Mb and accounted for about half of the additional span. There was no difference in number of exons per gene between orthologues or in the whole predicted gene set. However, comparing orthologues, the intron span per gene in *H*. *dujardini* was on average twice that in *R*. *varieornatus* (Fig 1B), and gene length (measured as start codon to stop codon in coding exons) was approximately 1.3-fold greater in *H*. *dujardini* (Table 2, S3 Fig). There was more intergenic noncoding DNA in *H*. *dujardini*, largely explained by an increase in the repeat content (28.6 Mb in *H*. *dujardini* versus 11.1 Mb in *R*. *varieornatus*).

Whole genome alignments of *R*. *varieornatus* and *H*. *dujardini* using Murasaki [61] revealed a low level of synteny but evidence for conserved linkage at the genome scale, with little conservation of gene order beyond a few loci. For example, comparison of *R*. *varieornatus* Scaffold002 of with *H*. *dujardini* scaffold0001 showed linkage, with many orthologous (genome-wide bidirectional best BLAST hit) loci across approximately 1.7 Mb of the *H*. *dujardini* genome (Fig 1A). A high proportion of orthologues of genes located on the same scaffold in *H*. *dujardini* were also in one scaffold in *R*. *varieornatus*, implying that intrachromosomal rearrangement may be the reason for the low level of synteny (Fig 1C).

We defined protein families in the *H*. *dujardini* and new *R*. *varieornatus* predicted proteomes, along with a selection of other ecdysozoan and other animal predicted proteomes (S7 Table), using OrthoFinder [62], including predicted proteomes from fully sequenced genomes or predicted proteomes from the fully sequenced genomes and (likely partial) transcriptomes in two independent analyses. Using these protein families, we identified orthologues for phylogenetic analysis and explored patterns of gene family expansion and contraction, using KinFin [63]. We identified 144,610 protein families in the analysis of 29 fully sequenced genome species. Of these families, 87.9% were species specific (with singletons accounting for 11.6% of amino acid span, and multi-protein clusters accounting for 1.2% of span). While only 12.1% of clusters contained members from ≥2 predicted proteomes, they accounted for the majority of the amino acid span (87.2%). *H*. *dujardini* had more species-specific genes than *R*. *varieornatus* and had more duplicate genes in gene families shared with *R*. *varieornatus* (Table 2). *H*. *dujardini* also had more genes shared with nontardigrade outgroups, suggesting loss in *R*. *varieornatus*. Many families had more members in tardigrades compared to other taxa, and 3 had fewer members (115 had uncorrected Mann-Whitney U-test probabilities <0.01, but none had differential presence after Bonferroni correction). In 9 of the families with tardigrade overrepresentation, tardigrades had more than four times as many members as the average of the other species (S2 Data).

There were 1,486 clusters composed solely of proteins predicted from the 2 tardigrade genomes. Of those, 365 (24.56%) had a congruent domain architecture in both species, including 53 peptidase clusters, 27 kinase clusters, and 29 clusters associated with signaling function, including 18 G-protein coupled receptors (see S3 Data). While these annotations are commonly found in clade-specific families, suggesting that innovation in these classes of function is a general feature in metazoan evolution, of particular interest was innovation in the Wnt signaling pathway. Tardigrade-unique clusters included Wnt, Frizzled, and chibby proteins. Of relevance to cryptobiosis, 21 clusters with domain annotation relevant to genome repair and maintenance were synapomorphic for Tardigrada, including molecular chaperones (2), histone/chromatin maintenance proteins (11), genome repair systems (4), nucleases (2), and chromosome cohesion components (2) (see below).

### HGT in tardigrade genomes

**Fig 2:**
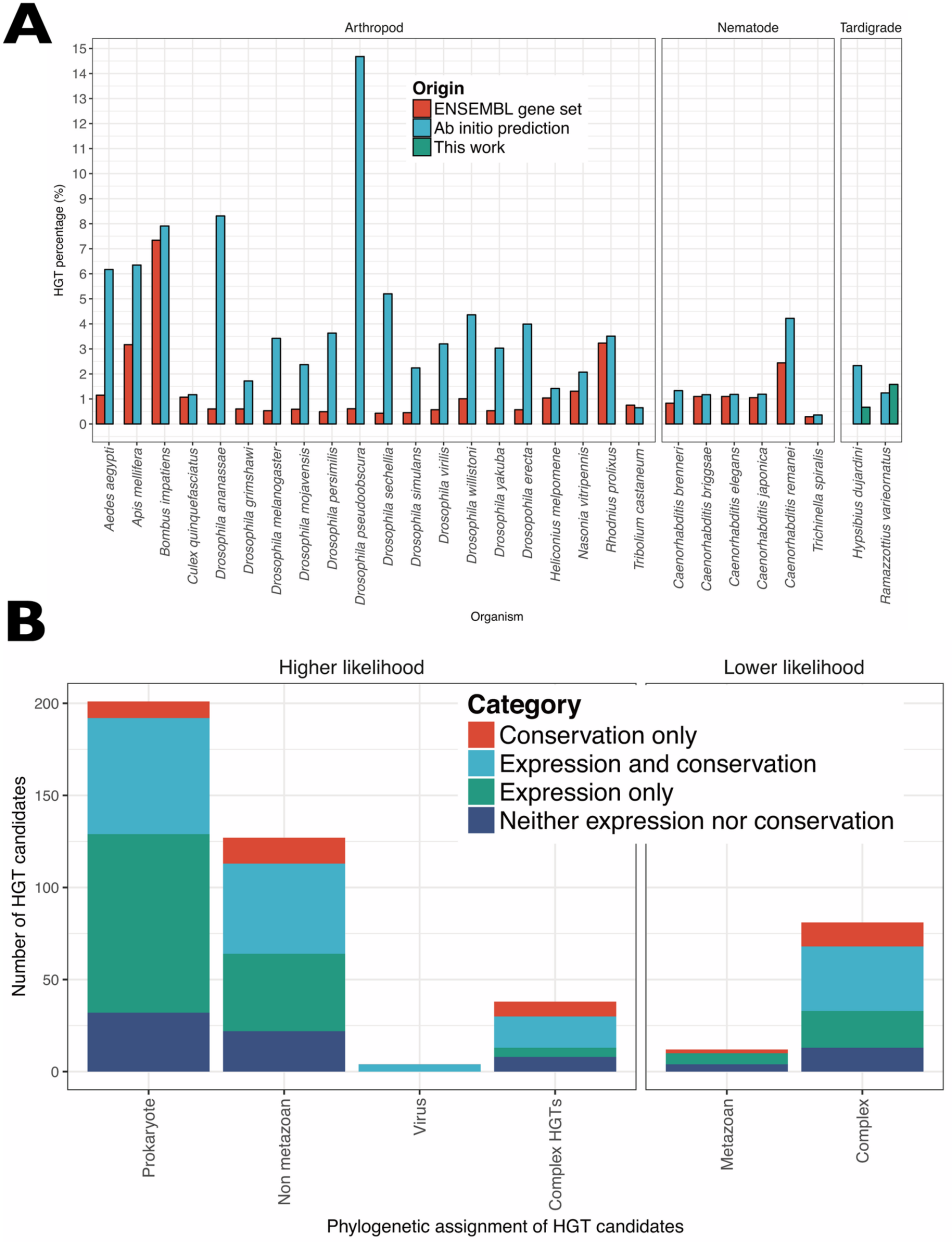
Horizontal gene transfer in *H*. *dujardini*. (A) Horizontal gene transfer ratios in various metazoa. For a set of assembled arthropod and nematode genomes, genes were repredicted ab initio with Augustus. Putative horizontal gene transfer (HGT) loci were identified using the HGT index for the longest transcript for each gene from the new and the ENSEMBL reference gene sets. In most species, the ab initio gene sets had elevated numbers of potential HGT loci compared to their ENSEMBL representations. Data available at https://github.com/abs-yy/Hypsibius_dujardini_manuscript/blob/master/data/Fig2A_HGT-content-in-metazoa.txt. (B) Classification of HGT candidates in *H*. *dujardini*. Classification of the initial HGT candidates identified in *H*. *dujardini* by their phylogenetic annotation (prokaryotic, nonmetazoan eukaryotic, viral, complex HGT, and likely non-HGT metazoan and complex), their support in RNA-Seq expression data, and the presence of a homologue in *R*. *varieornatus*. Data available at https://github.com/abs-yy/Hypsibius_dujardini_manuscript/blob/master/data/Fig2B_HGT_content_in_Hdujardini.csv.

HGT is an interesting but contested phenomenon in animals. Many newly sequenced genomes have been reported to have relatively high levels of HGT, and genomes subject to intense curation efforts tend to have lower HGT estimates. We performed ab initio gene finding on the genomes of the model species *Caenorhabditis elegans* and *Drosophila melanogaster* with Augustus [64] and used the HGT index approach [65], which simply classifies loci based on the ratio of their best BLAST scores to ingroup and potential donor taxon databases, to identify candidates. Compared with their mature annotations, we found elevated proportions of putative HGTs in both species (*C*. *elegans*: 2.09% of all genes; *D*. *melanogaster*: 4.67%). We observed similarly elevated rates of putative HGT loci, as assessed by the HGT index, in gene sets generated by ab initio gene finding in additional arthropod and nematode genomes compared to their mature annotation (Fig 2A, S8 Table). Thus, the numbers of HGT events found in the genomes of *H*. *dujardini* and *R*. *varieornatus* are likely to have been overestimated in these initial, uncurated gene predictions, even after sequence contamination has been removed, as seen in the *H*. *dujardini* assembly of Boothby et al. [41].

Using the HGT index approach, we identified 463 genes as potential HGT candidates in *H*. *dujardini* (S4 Data). Using Diamond BLASTX [66] instead of standard BLASTX [67,68] made only a minor difference in the number of potential HGT events predicted (446 genes). We sifted the initial 463 *H*. *dujardini* candidates through a series of biological filters. A true HGT locus will show affinity with its source taxon when analyzed phylogenetically (a more sensitive test than simple BLAST score ratio). Four-fifths of these loci (357) were confirmed as HGT events by Randomized Axelerated Maximum Likelihood (RAxML) [69] analysis of aligned sequences (Fig 2B). For 13 candidates, there were not enough homologues found in public databases to estimate phylogenies. HGT genes are expected to be incorporated into the host genome and to persist through evolutionary time. Only 164 of the RAxML-confirmed *H*. *dujardini* candidates had homologues in *R*. *varieornatus*, indicating phyletic perdurance (S2 Data). HGT loci will acquire gene structure and expression characteristics of their host metazoan genome. We identified expression at greater than 1 transcript per million (TPM) in any library for 338 HGT candidates. While metazoan genes usually contain spliceosomal introns, and 367 of the candidate HGT gene models included introns, we regard this a lower-quality validation criterion, as gene-finding algorithms are programmed to identify introns. Therefore, our highest-credibility current estimate for HGT into the genome of *H*. *dujardini* is 133 genes (0.7% of all genes), with a less-credible set, showing conservation, expression, and/or phylogenetic validation of 357 (1.8%) and an upper bound of 463 (2.3%). This is congruent with estimates of 1.6% HGT candidates (out of 13,917 genes) for *R*. *varieornatus* [22].

The putative HGT loci tended to be clustered in the tardigrade genomes, with many gene neighbors of HGT loci also predicted to be HGTs (S4 Fig). We found 58 clusters of HGT loci in *H*. *dujardini* and 14 in *R*. *varieornatus* (S6 Data). The largest clusters included up to 6 genes from the same gene family and may have arisen through tandem duplication. These tandem duplication clusters included intradiol ring-cleavage dioxygenases, uridine diphosphate (UDP) glycosyltransferases, and alpha/beta fold hydrolases. Several clusters of UDP glycosyltransferases with signatures of HGT from plants were identified in the *H*. *dujardini* genome, 1 of which included 6 UDP glycosyltransferases within 12 genes (loci between the genes bHd03905 and bHd03916). *H*. *dujardini* had 40 UDP glycosyltransferase genes, 29 of which were classified as glucuronosyltransferase (UGT, K00699) by Kyoto Encyclopedia of Genes and Genomes (KEGG) ORTHOLOG mapping with the KEGG Automatic Annotation Server (KAAS) [70], and of these 27 were HGT candidates. While UGT can function in a number of pathways, we found that the whole ascorbate synthesis pathway, in which UGT metabolizes UDP-D-glucuronate to D-Glucuronate, has been acquired by HGT in *H*. *dujardini*. *R*. *varieornatus* has only acquired L-gulonolactone oxidase (S5 Fig). Gluconolactonase and L-gluonolactone oxidase were consistently expressed at low levels (approximately 10–30 TPM), but L-ascorbate degradation enzyme L-ascorbate oxidase was not expressed (TPM < 1).

### The genomics of anhydrobiosis in tardigrades

We explored the *H*. *dujardini* proteome and the reannotated *R*. *varieornatus* proteome for loci implicated in anhydrobiosis. In the new *R*. *varieornatus* proteome, we found 16 CAHS loci and 13 SAHS loci and 1 copy each of MAHS, RvLEAM, and Dsup. In *H*. *dujardini*, we identified 12 CAHS loci, 10 SAHS loci, and single members of the RvLEAM and MAHS families (S9 Table). Direct interrogation of the *H*. *dujardini* genome with *R*. *varieornatus* loci identified an additional possible CAHS-like locus and an additional SAHS-like locus. We found no evidence for a *H*. *dujardini* homologue of Dsup. Phylogenetic analyses revealed a unique duplication of CAHS3 in *R*. *varieornatus*. No SAHS2 orthologue was found in *H*. *dujardini* (S6 Fig), and most of the *H*. *dujardini* SAHS loci belonged to a species-specific expansion that was orthologous to a single *R*. *varieornatus* SAHS locus, RvSAHS13. SAHS1-like genes in *H*. *dujardini* and SAHS1- and SAHS2-like genes in *R*. *varieornatus* were locally duplicated, forming SAHS clusters on single scaffolds.

**Fig 3:**
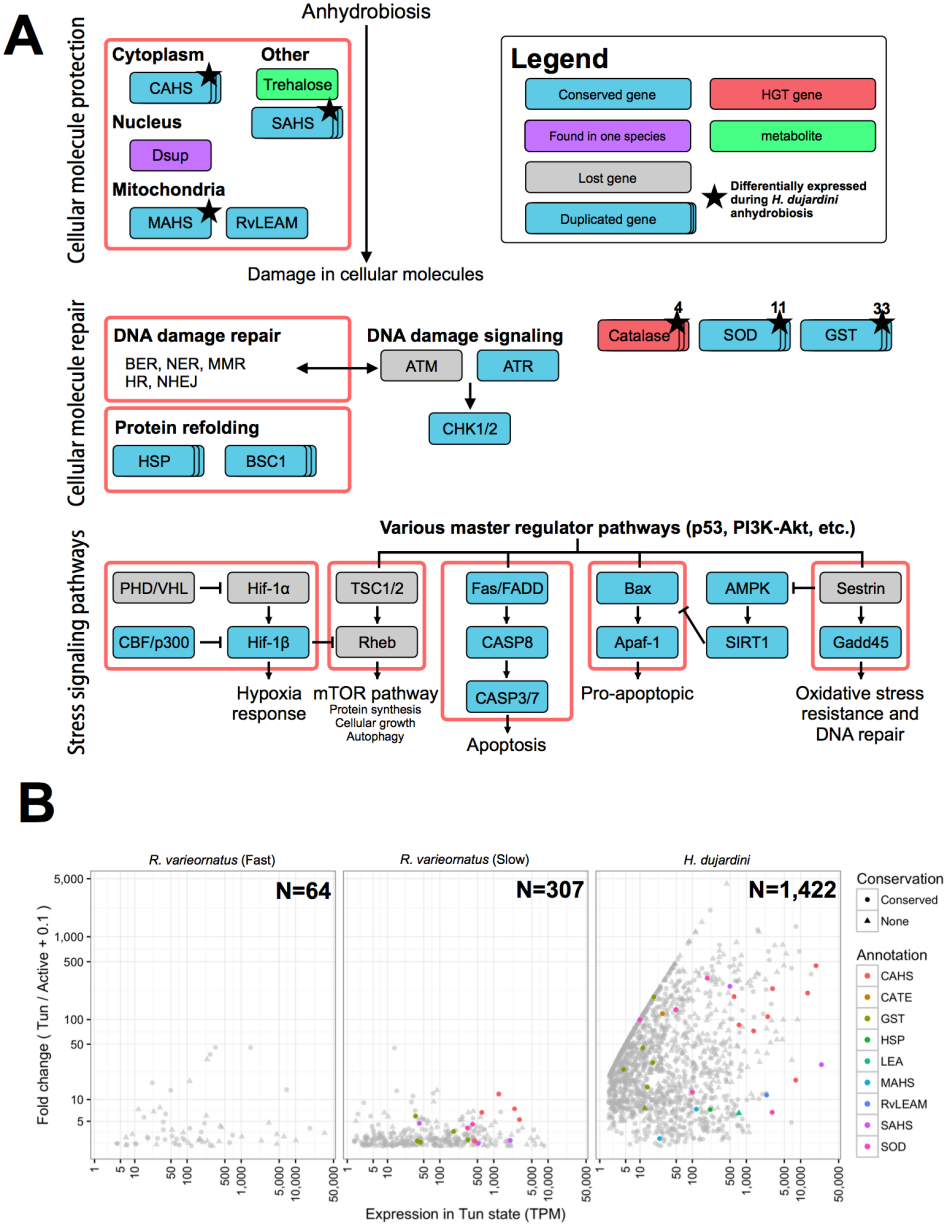
The genomics of anhydrobiosis in tardigrades. (A) Gene losses in Hypsibiidae. Gene losses were detected by mapping to Kyoto Encyclopedia of Genes and Genomes (KEGG) pathways using KEGG Automatic Annotation Server (KAAS) and validated by Basic Local Alignment Search Tool (BLAST) TBLASTN search of KEGG orthologue gene amino acid sequences. Light blue and gray boxes indicate genes conserved and lost in both tardigrades, respectively. Furthermore, purple boxes represent genes retained in only 1 species, and red boxes represent genes that have been detected as horizontal gene transfer (HGT). The numbers on the top right of the boxes indicate copy numbers of multiple copy genes in *H*. *dujardini*. Genes annotated as CASP3 and CDC25A have contradicting annotation with KAAS and Swiss-Prot; however, the KAAS annotation was used. (B) Differential gene expression in tardigrades on entry to the anhydrobiotic state. The transcript per million (TPM) expression for each sample was calculated using Kallisto, and the fold change between active and tun and the TPM expression in the tun state were plotted. Genes that likely contribute to anhydrobiosis were colored. Genes that had an orthologue in the other species are plotted as circles; other genes are plotted as triangles. Data available at https://github.com/abs-yy/Hypsibius_dujardini_manuscript/blob/master/data/Fig3B_gene-expression-DEGs-FCandTUN-annotated.txt. AMPK, 5' adenosine monophosphate-activated protein kinase; ATM, ataxia-telangiectasia mutated; ATR, ataxia telangiectasia and Rad3-related protein; BER, base excision repair; BSC1, bypass of stop codon protein 1; CAHS, cytosolic abundant heat soluble; CASP8, caspase 8; CATE, catalase; CBF, C-repeat-binding factor; CHK1/2, checkpoint kinase 1/2; Dsup, damage suppressor; GST, glutathione S-transferase; HR, homologous recombination; HSP, heat shock protein; MAHS, mitochondrial abundant heat soluble protein; MMR, mismatch repair; mTOR, mechanistic target of rapamycin; NER, nucleotide excision repair; NHEJ, nonhomologous end joining; RvLEAM, late embryogenesis abundant protein mitochondrial; PHD, plant homeodomain; SAHS, secretory abundant heat soluble; SIRT1, sirtuin 1; SOD, superoxide dismutase; TSC1/2, tuberous sclerosis 1/2; VHL, von Hippel-Lindau tumor suppressor.

*R*. *varieornatus* was reported to have undergone extensive gene loss in the stress-responsive transducer of mechanistic target of rapamycin (mTOR) pathway and in the peroxisome pathway, which generates H_2_O_2_ during the beta-oxidation of fatty lipids. *H*. *dujardini* was similarly compromised (Fig 3A). We identified additional gene losses in the peroxisome pathway in *H*. *dujardini*, as peroxisome proteins PEK5, PEK10, and PEK12, while present in *R*. *varieornatus*, were not found in *H*. *dujardini* (TBLASTN search against genome with an E-value threshold of 1E-3).

To identify gene functions associated with anhydrobiosis, we explored differential gene expression in fully hydrated and postdesiccation samples from both species. We compared single individual RNA-Seq of *H*. *dujardini* undergoing anhydrobiosis [42] with new data for *R*. *varieornatus* induced to enter anhydrobiosis in 2 ways: slow desiccation (approximately 24 h) and fast desiccation (approximately 30 min). Successful anhydrobiosis was assumed when >90% of the samples prepared in the same chamber recovered after rehydration. Many more genes were differentially up-regulated by entry into anhydrobiosis in *H*. *dujardini* (1,422 genes, 7.1%) than in *R*. *varieornatus* (fast desiccation: 64 genes, 0.5%; slow desiccation: 307 genes, 2.2%) (S6 Data). The fold change distribution of the whole transcriptome of *H*. *dujardini* (mean 8.33, median 0.91 ± 69.90 SD) was significantly broader than those of both fast (0.67, 0.48 ± 2.25) and slow (0.77, 0.65 ± 0.79) desiccation *R*. *varieornatus* (U-test, *p*-value < 0.001) (Fig 3B).

For the loci differentially expressed in anhydrobiosis (S7 Data), we investigated their membership of gene families with elevated numbers in tardigrades and functional annotations associated with anhydrobiosis. Proteins with functions related to protection from oxidants, such as SOD and peroxiredoxin, were found to have been extensively duplicated in tardigrades. In addition, the mitochondrial chaperone (BSC1), osmotic stress-related transcription factor NFAT5, and apoptosis related-gene poly(ADP-ribose) polymerase (PARP) families were expanded in tardigrades. Chaperones were extensively expanded in *H*. *dujardini* (HSP70, DnaK, and DnaJ subfamily C-5, C-13, and B-12), and the DnaJ subfamily B3, B-8 was expanded in *R*. *varieornatus*. In *H*. *dujardini*, we found 5 copies of DNA repair endonuclease XPF, which functions in the nucleotide-excision repair pathway, and, in *R*. *varieornatus*, 4 copies of the double-stranded break repair protein MRE11 (as reported previously [22]) and additional copies of DNA ligase 4, from the nonhomologous end-joining pathway. In both *R*. *varieornatus* [22] and *H*. *dujardini*, some of the genes with anhydrobiosis-related function appear to have been acquired through HGT. All copies of catalase were high-confidence HGTs (S5 Data), and 1 copy was differentially expressed during *H*. *dujardini* anhydrobiosis (expression rises from 0 TPM to 27.5 TPM during slow dehydration in *H*. *dujardini*). *R*. *varieornatus* had 11 trehalase loci (9 trehalases and 2 acid trehalase-like proteins). While *H*. *dujardini* did not have an orthologue of trehalose-6-phosphatase synthase (TPS), a gene required for trehalose synthesis, *R*. *varieornatus* had a HGT-derived TPS (S5 Fig). Previous studies in *M*. *tardigradum* have shown that trehalose does not accumulate during anhydrobiosis, and this is supported by the low expression of the *R*. *varieornatus* TPS gene (10–20 TPM in active and tun states). We note that the *R*. *varieornatus* TPS had the highest similarity to TPS from bacterial species in Bacteriodetes, including *Chitinophaga*, which was one of the contaminating organisms in the Boothby et al. assembly [40]. The *R*. *varieornatus* locus contains spliceosomal introns that do not compromise the TPS protein sequence and is surrounded by metazoan-affinity loci. The ascorbate synthesis pathway appears to have been acquired through HGT in *H*. *dujardini*, and a horizontally acquired L-gulonolactone oxidase was identified in *R*. *varieornatus* (S5 Fig).

Several protection-related genes were differentially expressed in anhydrobiotic *H*. *dujardini*, including CAHS (8 loci of 15), SAHS (2 of 10), RvLEAM (1 of 1), and MAHS (1 of 1). Loci involved in reactive oxygen protection (5 SOD genes, 6 glutathione-S transferase genes, a catalase gene, and a LEA gene) were up-regulated under desiccation. Interestingly, 2 trehalase loci were up-regulated, even though we were unable to identify any TPS loci in *H*. *dujardini*. We also identified differentially expressed transcription factors that may regulate anhydrobiotic responses. Two calcium-signaling factors, calmodulin (CaM) and a cyclic nucleotide-gated channel (CNG-3), were both up-regulated, which may drive cAMP synthesis through adenylate cyclase. Although *R*. *varieornatus* is capable of rapid anhydrobiosis induction, complete desiccation is unlikely to be as rapid in natural environments, and regulation of gene expression under slow desiccation might reflect a more likely scenario. Fitting this expectation, 5 CAHS loci and a single SAHS locus were up-regulated after slow desiccation, but none were differentially expressed following rapid desiccation. Most *R*. *varieornatus* CAHS and SAHS orthologues had high expression in the active state, several over 1,000 TPM. In contrast, *H*. *dujardini* CAHS and SAHS orthologues had low resting expression (median 0.7 TPM) and were up-regulated (median 1,916.8 TPM) on anhydrobiosis induction. Aquaporins contribute to transportation of water molecules into cells and could be involved in anhydrobiosis [71]. Aquaporin-10 was highly expressed in *R*. *varieornatus* and differentially expressed in anhydrobiotic *H*. *dujardini*. *M*. *tardigradum* has at least 10 aquaporin loci [72], *H*. *dujardini* has 11, and *R*. *varieornatus* has 10. The contributions to anhydrobiosis of additional genes identified as up-regulated (including cytochrome P450, several solute carrier families, and apolipoproteins) are unknown.

Some genes differentially expressed in both *H*. *dujardini* and *R*. *varieornatus* slow-desiccation anhydrobiosis were homologous (S9 Data). Of the 1,422 differentially expressed genes from *H*. *dujardini*, 121 genes were members of 70 protein families that also contained 115 *R*. *varieornatus* differentially expressed genes. These included CAHS, SAHS, glutathione-S transferase, and SOD gene families, but in each case *H*. *dujardini* had more differentially expressed copies than *R*. *varieornatus*. Other differentially expressed gene families were annotated as metalloproteinases, calcium-binding receptors, and G-protein coupled receptors, suggesting that these functions may participate in cellular signaling during induction of anhydrobiosis. Many more (887) gene families included members that were up-regulated by anhydrobiosis in *H*. *dujardini* but unaffected by desiccation in *R*. *varieornatus*. These gene families included 1,879 *R*. *varieornatus* genes; some (154) were highly expressed in the active state (TPM > 100).

In addition to gene loss, we predicted that the tardigrades might have undergone expansion in gene families active in anhydrobiotic physiology. We identified 3 gene families–each containing members with significant differential expression during anhydrobiosis–that had elevated numbers of members in the tardigrades compared to the other taxa analyzed. *H*. *dujardini* and *R*. *varieornatus* had more members of OG000684 (33 and 8, respectively) than any other (mode of 1 and mean of 1.46 copies in the other 28 species, with a maximum of 4 in the moth *Plutella xylostella*). Proteins in OG000684 were annotated with domains associated with ciliar function. OG0002660 contained 3 proteins from *H*. *dujardini* and 3 proteins from *R*. *varieornatus* but a mean of 1.2 from other species. OG0002660 was annotated as fumarylacetoacetase, which acts in phenylalanine metabolism. Fumarylacetoacetase has been identified as a target of the SKN-1-induced stress responses in *C*. *elegans* [73]. OG0002103 was also overrepresented in the tardigrades (3 in each species), while 23 of the other species had 1 copy. Interestingly, the extremophile nematode *Plectus murrayi* had 4 copies. OG0002103 was annotated as guanosine-5'-triphosphate (GTP) cyclohydrolase, involved in formic acid metabolism, including tetrahydrobioterin synthesis. Tetrahydrobioterin is a cofactor of aromatic amino acid hydroxylases, which metabolize phenylalanine. The association of these functions with anhydrobiosis merits investigation.

### Phylogenetic relationships of tardigrada

From the two analyses of protein families shared between *H*. *dujardini*, *R*. *varieornatus*, taxa from other ecdysozoan phyla, and 2 lophotrochozoan outgroup taxa (one that included only taxa with whole genome data, and a second that also included taxa with transcriptome data), we selected putative orthologous protein families. These were screened to eliminate evident paralogous sequences, and alignments were concatenated into a supermatrix. The genomes-only supermatrix included 322 loci from 28 taxa spanning 67,256 aligned residues and had 12.5% missing data. The alignment was trimmed to remove low-quality regions. The genomes and transcriptomes supermatrix included 71 loci from 37 taxa spanning 68,211 aligned residues, had 27% missing data, and was not trimmed. Phylogenomic analyses were carried out in RAxML (using the General Time Reversible model with Gamma distribution of rates model, GTR+G) and PhyloBayes (using a GTR plus rate categories model, GTR-CAT+G). We also explored bipartition support from individual gene trees and RAxML and PhyloBayes analyses of 6-state Dayhoff recoded amino acid alignments using the GTR model (as GTR-CAT cannot be used on these recoded data; S8 Data).

**Fig 4:**
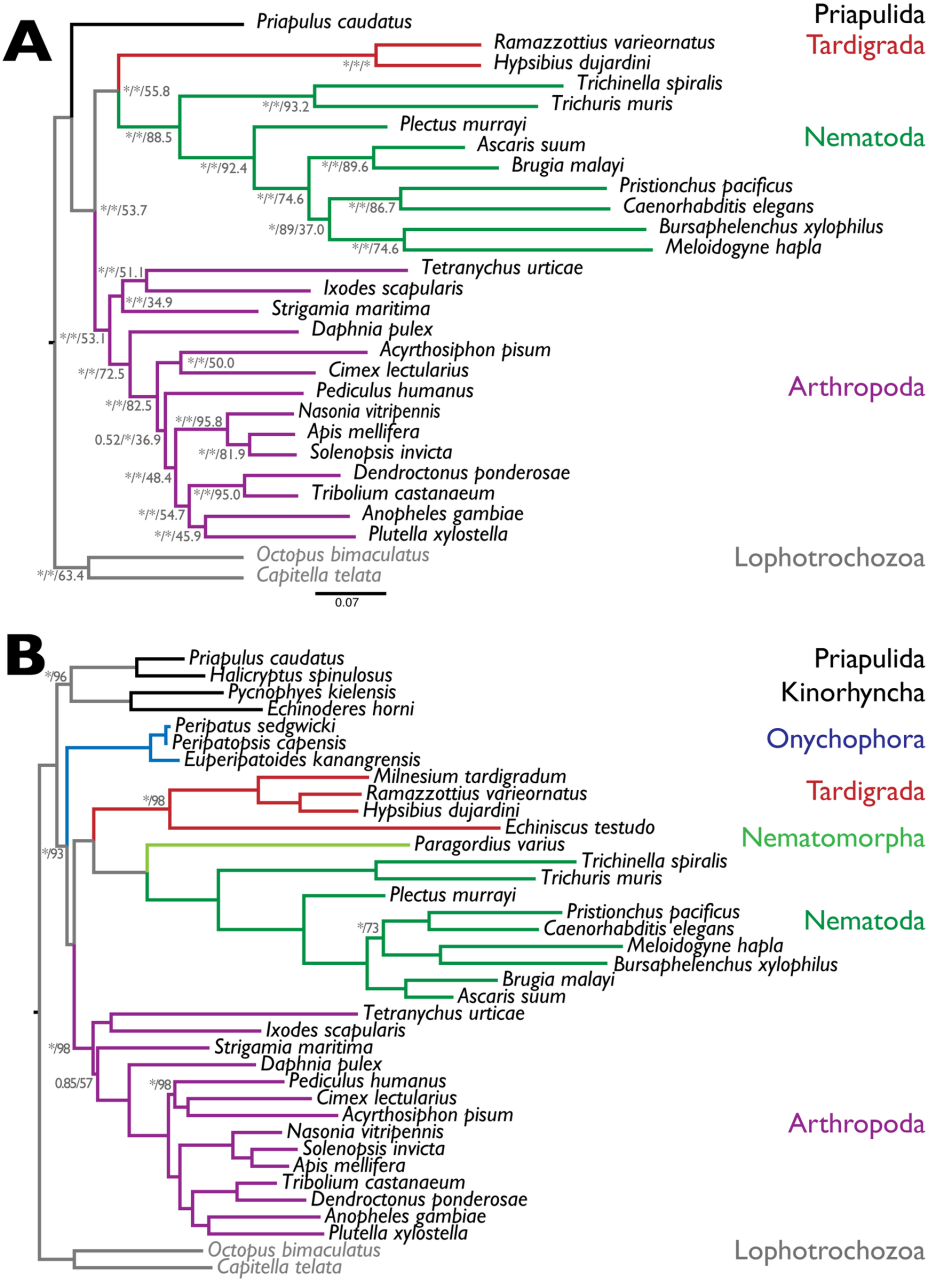
Phylogeny of Ecdysozoa. (A) Phylogeny of 28 species from 5 phyla, based on 322 loci derived from whole genome sequences, and rooted with the lophotrochozoan outgroup. The labels on the nodes are Bayes proportions from PhyloBayes analysis / bootstrap proportions from Randomized Axelerated Maximum Likelihood (RAxML) maximum likelihood bootstraps / proportion of trees of individual loci supporting each bipartition. Note that different numbers of trees were assessed at each node, depending on the representation of the taxa at each locus. * indicates maximal support (Bayes proportion of 1.0 or RAxML bootstrap of 1.0). (B) Phylogeny of 36 species from 8 phyla, based on 71 loci derived using PhyloBayes from whole genome and transcriptome sequences, and rooted with the lophotrochozoan outgroup. All nodes had maximal support in Bayes proportions and RAxML bootstrap, except those labeled (Bayes proportion, * = 1.0 / RAxML bootstrap).

The genomes-only phylogeny (Fig 4A) strongly supported Tardigrada as a sister to monophyletic Nematoda. Within Nematoda and Arthropoda, the relationships of species were congruent with previous analyses, and the earliest branching taxon in Ecdysozoa was Priapulida. RAxML bootstrap and PhyloBayes Bayes proportion support was high across the phylogeny, with only 2 internal nodes in Nematoda and Arthropoda receiving less-than-maximal support. Analysis of individual RAxML phylogenies derived from the 322 loci revealed a degradation of support deeper in the tree, with 53% of trees supporting a monophyletic Arthropoda, 56% supporting Tardigrada plus Nematoda, and 54% supporting the monophyly of Arthropoda plus Tardigrada plus Nematoda. The phylogeny derived from the genomes and transcriptomes dataset (Fig 4B) also recovered credibly resolved Nematoda and Arthropoda and, as expected, placed Nematomorpha as sister to Nematoda. Tardigrada was again recovered as sister to Nematoda plus Nematomorpha, with maximal support. Priapulida plus Kinorhyncha was found to arise basally in Ecdysozoa. Unexpectedly, Onychophora, represented by 3 transcriptome datasets, was sister to an Arthropoda plus (Tardigrada, Nematomorpha, and Nematoda) clade, again with high support.

### Rare genomic changes and tardigrade relationships

We tested support for a Nematoda+Tardigrada clade in rare genomic changes [74] in core developmental gene sets and protein family evolution. Rare genomic changes can be used as strong parsimony markers of phylogenetic relationships that are hard to resolve using model-based sequence analyses. An event shared by 2 taxa can be considered to support their relationship where the likelihood of the event is a priori expected to be vanishingly small.

**Fig 5:**
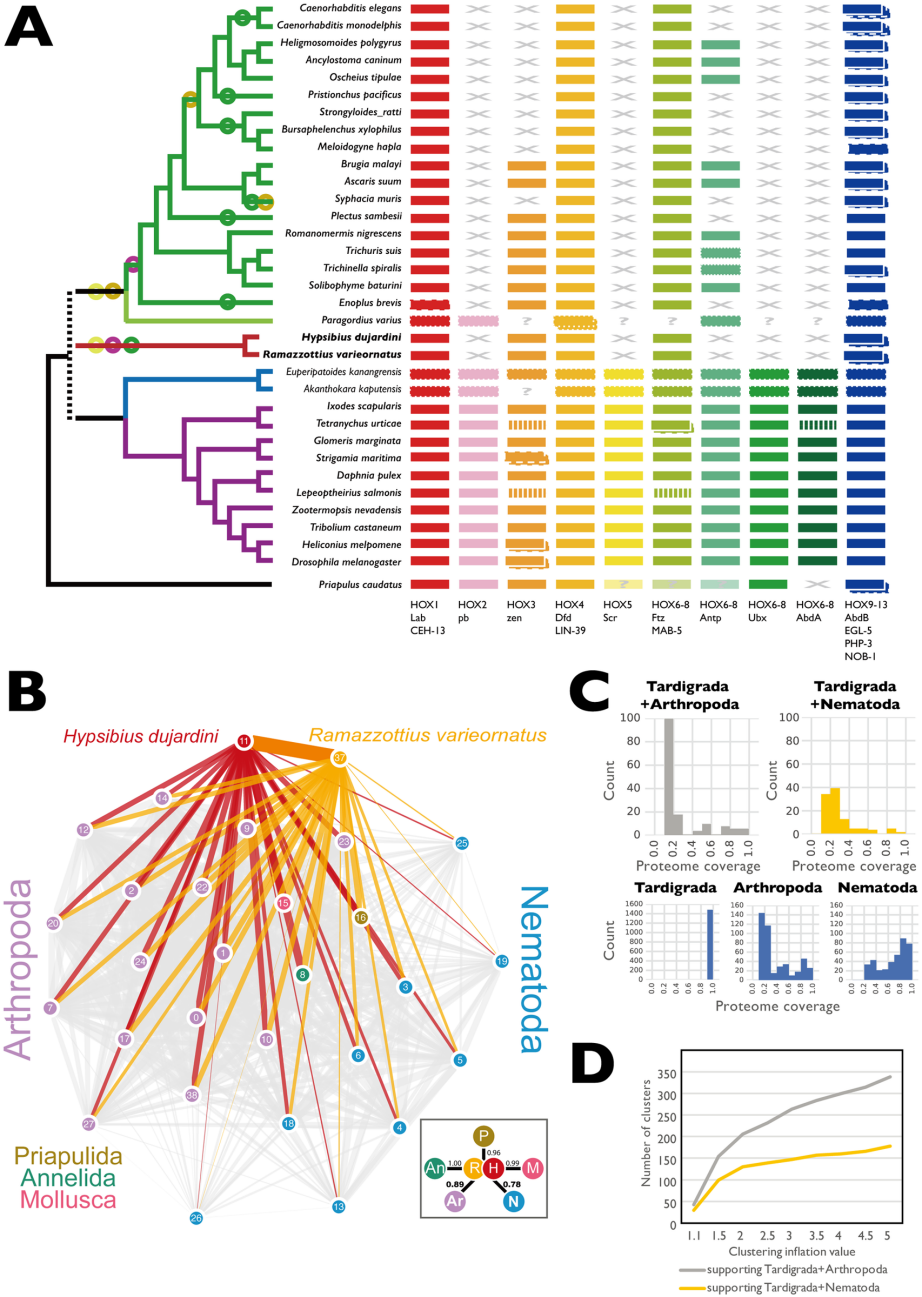
The position of tardigrada in Ecdysozoa. (A) HOX genes in tardigrades and other Ecdysozoa. HOX gene losses in Tardigrada and Nematoda. HOX gene catalogues of tardigrades and other Ecdysozoa were collated by screening ENSEMBL Genomes and WormBase Parasite. HOX orthology groups are indicated by different colors. Some “missing” HOX loci were identified by Basic Local Alignment Search Tool (BLAST) search of target genomes (indicated by vertical striping of the affected HOX). “?” indicates that presence/absence could not be confirmed because the species was surveyed by PCR or transcriptomics; loci identified by PCR or transcriptomics are indicated by a dotted outline. “X” indicates that orthologous HOX loci were not present in the genome of that species. Some species have duplications of loci mapping to 1 HOX group, and these are indicated by boxes with dashed outlines. The relationships of the species are indicated by the cladogram to the left, and circles on this cladogram indicate Dollo parsimony mapping of events of HOX group loss on this cladogram. Circles are colored congruently with the HOX loci. (B) Evolution of gene families under different hypotheses of tardigrade relationships. Tardigrades share more gene families with Arthropoda than with Nematoda. In this network, derived from the OrthoFinder clustering at inflation value 1.5, nodes represent species (0: *Anopheles gambiae*, 1: *Apis mellifera*, 2: *Acyrthosiphon pisum*, 3: *Ascaris suum*, 4: *Brugia malayi*, 5: *Bursaphelenchus xylophilus*, 6: *Caenorhabditis elegans*, 7: *Cimex lectularius*, 8: *Capitella teleta*, 9: *Dendroctonus ponderosae*, 10: *Daphnia pulex*, 11: *Hypsibius dujardini*, 12: *Ixodes scapularis*, 13: *Meloidogyne hapla*, 14: *Nasonia vitripennis*, 15: *Octopus bimaculoides*, 16: *Priapulus caudatus*, 17: *Pediculus humanus*, 18: *Plectus murrayi*, 19: *Pristionchus pacificus*, 20: *Plutella xylostella*, 37: *Ramazzottius varieornatus*, 22: *Solenopsis invicta*, 23: *Strigamia maritima*, 24: *Tribolium castaneum*, 25: *Trichuris muris*, 26: *Trichinella spiralis*, 27: *Tetranychus urticae*, 38: *Drosophila melanogaster*). The thickness of the edge connecting 2 nodes is weighted by the count of shared occurrences of both nodes in OrthoFinder-clusters. Links involving *H*. *dujardini* (red) and *R*. *varieornatus* (orange) are colored. The inset box on the lower right shows the average weight of edges between each phylum and both Tardigrades, normalized by the maximum weight (i.e., count of co-occurrences of Tardigrades and the annelid *C*. *teleta*)" (C) Gene family birth synapomorphies at key nodes in Ecdysozoa under 2 hypotheses: Tardigrada+Nematoda versus Tardigrada+Arthropoda. Each graph shows the number of gene families at the specified node inferred using Dollo parsimony from OrthoFinder clustering at inflation value 1.5. Gene families are grouped by the proportion of taxa above that node that contain a member. Note that to be included as a synapomorphy of a node, a gene family must contain proteins of at least 1 species of each child node of the node in question, and thus, there are no synapomorphies with <0.3 proportional proteome coverage in Nematoda and <0.2 in Arthropoda, and all synapomorphies of Tardigrada have 1.0 representation. (D) Gene family birth synapomorphies for Tardigrada+Arthropoda (grey) and Tardigrada+Nematoda (yellow) for OrthoFinder clusterings performed at different Markov Cluster Algorithm (MCL) inflation parameters.

HOX genes are involved in anterior-posterior patterning across the Metazoa, with a characteristic set of paralogous genes present in most animal genomes, organized as a tightly regulated cluster. The ancestral cluster is hypothesized to have included HOX1, HOX2, HOX3, HOX4, HOX5, and a HOX6-8 like locus and HOX9. The HOX6-8 and HOX9 types have undergone frequent, independent expansion and contraction during evolution, and HOX clustering has broken down in some species. HOX complements are generally conserved between related taxa, and gain and loss of HOX loci can be considered a rare genomic change. We surveyed HOX loci in tardigrades and relatives (Fig 5A). In the priapulid *Priapulus caudatus*, 9 HOX loci have been described [75], but no HOX6-8/*AbdA*-like gene was identified. All arthropods surveyed (including representatives of the 4 classes) had a complement of HOX loci very similar to that of *D*. *melanogaster*, with at least 10 loci including HOX6-8 and HOX9. Some HOX loci in some species have undergone duplication, particularly HOX3/*zen*. In the mite *Tetranychus urticae* and the salmon louse *Lepeoptheirius salmonis*, we identified “missing” HOX genes in the genome. For Onychophora, the sister group to Arthropoda, HOX loci have only been identified through PCR screens [76,77], but they appear to have the same complement as Arthropoda.

In *H*. *dujardini*, a reduced HOX gene complement (6 genes in 5 orthology groups) has been reported [78], and we confirmed this reduction using our improved genome (Fig 5A). The same reduced complement was also found in the genome of *R*. *varieornatus* [22], and the greater contiguity of the *R*. *varieornatus* genome shows that 5 of the 6 HOX loci are on 1 large scaffold, distributed over 2.7 Mb, with 885 non-HOX genes separating them. The *H*. *dujardini* loci were unlinked in our assembly, except for the 2 HOX9/*AbdB*-like loci, and lack of gene level synteny precludes ordering of these scaffolds based on the *R*. *varieornatus* genome. The order of the HOX genes on the *R*. *varieornatus* scaffolds is not colinear with other, unfragmented clusters, as HOX6-8/*ftz* and the pair of HOX9/*AbdB* genes are inverted, and HOX4/*dfd* is present on a second scaffold (and not found between HOX3 and HOX6-8/*ftz* as would be expected).

The absences of HOX2/*pb*, HOX5/*scr*, and HOX6-8/*Ubx/AbdA* in both tardigrade species is reminiscent of the situation in Nematoda, in which these loci are also absent [79–81]. HOX gene evolution in Nematoda has been dynamic. No Nematode HOX2 or HOX5 orthology group genes were identified, and only a few species had a single HOX6-8 orthologue. Duplication of the HOX9/*AbdB* locus was common, generating, for instance, the *egl-5*, *php-3*, and *nob-1* loci in *Caenorhabditis* species. The maximum number of HOX loci in a nematode was 7, deriving from 6 orthology groups. Loss of HOX3 happened twice (in *Syphacia muris* and in the common ancestor of Tylenchomorpha and Rhabditomorpha). The independent loss in *S*. *muris* was confirmed in 2 related pinworms, *Enterobius vermicularis* and *Syphacia oblevata*. The pattern of presence and absence of the *Antp*-like HOX6-8 locus is more complex, requiring 6 losses (in the basally arising enoplean *Enoplis brevis*, the chromadorean *Plectus sambesii*, the pinworm *S*. *muris*, the ancestor of Tylenchomorpha, the diplogasteromorph *Pristionchus pacificus*, and the ancestor of *Caenorhabditis*). We affirmed loss in the pinworms by screening the genomes of *E*. *vermicularis* and *S*. *oblevata* as above, and no HOX6-8/*Antp*-like locus was present in any of the over 20 genomes available for *Caenorhabditis*. A PCR survey for HOX loci and screening of a de novo assembled transcriptome from the nematomorph *Paragordius varius* identified 6 putative loci from 5 HOX groups. The presence of a putative HOX2/*pb*-like gene suggests that loss of HOX2 may be independent in Tardigrada and Nematoda.

Gene family birth can be used as another rare genomic marker. We analyzed the whole proteomes of ecdysozoan taxa for gene family births that supported either the Tardigrada+Nematoda model or the Tardigrada+Arthropoda (i.e., Panarthropoda) model. We mapped gene family presence and absence across the 2 contrasting phylogenies using KinFin [63] using different inflation parameters in the Markov Cluster Algorithm (MCL) step in OrthoFinder (S10 Data). Using the default inflation value of 1.5, the 2 tardigrades shared more gene families with Arthropoda than they did with Nematoda (Fig 5B). The numbers of gene family births synapomorphic for Arthropoda and Nematoda were identical under both phylogenies, as was expected (Table 3; Fig 5C; S11 Data). Many synapomorphic families had variable presence in the daughter taxa of the common ancestors of Arthropoda and Nematoda, likely because of stochastic gene loss or lack of prediction. However, especially in Nematoda, most synapomorphic families were present in a majority of species (Fig 5C).

**Table 3:** Gene family births that support different relationships of Tardigrada.

Protein family*	Number of proteins	Proportion of proteomes represented				Domain annotations**
		All	Nematoda (*n* = 9)	Arthropoda (*n* = 15)	Tardigrada (*n* = 2)	
**Synapomorphies with membership ≥ 0.7 under the Panarthropoda (Tardigrada+Arthropoda) hypothesis**						
**OG0000436**	104	1.00	0.00	1.00	1.00	Serine proteases, trypsin domain (IPR001254)
**OG0001236**	54	1.00	0.00	1.00	1.00	Major facilitator superfamily-associated domain (IPR024989)
**OG0002592**	36	1.00	0.00	1.00	1.00	Spätzle (IPR032104)
**OG0006538**	19	1.00	0.00	1.00	1.00	Leucine-rich repeat (IPR001611)
**OG0006541**	19	1.00	0.00	1.00	1.00	None
**OG0006869**	17	1.00	0.00	1.00	1.00	Thioredoxin domain (IPR013766)
**OG0005117**	27	0.88	0.00	0.93	0.50	BTB/POZ domain (IPR000210)
**OG0005941**	22	0.77	0.00	0.73	1.00	None
**OG0006662**	18	0.82	0.00	0.80	1.00	None
**OG0006889**	17	0.71	0.00	0.73	0.50	None
**OG0006940**	17	0.82	0.00	0.87	0.50	EGF-like domain (IPR000742), Laminin G domain (IPR001791)
**OG0006941**	17	0.71	0.00	0.67	1.00	EF-hand domain (IPR002048)
**OG0006951**	17	0.71	0.00	0.67	1.00	Adipokinetic hormone (IPR010475)
**OG0007141**	16	0.82	0.00	0.80	1.00	None
**OG0007285**	15	0.71	0.00	0.67	1.00	GPCR, family 2, secretin-like (IPR000832)
**OG0007290**	15	0.82	0.00	0.80	1.00	Allatostatin (IPR010276)
**OG0007298**	15	0.88	0.00	0.87	1.00	None
**OG0007328**	15	0.71	0.00	0.67	1.00	Sulfakinin (IPR013259)
**OG0007463**	14	0.77	0.00	0.73	1.00	Peptidase S1A, nudel (IPR015420), serine proteases, trypsin domain (IPR001254), low-density lipoprotein (LDL) receptor class A repeat (IPR002172)
**OG0007689**	13	0.71	0.00	0.67	1.00	Marvel domain (IPR008253)
**Synapomorphies with membership ≥ 0.7 under the Tardigrada+Nematoda hypothesis**						
**OG0005423**	26	0.82	0.89	0.00	0.50	Amidinotransferase (PF02274)
**OG0006414**	20	0.82	0.78	0.00	1.00	Proteolipid membrane potential modulator (IPR000612)
**OG0007199**	16	0.91	1.00	0.00	0.50	Zona pellucida domain (IPR001507)
**OG0007812**	13	0.82	0.78	0.00	1.00	None
**OG0008368**	11	0.82	0.78	0.00	1.00	RUN domain (IPR004012)

* Protein families from OrthoFinder clustering at inflation value 1.5.

** Domain annotations are reported where proteins from more than one-third of the proteomes in the family had that annotation.

EGF, epidermal growth factor; GPCR, G-protein-coupled receptor; IPR, InterPro domain identifier; PF, PFam identifier.

At inflation value 1.5, we found 6 gene families present that had members in both tardigrades and all 14 arthropods under Panarthropoda, but no gene families were found in both tardigrades and all 9 nematodes under the Tardigrada+Nematoda hypothesis (S10 Table). Allowing for stochastic absence, we inferred 154 families to be synapomorphic for Tardigrada+Arthropoda under the Panarthropoda hypothesis, and 99 for Tardigrada+Nematoda under the Tardigrada+Nematoda hypothesis (Fig 5D). More of the Tardigrada+Arthropoda synapomorphies had higher species representation than did the Tardigrada+Nematoda synapomorphies. This pattern was also observed in analyses using different inflation values and in analyses including the transcriptome from the nematomorph *P*. *varius*.

We explored the biological implications of these putative synapomorphies by examining the functional annotations of each protein family that contained members from ≥70% of the ingroup species (Table 3). Under Tardigrada+Arthropoda, 20 families had ≥70% of the ingroup taxa represented, and 6 were universally present. These included important components of developmental and immune pathways, neuromodulators, and others. Two families were annotated as serine endopeptidases, 1 missing in some arthropods that included *D*. *melanogaster* Nudel and 1 found in all species. Another synapomorphic family, found in all species, included *spätzle* orthologues. Spätzle is a cysteine-knot, cytokine-like ligand involved in dorsoventral patterning and is the target of a serine protease activation cascade initiated by Nudel protease. The identification of more than 1 member of a single regulatory cascade as potential gene family births suggests that the pathway may have been established in a Tardigrada+Arthropoda most recent common ancestor. Other Tardigrada+Arthropoda-synapomorphic families were annotated with ommatidial apical extracellular matrix (eyes shut), adipokinetic hormone, neuromodulatory allatostatin-A, drosulfakinin, leucine-rich repeat, thioredoxin, major facilitator superfamily associated, and domain of unknown function DUF4728 domains. However, 9 of the 20 Panarthropoda synapomorphic families had no informative domain annotations. Under Tardigrada+Nematoda, only 5 putatively synapomorphic families had members from ≥70% of the ingroup species. Four of these had domain matches (proteolipid membrane potential modulator, zona pellucida, RUN, and amidinotransferase domains), and 1 contained no proteins with identifiable domains.

## Discussion

### A robust estimate of the *H*. *dujardini* genome

We have sequenced and assembled a high-quality genome for the tardigrade *H*. *dujardini*, utilizing new data, including single-molecule, long-read sequencing, and heterozygosity-aware assembly methods. Comparison of genomic metrics with previous assemblies for this species showed that our assembly is more complete and more contiguous than has been achieved previously and retains minimal uncollapsed heterozygous regions. The span of this new assembly is much closer to independent estimates of the size of the *H*. *dujardini* genome (75–100 Mb) using densitometry and staining. The *H*. *dujardini* genome is thus nearly twice the size of that of the related tardigrade *R*. *varieornatus*. We compared the 2 genomes to identify differences that would account for the larger genome in *H*. *dujardini*. While *H*. *dujardini* had approximately 6,000 more protein coding genes than *R*. *varieornatus*, these accounted for only approximately 23 Mb of the additional span and are not obviously simple duplicates of genes in *R*. *varieornatus*. Analyses of the gene contents of the 2 species showed that while *H*. *dujardini* had more species-specific genes, it also had greater numbers of loci in species-specific gene family expansions than *R*. *varieornatus* and had lost fewer genes whose origins could be traced to a deeper ancestor. *H*. *dujardini* genes had, on average, the same structure (approximately 6 exons per gene) as did *R*. *varieornatus*; however, introns in *H*. *dujardini* genes were on average twice the length of their orthologues in *R*. *varieornatus* (255 bases versus 158 bases). Finally, the *H*. *dujardini* genome was more repeat rich (28.5% compared to only 21% in *R*. *varieornatus*). These data argue against simple whole genome duplication in *H*. *dujardini*. The genome of *H*. *dujardini* is larger because of expansion of noncoding DNA, including repeats and introns, and acquisition and retention of more new genes and gene duplications than *R*. *varieornatus*. The disparity in retention of genes with orthologues outside the Tardigrada, where *R*. *varieornatus* has lost more genes than has *H*. *dujardini*, suggests that *R*. *varieornatus* may have undergone genome size reduction and that the ancestral tardigrade (or hypsibiid) genome is more likely to have been approximately 100 Mb than 54 Mb. We await additional tardigrade genomes with interest. While we identified linkage between genes in the 2 tardigrades, local synteny was relatively rare. In this, these genomes resemble those of the genus *Caenorhabditis*, in which extensive, rapid, within-chromosome rearrangement has served to break close synteny relationships while, in general, maintaining linkage [82]. The absence of chromosomal level assemblies for either tardigrade (and lack of any genetic map information) precludes definitive testing of this hypothesis.

### HGT in tardigrades: *H*. *dujardini* has a normal metazoan genome

Boothby et al. made the surprising assertion that 17.5% of *H*. *dujardini* genes originated through HGT from a wide range of bacterial, fungal, and protozoan donors [19]. Subsequently, several groups including our teams proved that this finding was the result of contamination of their tardigrade samples with cobionts and less-than-rigorous screening of HGT candidates [20,21,39,40]. We found that the use of uncurated gene-finding approaches yielded elevated HGT proportion estimates in many other nematode and arthropod genomes, even where contamination is unlikely to have been an issue. It is thus essential to follow up initial candidate sets of HGT loci with detailed validation. We screened our new *H*. *dujardini* assembly for evidence of HGT, identifying a maximum of 2.3% of the protein coding genes as potential candidates. After careful assessment using phylogenetic analysis and expression evidence, we identified a likely high-confidence set of only 0.7% of *H*. *dujardini* genes that originated through HGT. HGT was also low (1.6%) in the high-quality *R*. *varieornatus* genome. These proportions are congruent with similar analyses of *C*. *elegans* and *D*. *melanogaster*. Curation of the genome assemblies and gene models may decrease the proportion further. Tardigrades do not have elevated levels of HGT.

While tardigrades do not have elevated levels of HGT in their genomes, some HGT events are of importance in anhydrobiosis. All *H*. *dujardini* catalase loci were of bacterial origin, as described for *R*. *varieornatus* [22]. While trehalose phosphatase synthase was absent from *H*. *dujardini*, *R*. *varieornatus* has a TPS that likely was acquired by HGT (S5 Data). As *M*. *tardigradum* does not have a TPS homologue, while other ecdysozoan taxa do, this suggests that TPS may have been lost in the common ancestor of eutardigrada and regained in *R*. *varieornatus* by HGT after divergence from *H*. *dujardini*.

### Contrasting modes of anhydrobiosis in tardigrades

Genes with likely roles in protection against extreme stress previously identified in *R*. *varieornatus* were largely conserved in *H*. *dujardini*. Both CAHS and SAHS families had high copy numbers in both species, with independent expansions. However, we did not find a Dsup orthologue in *H*. *dujardini*. *H*. *dujardini* has similar gene losses to *R*. *varieornatus* in pathways that produce reactive oxygen species (ROS) and in cellular stress signaling pathways, which suggest that the gene losses occurred before the divergence of the 2 species. This loss of important signaling pathway genes may disconnect signals of stress induction from activating downstream response systems that must be suppressed if anhydrobiosis is to be achieved successfully—for example, cell cycle regulation, transcription and replication inhibition, and apoptosis. As cellular protection and repair pathways were highly conserved, damaged cell systems will still be protected and repaired. Indeed, some stress-related gene families had undergone gene family expansion in 1 or both tardigrades. SOD was duplicated in both species, as was a calcium-activated potassium channel, which has been implicated in cellular signaling during anhydrobiosis [23]. The elevated gene family expansion in *H*. *dujardini* compared to *R*. *varieornatus* may be related to retention and expansion of induced stress response systems.

The transcriptome response to anhydrobiosis differs between the 2 tardigrades. *H*. *dujardini* has an induced transcriptomic response where *R*. *varieornatus* does not. We found that *H*. *dujardini* had more genes differentially expressed on anhydrobiosis than *R*. *varieornatus*. As anticipated, more *R*. *varieornatus* loci were differentially expressed when desiccated at a slow pace. Genes induced by slow desiccation included CAHS and SAHS genes and antioxidant-related genes. Although most of these genes were highly expressed (>100 TPM) in the active state, the induction of these genes may enable higher recovery. CAHS and SAHS loci were also overexpressed on anhydrobiosis in *H*. *dujardini*. We found a variety of calcium-related transporters and receptors were differentially expressed on anhydrobiosis. Kondo et al. suggested that cellular signaling using calmodulin and calcium may be required for anhydrobiosis [23], but it is still unclear how this is related to anhydrobiosis. Calcium and other metal ion concentrations could be increased during dehydration and thus could act as a desiccation signal. Trehalose is known for its role in protecting cellular systems from dehydration [35,36,83,84]. It has been hypothesized that it may not be required for tardigrade anhydrobiosis, as trehalose was not detected in *M*. *tardigradum* [31]. Trehalose synthesis via TPS has been lost in *H*. *dujardini*, although we found an HGT-origin TPS in *R*. *varieornatus*. Unexpectedly, 3 *R*. *varieornatus* trehalase loci were differentially expressed on slow desiccation, including 2 with over 200 TPM in the anhydrobiotic state. As trehalose degradation should not be required in the absence of trehalose, there may be an alternative pathway for trehalose synthesis.

### The position of tardigrades in the metazoa

Our phylogenomic analyses found Tardigrada, represented by *H*. *dujardini* and *R*. *varieornatus* genomes as well as transcriptomic data from *M*. *tardigradum* and *Echiniscus testudo*, to be sisters to Nematoda, not Arthropoda. This finding was robust to inclusion of additional phyla, such as Onychophora and Nematomorpha, and to filtering the alignment data to exclude poorly represented or rapidly evolving loci. This finding is both surprising and not new. Many previous molecular analyses have found Tardigrada to group with Nematoda, whether using single genes or ever larger gene sets derived from transcriptome and genome studies [1–3]. This phenomenon has been attributed to long branch attraction in suboptimal datasets, with elevated substitutional rates or biased compositions in Nematoda and Tardigrada mutually and robustly driving Bayesian and Maximum Likelihood algorithms to support the wrong tree. Strikingly, in our analyses including taxa for which transcriptome data are available, we found Onychophora to lie outside a ([Nematoda, Nematomorpha, and Tardigrada], Arthropoda) clade. This finding, while present in some other analyses (e.g., component phylogenies summarized in [2]), conflicts with accepted systematic and many molecular analyses. We note that Onychophora was only represented by transcriptome datasets and that there is accordingly an elevated proportion of missing data in the alignment for this phylum.

Developmental and anatomical data do not, in general, support a tree linking Tardigrada with Nematoda. Tardigrades are segmented, have appendages, and have a central and peripheral nervous system anatomy that can be homologized with those of Onychophora and Arthropoda [85, 86]. In contrast, nematodes are unsegmented, have no lateral appendages, and have a simple nervous system. The myoepithelial triradiate pharynx, found in Nematoda, Nematomorpha, and Tardigrada, is 1 possible morphological link, but Nielsen has argued persuasively that the structures of this organ in nematodes and tardigrades (and other taxa) are distinct and thus nonhomologous [5].

*H*. *dujardini* has a reduced complement of HOX loci, as does *R*. *varieornatus*. Some of the HOX loci missing in the Tardigrada are the same as those absent from Nematoda. Whether these absences are a synapomorphy for a Nematode-Tardigrade clade or simply a product of homoplasious evolution remains unclear. It may be that miniaturization of Nematoda and Tardigrada during adaptation to life in interstitial habitats facilitated the loss of specific HOX loci involved in postcephalic patterning and that both nematodes and tardigrades can be thought to have evolved by reductive evolution from a more fully featured ancestor. It may be intrinsically easier to lose some HOX loci than others. While tardigrades retain obvious segmentation, nematodes do not, with the possible exception of repetitive cell lineages along the anterior-posterior axis during development [87]. We note that until additional species were analyzed, the pattern observed in *C*. *elegans* was assumed to be the ground pattern for all Nematoda. More distantly related Tardigrada may have different HOX gene complements to these hypsibiids, and a pattern of staged loss similar to that in Nematoda [79–81] may be found.

Assessment of gene family births as rare genomic changes lent support to a Tardigrada+Arthropoda clade, but the support was not striking. There were more synapomorphic gene family births when a Tardigrada+Arthropoda (Panarthropoda) clade was assumed than when a Tardigrada+Nematoda clade was assumed. However, analyses under the assumption of Tardigrada+Nematoda identified synapomorphic gene family births at 50% of the level found when Panarthropoda was assumed. We note that recognition of gene families may be compromised by the same “long branch attraction” issues that plague phylogenetic analyses and also that any taxon in which gene loss is common (such as has been proposed for Nematoda as a result of its simplified body plan) may score poorly in gene family membership metrics. The short branch lengths that separate basal nodes in the analysis of the panarthropodan-nematode part of the phylogeny of Ecdysozoa may make robust resolution very difficult. We explored the biological implications of the synapomorphies that supported Panarthropoda by examining the functional annotations of each protein family (S10 Table) and were surprised that many of these deeply conserved loci have escaped experimental, genetic, or biochemical annotation. One family included *spätzle*, a cysteine-knot, cytokine-like family involved in dorsoventral patterning as well as immune response, and 2 others were serine endopeptidases, including *nudel*, which is part of the same pathway as *spätzle*. This pathway may be a Panarthropod innovation. Thus, our analyses of rare genomic changes lent some support to the Panarthropoda hypothesis, as did analysis of miRNA gene birth [2], but analysis of HOX loci may conflict with this.

The question of tardigrade relationships remains open [4]. While we found support for a clade of Tardigrada, Onychophora, Arthropoda, Nematoda, and Nematomorpha, the branching order within this group remains contentious, and in particular, the positions of Tardigrada and Onychophora are poorly supported and/or variable in our and others’ analyses. Full genome sequences of representatives of Onychophora, Heterotardigrada (the sister group to the Eutardigrada including Hypsibiidae), Nematomorpha, and enoplian, basally arising Nematoda are required. Resolution of the conflicts between morphological and molecular data will be informative, either of the ground state of a nematode-tardigrade ancestor or of the processes that drive homoplasy in rare genomic changes and robust discovery of nonbiological trees in sequence-based phylogenomic studies.

## Methods

### Tardigrade culture and sampling

The tardigrade *H*. *dujardini* Z151 was purchased from Sciento (Manchester, United Kingdom). *H*. *dujardini* Z151 and *R*. *varieornatus* strain YOKOZUNA-1 were cultured as previously described [24,42]. Briefly, tardigrades were fed *Chlorella vulgaris* (Chlorella Industry) on 2% Bacto Agar (Difco) plates prepared with Volvic water, incubated at 18°C for *H*. *dujardini* and 22°C for *R*. *varieornatus* under constant dark conditions. Culture plates were renewed approximately every 7–8 d. Anhydrobiotic adult samples were isolated on 30 μM filters (Millipore) and placed in a chamber maintained at 85% RH for 48 h for *H*. *dujardini*; 30% RH for 24 h and additional 24 h at 0% RH for slow-dried *R*. *varieornatus*; and 0% RH for 30 min on a 4 cm x 4 cm Kim-towel with 300 μL of distilled water and an additional 2 h without the towel for fast-dried *R*. *varieornatus*. Successful anhydrobiosis was assumed when >90% of the samples prepared in the same chamber recovered after rehydration.

### Sequencing

Genomic DNA for long read sequencing was extracted using MagAttract HMW DNA Kit (Qiagen) from approximately 900,000 *H*. *dujardini*. DNA was purified twice with AMPure XP beads (Beckman Coulter). A 20 kb PacBio library was prepared following the manual “20 kb Template Preparation Using BluePippin Size-Selection System (15 kb Size Cutoff)” (PacBio SampleNet—Shared Protocol) using SMARTBell Template Prep Kit 1.0 (Pacific Biosciences) and was sequenced using 8 SMRT Cells Pac V3 with DNA Sequencing Reagent 4.0 on a PacBio RSII System (Pacific Biosciences) at Takara Bio. Briefly, purified DNA was sheared, targeting 20 kb fragments, using a g-TUBE (Covaris). Following end-repair and ligation of SMRTbell adapters, the library was size selected using BluePippin (Sage Science) with a size cutoff of 10 kb. The size distribution of the library was assayed on TapeStation 2200 (Agilent) and quantified using the Quant-iT dsDNA BR Assay Kit (Invitrogen). MiSeq reads from a single *H*. *dujardini* individual (DRR055040) and HiSeq reads are from our previous reports [20,21].

For mRNA-Seq to be used in genome annotation, 30 individuals were collected from each of the following conditions in 3 replicates: active and dried adults (slow dried for *R*. *varieornatus*), eggs (1, 2, 3, 4, 5, 6, and 7 d after laying), and juveniles (1, 2, 3, 4, 5, 6, and 7 d after hatching). Because of sample preparations, *R*. *varieornatus* juveniles were sampled until juvenile first day. For gene expression analysis, we sampled approximately 2–3 individuals of fast-dried *R*. *varieornatus*. All mRNA-Seq analyses were conducted with 3 replicates. Specimens were thoroughly washed with Milli-Q water on a sterile nylon mesh (Millipore) and immediately lysed in TRIzol reagent (Life Technologies) using 3 cycles of immersion in liquid nitrogen followed by 37°C incubation. Total RNA was extracted using the Direct-zol RNA kit (Zymo Research) following the manufacturer’s instructions, and RNA quality was checked using the High Sensitivity RNA ScreenTape on a TapeStation (Agilent Technologies). For library preparation, mRNA was amplified using the SMARTer Ultra Low Input RNA Kit for Sequencing v.4 (Clontech), and Illumina libraries were prepared using the KAPA HyperPlus Kit (KAPA Biosystems). Purified libraries were quantified using a Qubit Fluorometer (Life Technologies), and the size distribution was checked using the TapeStation D1000 ScreenTape (Agilent Technologies). Libraries size selected above 200 bp by manually excision from agarose were purified with a NucleoSpin Gel and PCR Clean-up kit (Clontech). The samples were then sequenced on the Illumina NextSeq 500 in High Output Mode with a 75-cycle kit (Illumina) as single end reads, with 48 multiplexed samples per run. Adapter sequences were removed, and the sequences were demultiplexed using the bcl2fastq v.2 software (Illumina). For active and dried adults, RNA-Seq was also conducted starting from approximately 10,000 individuals, similarly washed, but RNA extraction was conducted with TRIzol reagent (Life Technologies) followed by RNeasy Plus Mini Kit (Qiagen) purification. Library preparation and sequencing were conducted at Beijing Genomics Institute.

For miRNA-Seq, 5,000 individuals were homogenized using Biomasher II (Funakoshi), and TRIzol (Invitrogen) was used for RNA extraction; the individuals were then purified by isopropanol precipitation. Size selection of fragments of 18–30 nt using electrophoresis, preparation of the sequencing library for Illumina HiSeq 2000, and subsequent (single end) sequencing were carried out by Beijing Genomics Institute.

All sequence data were validated for quality using FastQC [88].

### Genome assembly

The MiSeq reads from whole genome amplified DNA were merged with Usearch [89], and both merged and unmerged pairs were assembled with SPAdes [90] as single end. The SPAdes assembly was checked for contamination with BLAST+ BLASTN [68] against the nr [91] database, and no observable contamination was found with blobtools [46]. Illumina data from Boothby et al. [19] were mapped to the SPAdes assembly with Bowtie2 [92], and read pairs were retained if at least 1 of them mapped to the assembly. These reads were then assembled, scaffolded, and gap closed with Platanus [44]. The Platanus assembly was further scaffolded and gap closed using the PacBio data with PBJelly [93].

Falcon [43] assembly of PacBio data was performed on the DNAnexus platform. Using this Falcon assembly, Platanus assembly was extended using SSPACE-LongReads [47] and gap-filled with PBJelly [93] with default parameters. Single-individual MiSeq reads were mapped to the assembly with BWA, and all contigs with coverage < 1 or length < 1,000 bp or those corresponding to the mitochondrial genome were removed. At this stage, 1 CEGMA gene became unrecognized by CEGMA [48], probably because of multiple PBJelly runs, and therefore, the contig harboring that missing CEGMA gene was corrected by Pilon [94] using the single individual MiSeq reads. We also validated genomic completeness with BUSCO using the eukaryote lineage gene set.

### Gene finding

mRNA-Seq data (Development, Active-tun 10k animals) were mapped to the genome assembly with TopHat [92,95] without any options. Using the mapped data from TopHat, BRAKER [54] was used with default settings to construct a GeneMark-ES [96] model and an Augustus [64] gene model, which are used for ab initio prediction of genes. The getAnnotFasta.pl script from Augustus was used to extract coding sequences from the GFF3 file. Similarly, to construct a modified version of the *R*. *varieornatus* genomes annotation, we used the development and anhydrobiosis (S1 Table) RNA-Seq data for BRAKER annotation. We found that a few genes were misannotated (MAHS in both species, a CAHS orthologue in *R*. *varieornatus*), due to fusion with a neighboring gene, and these were manually curated. tRNA and rRNA genes were predicted with tRNAscan-SE [51] and RNAmmer [50], respectively. BUSCO was used again to validate the completeness of the predicted gene set for both tardigrades.

The mRNA-Seq data used to predict the gene models were mapped with BWA MEM [97] against the predicted coding sequences, the genome, and a Trinity [56] assembled transcriptome. We also mapped the mRNA-Seq data used for gene expression analysis (single individual *H*. *dujardini* and fast/slow dry of *R*. *varieornatus*) of the active state and tun state. After SAM to BAM conversion and sorting with SAMtools view and sort [98], we used QualiMap [99] and bedtools [100] for mapping quality check.

To annotate the predicted gene models, we performed similarity searches using BLAST BLASTP [67] against Swiss-Prot, TrEMBL [60], and HMMER hmmsearch [101] against Pfam-A [102] and Dfam [59], KAAS analysis for KEGG orthologue mapping [70], and InterProScan [103] for domain annotation. We used RepeatScout [104] and RepeatMasker [105] for de novo repeat identification. To compare *H*. *dujardini* gene models to those of *R*. *varieornatus*, we also ran BLAST BLASTP searches against the updated *R*. *varieornatus* proteome and TBLASTN search against the *R*. *varieornatus* genome and extracted bidirectional best hits with in-house Perl scripts.

For miRNA prediction, we used miRDeep [52] to predict mature miRNA within the genome, using the mature miRNA sequences in miRBase [53]. The predicted mature miRNA sequences were then searched against miRBase with ssearch36 [106] for annotation by retaining hits with identity >70% and a complete match of bases 1–7, 2–8, or 3–9.

### HGT analyses

HGT genes were identified using the HGT index approach [65]. Swiss-Prot and TrEMBL were downloaded [60], and sequences with “Complete Proteome” in the Keyword were extracted. Following the method of Boschetti et al., an Arthropoda-less and Nematoda-less database was constructed. These databases were searched with DIAMOND [66] using as query all CDS sequences, using the longest transcript for each gene (DIAMOND BLASTX). Hits with an E-value below 1e-5 were kept. The HGT index (Hu) was calculated as Bo − Bm, the bit score difference between the best nonmetazoan hit (Bo) and the best metazoan hit (Bm), and genes with Hu ≥ 30 were identified as HGT candidates.

To assess if ab initio annotation of genomes biases the calculation of the HGT index, we calculated HGT indices for genomes in ENSEMBL-Metazoa [57] that had corresponding Augustus [64] gene models and ran ab initio gene prediction. We analyzed *Aedes aegypti*, *Apis mellifera*, *Bombus impatiens*, *Caenorhabditis brenneri*, *Caenorhabditis briggsae*, *C*. *elegans*, *Caenorhabditis japonica*, *Caenorhabditis remanei*, *Culex quinquefasciatus*, *Drosophila ananassae*, *Drosophila erecta*, *Drosophila grimshawi*, *D*. *melanogaster*, *Drosophila mojavensis*, *Drosophila persimilis*, *Drosophila pseudoobscura*, *Drosophila sechellia*, *Drosophila simulans*, *Drosophila virilis*, *Drosophila willistoni*, *Drosophila yakuba*, *Heliconius melpomene*, *Nasonia vitripennis*, *Rhodnius prolixus*, *Tribolium castaneum*, and *Trichinella spiralis*. Gene predictions for each organism were conducted using autoAugPred.pl from the Augustus package with the corresponding model (S8 Table). The longest isoform sequences for all genes were extracted for both ENSEMBL and ab initio annotations, and the HGT index was calculated for each gene in all organisms. To assess if using DIAMOND BLASTX biases HGT index calculation, we ran BLAST BLASTX [67] searches with *H*. *dujardini* and calculated the HGT index using the same pipeline.

The blast-score-based HGT index provided a first-pass estimate of whether a gene had been horizontally transferred from a nonmetazoan species. Phylogenetic trees were constructed for each of the 463 candidates (based on the HGT index) along with their best blast hits as described above (S5 Data). Protein sequences for the blast hits were aligned with the HGT candidate using MAFFT [107]. RAxML [69] was used to build 450 individual trees, as 13 of the protein sets had less than 4 sequences and trees could not be built for them. HGT candidates were categorized as prokaryotes, viruses, metazoan, and nonmetazoan (i.e., eukaryotes that were nonmetazoan, such as fungi) based on the monophyletic clades that they were placed in. Any that could not be classified monophyletically were classified as “complex”: these were split into complex non-HGT (where the complexity was within a metazoan radiation) or complex HGT (where HGT was affirmed but affinities remained unclear) (S4 Data). OrthoFinder [62] with default BLAST+ BLASTP search settings and an inflation parameter of 1.5 was used to identify orthogroups containing *H*. *dujardini* and *R*. *varieornatus* protein-coding genes. These orthogroups were used to identify the *R*. *varieornatus* HGT homologues of *H*. *dujardini* HGT candidates. HGT candidates were classified as having high gene expression levels if they had an average gene expression greater than the overall average gene expression level of 1 TPM.

### Anhydrobiosis analyses

To identify genes responsive to anhydrobiosis, we explored transcriptome (Illumina mRNA-Seq) data for both *H*. *dujardini* and *R*. *varieornatus*. Individual mRNA-Seq data for *H*. *dujardini* [42] before and during anhydrobiosis were contrasted with new sequence data for *R*. *varieornatus* similarly treated. We mapped the mRNA-Seq reads to the coding sequences of the relevant species with BWA MEM [97], and after summarizing the read count of each gene, we used DESeq2 [108] for differential expression calculation, using false discovery rate (FDR) correction. Genes with a FDR below 0.05, an average expression level (in transcripts per kilobase of model per million mapped fragments; TPM) of over 1, and a fold change over 2 were defined as differentially expressed genes. Gene expression (TPM) was calculated with Kallisto [109] and was parsed with custom Perl scripts. To assess if there were any differences in fold change distributions, we used R to calculate the fold change for each gene (anhydrobiotic / [active + 0.1]) and conducted a U test using the wilcox.text() function. We mapped the differentially expressed genes to KEGG pathway maps [110] to identify pathways that were likely to be differentially active during anhydrobiosis.

### Protein family analyses and comparative genomics

For comparison with *R*. *varieornatus*, we first aligned the genomes of *H*. *dujardini* and *R*. *varieornatus* with Murasaki and visualized with gmv [61]. The lower tf-idf anchor filter was set to 500. A syntenic block was observed between scaffold0001 of *H*. *dujardini* and scaffold002 of *R*. *varieornatus*. We extracted the corresponding regions (*H*. *dujardini*: scaffold0001 363,334–2,100,664, *R*. *varieornatus*: scaffold002 2,186,607–3,858,816), and conducted alignment with Mauve [111]. We determined the number of bidirectional best hit (BBH) orthologues on the same scaffold in both *H*. *dujardini* and *R*. *varieornatus*. We extracted gene pairs that had an identity of more than 90% by ClustalW2 [103] and calculated the identity of the first and last exon between pairs. Tardigrade-specific, protection-related genes (CAHS, SAHS, MAHS, RvLEAM, and Dsup) were identified by BLASTP, subjected to phylogenetic analysis using Clustalw2 [103] and FastTree [112], and visualized with FigTree [113].

HOX loci were identified using BLAST, and their positions on scaffolds and contigs assessed. To identify HOX loci in other genomes, genome assembly files were downloaded from ENSEMBL Genomes [57] or Wormbase ParaSite [114,115] and formatted for local search with BLAST+ [68]. Homeodomain alignments were generated using Clustal Omega [116], and phylogenies estimated with RAxML [69] to classify individual homeodomains.

Protein predictions from genomes of Annelida (1 species), Nematoda (9), Arthropoda (15), Mollusca (1), and Priapulida (1) were retrieved from public databases (S7 Table). Proteomes were screened for isoforms (S12 Data), and the longest isoforms were clustered with the proteins of *H*. *dujardini* and *R*. *varieornatus* using OrthoFinder 1.1.2 [62] at different inflation values (S10 Data). Proteins from all proteomes were functionally annotated using InterProScan [103]. OrthoFinder output was analyzed using KinFin [63] under 2 competing phylogenetic hypotheses: either (1) “Panarthropoda,” where Tardigrada and Arthropoda share a more recent common ancestor distinct from Nematoda, or (2) Tardigrada and Nematoda sharing a more recent common ancestor distinct from Arthropoda. (see S11 Data for input files used in KinFin analysis). Enrichment and depletion in clusters containing proteins from Tardigrada and other taxa was tested using a 2-sided Mann-Whitney-U test of the count (if at least 2 taxa had non-zero counts), and results were deemed significant at a *p*-value threshold of *p* = 0.01.

A graph representation of the OrthoFinder clustering (at inflation value = 1.5) was generated using the generate_network.py script distributed with KinFin. The nodes in the graph were positioned using the ForceAtlas2 layout algorithm implemented in Gephi.

Single-copy orthologues between *H*. *dujardini* and *R*. *varieornatus* were identified using the orthologous groups defined by OrthoFinder. Using the Ensembl Perl API, gene structure information (gene lengths, exon counts, and intron spans per gene) were extracted for these gene pairs. To avoid erroneous gene predictions biasing observed trends, *H*. *dujardini* genes that were 20% longer or 20% shorter were considered outliers.

### Phylogenomics

The whole-genome OrthoFinder clustering at inflation value 1.5 was mined for potential single-copy orthologues for phylogenetic analysis. Transcriptome data were retrieved for additional tardigrades (2 species), a priapulid (1), kinorhynchs (2), and onychophorans (3) (S11 Table). Assembled transcripts for *E*. *testudo*, *M*. *tardigradum*, *Pycnophyes kielensis*, and *Halicryptus spinulosus* were downloaded from the NCBI Transcriptome Shotgun Assembly (TSA) Database. EST sequences of *Euperipatoides kanangrensis*, *Peripatopsis sedgwicki*, and *Echinoderes horni* were download from NCBI Trace Archive and assembled using CAP3 [117]. Raw mRNA-Seq reads for *Peripatopsis capensis* were downloaded from NCBI SRA, trimmed using skewer [118], and assembled with Trinity [56]. Protein sequences were predicted from all transcriptome data using TransDecoder [119], retaining a single open reading frame per transcript. Predicted proteins from these transcriptomes were used along with the genome-derived proteomes in a second OrthoFinder clustering analysis.

We identified putatively orthologous genes in the OrthoFinder clusters for the genome and the genome-plus-transcriptome datasets. For both datasets, the same pipeline was followed. Clusters with 1-to-1 orthology were retained. For clusters with median per-species membership equal to 1 and mean less than 2.5, a phylogenetic tree was inferred with RAxML (using the LG+G model). Each tree was visually inspected to identify the largest possible monophyletic clan, and in-paralogues and spuriously included sequences were removed. Individual alignments of each locus were filtered using trimal [120] and then concatenated into a supermatrix using fastconcat [121]. The supermatrices were analyzed with RAxML [69] with 100 ML bootstraps and PhyloBayes [122] (see S11 Table for specific commands). Trees were summarized in FigTree.

### Databasing

A dedicated Ensembl genome browser (version 85) [57] using the EasyImport pipeline [123] was constructed on http://www.tardigrades.org, and the *H*. *dujardini* genome and annotations described in this paper and the new gene predictions for *R*. *varieornatus* were imported.

### Software usage and data manipulation

We used open source software tools where available, as detailed in S11 Table. Custom scripts developed for the project are uploaded to https://github.com/abs-yy/Hypsibius_dujardini_manuscript. We used G-language Genome Analysis Environment [124, 125] for sequence manipulation.

## Supporting information

S1 Fig DNA sequencing coverage of the *H*. *dujardini* genome. (DOCX)

S2 Fig tRNA genes in *Hypsibius dujardini* and *Ramazzottius varieornatus*. (DOCX)

S3 Fig Comparisons of genic features between *Hypsibius dujardini* and *Ramazzottius varieornatus*. (DOCX)

S4 Fig Clustered HGT loci in *H*. *dujardini* and *R*. *varieornatus*. (DOCX)

S5 Fig Phylogeny of protection-related proteins. (DOCX)

S6 Fig Biochemical pathways acquired or supplemented by HGT in *H*. *dujardini* and *R*. *varieornatus*. (DOCX)

S1 Table Data used in this study. (DOCX)

S2 Table Mapping statistics of DNA sequencing data. (DOCX)

S3 Table Repeat content of the genomes of *Hypsibius dujardini* and *Ramazzottius varieornatus*. (DOCX)

S4 Table Low-complexity and possible telomeric repeats in the *H*. *dujardini* genome. (DOCX)

S5 Table Mapping proportions of RNA-Seq data. (DOCX)

S6 Table Mapping proportion of the *Hypsibius dujardini* Trinity assembled transcriptome. (DOCX)

S7 Table Data used for clustering and phylogenomics. (DOCX)

S8 Table HGT content calculation of Ecdysozoa. (DOCX)

S9 Table Tardigrade-specific, protection-related proteins. (DOCX)

S10 Table Synapomorphies identified under different systematic hypotheses. (DOCX)

S11 Table Software used in this study. (DOCX)

S1 Data *Hypsibius dujardini* miRNA data. (1) Provisional id: identifier for miRNA sequence, based on scaffold of origin. (2) miRDeep2 score: score for miRNA as given by miRGeep2. (3) Total read count: total number of reads mapping to this miRNA model. (4) Mature read count: number of reads mapping to majority strand of predicted hairpin model. (5) Loop read count: number of reads mapping to predicted loop region of model. (6) Star read count: number of reads mapping to minority strand of predicted hairpin model. (7) Significant randfold *p*-value. (8) Example miRBase miRNA with the same seed: an example from miRBase of this miRNA family. (9) Consensus mature sequence. (10) Consensus star sequence. (11) Consensus precursor sequence. (12) Precursor coordinate: coordinates of the predicted miRNA on the scaffold.
(XLSX)

S2 Data Orthology analyses: Analysis of overrepresentation of protein families in tardigrades. *Supplementary comments on protein families with four-fold overrepresentation in tardigrades*. Family OG0000104 had 284 members, 276 of which derived from tardigrades, and was annotated as having a receptor ligand binding domain, but was not otherwise distinguished. OG004022 had 5 members in *H*. *dujardini *and 9 members in *R*. *variornatus*, but a mode of 1 (and maximum of 4 in *Octopus bimaculatus*) in other species. It is a member of a deeply conserved, otherwise uncharacterized, transmembrane protein family of unknown function. OG0001636 gathers a deeply conserved ATP-binding cassette family, and *while the 27 other species had a mode of 1 (and a maximum of 2)*, *R*. *varieornatus had 4 and H*. *dujardini* had 9 copies. OG0002927 encodes protein kinases, present in 23 of the 29 species, with 6 in *H*. *dujardini*, 5 in *R*. *varieornatus* and a mode of 1 elsewhere. OG0004228 is annotated as a relish-like transcription factor, and has 1 copy in the non-tardigrade species (except for two insects with 2) and 5 copies in each tardigrade. OG0001359, with 1 copy in most species, 8 in *H*. *dujardini*, 8 in *R*. *varieornatus*, and 4 in *Solenopsis invictus*, is likely to be a SAM-dependent methyltransferase (type 11), possibly involved in coenzyme biosynthesis. OG001949 had 1 copy in most species but 6 in *H*. *dujardini* and 4 in *R*. *varieornatus*, and is annotated as a RAB GTP hydrolase. OG0003870 was unannotated (containing only matches to domain of unknown function DUF1151), and elevated in *R*. *varieornatus* (9 copies) compared to other species (mode of 1; *H*. *dujardini* had 2). The three clusters with depletion in the tardigrades were OG0000604, encoding an exoribonuclease (1 copy in each tardigrade, but an average of three copies in the other 27 species), OG0000950, a 3'5'-cyclic nucleotide phosphodiesterase (1 in tardigrades versus 2.3 elsewhere) and OG00001138, an EF-hand protein (1 in tardigrades versus 2 elsewhere). In the Excel-format file the columns are as follows: (1) #cluster_id: protein family identifier; (2) cluster_status: “present” indicates present in either or both tardigrades; (3) cluster_type: “shared” indicates present in both tardigrades; (4) cluster_protein_count: the number of proteins in the cluster; (5) cluster_proteome_count: the number of proteomes contributing a member to the cluster; (6) Tardigrade_protein_count: the number of members that come from the tardigrade species; (7) Tardigrade_mean_count: the mean number of members per tartdigrade; (8) non_Tardigrade_mean_count: the mean number of members across the other species; (9) representation: the direction of the difference found; “enriched” = enriched in tardigrades; “depleted” = depleted in tardigrades; (10) log2_mean(Tardigrade/others): the ratio of mean per-species membership in tardigrades to others; (11) mwu_pvalue(Tardigrade vs. others): Mann-Whitney U-test for difference in representation in the tardigrades; (12) Tardigrade_proteome_coverage: the proportion of tardigrade proteomes that contributed to the cluster; (13) Tardigrade_proteomes_present_count: the number of tardigrade proteomes in the clustering analyses; (14) Tardigrade_proteomes_present: which tardigrade proteomes contributed members to the cluster; (15) GO: GO terms associated with the cluster (based on InterPro matches; given as GO identifier:number of members with that term); (16) IPR: InterPro domains associated with the cluster (given as IPR identifier:number of members with that domain); (17) Pfam: Pfam domains associated with the cluster (given as Pfam identifier:number of members with that domain); (18) SignalP_EUK: Presence of eukaryotic signal peptides in members of the cluster (given as number of members with each pattern of signal peptide (SP) and transmembrane (TM) domains).
(XLSX)

S3 Data List of tardigrade specific cluster domains. (XLSX)

S4 Data 463 putative HGTs in *Hypsibius dujardini*. This excel file lists the properties of the 463 loci identified as potential HGTs in *H*. *dujardini *using the HGT index approach. The columns in the table are as follows: (1) protein: name of the *H*. *dujardini* HGT candidate; (2) best_metazoan_hit: identifier for the best hit in Metazoa (as identifier|database|classification); (3) metazoan_hitscore: bit score for the best metazoan hit; (4) best_nonmetazoan_hit: identifier for the best hit in non-metazoan organisms (as identifier|database|classification); (5) nonmetazoan_hitscore: bit score for the best non-metazoan hit; (6) hits_to_metazoan_nonmetazoan: summary of whether the protein has hits to one or both of Metazoa and non-Metazoa; (7) score_difference: difference in BLAST bitscore between metazoan and non-metazoan hits, the HGT index; (8) HGT_index_gt30: is the difference between best Metazoan and best non-metazoan hit scores greater than 30—the HGT index score; (9) has_metazoan_neighbour_on_same_scaffold: Does the locus have neighbours on the same scaffold with uncomplicated metazoan similarities? (10) intron_count: number of introns predicted in the gene; (11) expr_level_in_any_lib_gt_1tpm: Does the gene have evidence of expression from RNA-Seq data? hiexp = greater than 1 TPM, lowexp = less than 1 TPM; (12) tree_classification: the taxonomic classification of the locus based on RAXML phylogenetic analyses; (13) OrthogroupName: the OrthoFinder orthogroup the locus is a member of; (14) High_likelihood_HGTs: classification of likelihood of being a true HGT, with evidence supporting HighLikelihood calls; (15) Ramazzottius_HGT_homologues: orthologues and homiologues in *R*. *varieornatus *(names are *R*. *varieornatus* gene names); (16) Short_InterproScan_Names: summary of InterProScan-derived domain annotations for the locus. Where more than one domain is found, the different domains are separated by “|” characters.
(XLSX)

S5 Data Phylogenetic analyses of potential horizontal gene transfer candidates. These data are the phylogenies from which putative HGTs were classified by taxon-of-origin. This compressed file (tar-gzipped) contains a directory, that holds five subdirectories, “complex”, “metazoan”, “non-metazoan”, “prokaryotes”, “viruses”. In each subdirectory is the set of text files containing NEWICK format trees for putative HGT loci generated by RAXML search, as described in the Methods. Each tree has a name of the form “RAxML_bestTree.bHd00409.1.faa.aln.ml.newick”: RAXML best tree for gene bHd00409.1, amino acid fasta alignment, generated with maximum likelihood, NEWICK format.
(GZ) Click here for additional data file.

S6 Data Physical clustering of horizontal gene transfer candidates in the genome. This table summarises the gene neighbourhood analysis of *H*. *dujardini *HGT<br class = "kix-line-break">candidates. Highlighted rows indicate putative HGT gene sets that are very close on the *H*. *dujardini* genome. For this analysis, the scaffolds were concatenated into one linear sequence, and thus one step in the assessment was determining the scaffold origin of loci (columns 13, 14). The columns are as follows: (1) Gene: putative HGT candidate gene name; (2) Count: index count; (3) Scaffold: scaffold from which the gene is predicted; (4) Number: number of gene on scaffold; (5) PrevNum: number of the closest HGT candidate to the left of the gene (set to -1000000 if the gene is the first on the scaffold); (6) PrevGene: putative downstream HGT candidate gene name; (7) Number: number of gene on scaffold; (8) NextNum: number of the closest HGT candidate to the right of the gene (set to 1000000 if the gene is the last on the scaffold); (9) NextGene: putative upstream HGT candidate gene name; (10) DifPrev: number of genes between focal gene and next HGT candidate to left; (11) DifNext: number of genes between focal gene and next HGT candidate to right; (12) DiffClose: number of genes between focal gene and next closest HGT candidate (minimum of columns 10 and 11); (13) ScaffClose: the scaffold on which the closest next HGT gene resides; (14) SameScaf: whether this scaffold is the same as the scaffold on which the focal gene resides; (15) DiffClose: number of genes between focal gene and next closest HGT candidate on the same scaffold (minimum of columns 10 and 11); (16) Phylo: phylogenetic classification of the taxon-of-origin of the focal locus; (17) TrEMBL: best BLAST match of the focal locus in TrEMBL; (18) Swiss-Prot: best BLAST match of the focal locus in SwissProt.
(XLSX)

S7 Data Genes differentially expressed in anhydrobiosis. This table describes the analysis of differential gene expression under anhydrobiosis of *H*. *dujardini*, based on mRNA-Seq of active and anhydrobiotic, “tun” animals. TPM: transcripts per million mapped. The columns are as follows: (1) Gene: gene name; (2) Active-1: TPM in sample of active animals 1, assessed by Kallisto; (3) Active-2: TPM in sample of active animals 2, assessed by Kallisto; (4) Active-3: TPM in sample of active animals 3, assessed by Kallisto; (5) Tun-1: TPM in sample of tun animals 1, assessed by Kallisto; (6) Tun-2: TPM in sample of tun animals 2, assessed by Kallisto; (7) Tun-3: TPM in sample of tun animals 3, assessed by Kallisto; (8) FoldChange: fold change in tun animals (mean of tun TPM/mean of active TPM); (9) Active-1: fragments mapped in sample of active animals 1 using BWA; (10) Active-2: fragments mapped in sample of active animals 2 using BWA; (11) Active-3: fragments mapped in sample of active animals 3 using BWA; (12) Tun-1: fragments mapped in sample of tun animals 1 using BWA; (13) Tun-2: fragments mapped in sample of tun animals 2 using BWA; (14) Tun-3: fragments mapped in sample of tun animals 3 using BWA; (15) p-value: p-value of differential expression between tun and active animals estimated using DESeq; (16) FDR: false discovery rate estimate; (17) Database Search: BLAST matches for locus based on BLAST search of SwissProt/TrEMBL. Blank entries indicate no matches with E-values less than 1e-5.
(XLSX)

S8 Data Dayhoff recoded phylogenetic analyses in NEWICK format. (TXT)

S9 Data Functional annotation of genes differentially expressed in anhydrobiosis. Functional annotations of the *H*. *dujardini* genes found to be differentially expressed under anhydrobiosis, based on InterPro domain matches. The columns in the table are: (1) #cluster_id: cluster identifier summary; (2) cluster_id: cluster identifier (with one line for each domain matched); (3) domain_source: database source of domain model (IPR = InterProScan); (4) domain_id: database identifier for domain model; (5) domain_description: summary description for domain model; (6) protein_count: total number of proteins in the cluster; (7) protein_count_with_domain: number of proteins in the cluster annotated with this domain; (8) HDUJA-DEG: number of *H*. *dujardini* members of the cluster that are differentially expressed under anhydrobiosis; (9) HDUJA-DEG-DOMAINS: number of *H*. *dujardini* members of the cluster that are differentially expressed under anhydrobiosis that have this domain, and how many copies of the domain each has (e.g. 4x1 means four members have 1 copy each); (10) HDUJA-HGT: number of *H*. *dujardini* members of the cluster that are potential HGT candidates; (11) RVARI-DEG: number of *R*. *varieornatus* members of the cluster that are differentially expressed under anhydrobiosis; (12) RVARI-DEG-DOMAINS: number of *R*. *varieornatus *members of the cluster that are differentially expressed under anhydrobiosis that have this domain, and how many copies of the domain each has (e.g. 4x1 means four members have 1 copy each); (13) proteomes_with_domain_fraction: proportion of all proteomes represented in the cluster where a member has the domain; (14) proteomes_with_domain: proteomes with members in the cluster where that member has the domain.
(XLSX)

S10 Data Orthology analyses—Orthofinder clustering outputs. This file, a tarred and gzipped archive, contains the outputs from OrthoFinder analyses conducted under different inflation values.
(GZ) Click here for additional data file.

S11 Data Orthology analyses—KinFin input and output files. This tarred and gzipped archive contains the input files for KinFin and the output from the<br class = "kix-line-break">analyses. The six input files include two alternative tree hypotheses, a file giving the functional<br class = "kix-line-break">annotation of the proteins, a species classification file and files giving the links between species and sequences. The output files are given for each inflation value applied in OrthoFinder, and include analyses under both hypotheses of tardigrade relationships.
(GZ) Click here for additional data file.

S12 Data Input proteome FASTA files for orthology analyses. A tarred and gzipped archive of the protein fasta format files used in orthology analyses, including data from 29 species. In these files only the longest isoform has been retained from groups of sequences derived from the same locus.
(GZ) Click here for additional data file.

## Abbreviations

AMPK: 5' adenosine monophosphate-activated protein kinase
API: application-programming interface
ATM: ataxia-telangiectasia mutated
ATR: ataxia telangiectasia and Rad3-related protein
BER: base excision repair
BLAST: Basic Local Alignment Search Tool
BSC1: bypass of stop codon protein 1
BUSCO: Benchmarking Universal Single-Copy Orthologs
CAHS: cytosolic abundant heat soluble
CaM: calmodulin
CASP: caspase
CATE: catalase
CBF: C-repeat-binding factor
CDS: coding sequence
CEGMA: Core Eukaryotic Genes Mapping Approach
CHK: checkpoint kinase
CNG: cyclic nucleotide-gated channel
Dsup: damage suppressor
EGF: epidermal growth factor
GPCR: G-protein-coupled receptor
GST: glutathione S-transferase
GTP: guanosine-5'-triphosphate
GTR+G: General Time Reversible model with Gamma distribution of rates model
GTR-CAT+G: GTR plus rate categories model
HGT: horizontal gene transfer
HR: homologous recombination
HSP: heat shock protein
INSDC: International Nucleotide Sequence Database Collaboration
IPR: InterPro domain identifier
KAAS: KEGG Automatic Annotation Server
KEGG: Kyoto Encyclopedia of Genes and Genomes
LDL: low-density lipoprotein
MAHS: mitochondrial abundant heat soluble protein
MCL: Markov Cluster Algorithm
miRNA-Seq: microRNA sequencing
MMR: mismatch repair
mTOR: mechanistic target of rapamycin
NER: nucleotide excision repair
NHEJ: nonhomologous end joining
PacBio: Pacific Biosciences
PARP: poly(ADP-ribose) polymerase
PF: PFam identifier
PHD: plant homeodomain
RAxML: Randomized Axelerated Maximum Likelihood
RH: relative humidity
RNAi: RNA interference
RNA-Seq: RNA sequencing
ROS: reactive oxygen species
RyLEAM: late embryogenesis abundant protein mitochondrial
SAHS: secretory abundant heat soluble
SIRT1: sirtuin 1
SOD: superoxide dismutase
SRA: Sequence Read Archive
TPM: transcript(s) per million
TPS: trehalose-6-phosphatase synthase
TSC1/2: tuberous sclerosis 1/2
UDP: uridine diphosphate
UGT: UDP-glucuronosyltransferase
VHL: von Hippel-Lindau tumor suppressor

## References

[1] Dunn CW, Hejnol A, Matus DQ, Pang K, Browne WE, Smith SA, et al Broad phylogenomic sampling improves resolution of the animal tree of life. Nature. 2008;452(7188):745–9. doi: 10.1038/nature06614 . 18322464 10.1038/nature06614

[2] Campbell LI, Rota-Stabelli O, Edgecombe GD, Marchioro T, Longhorn SJ, Telford MJ, et al MicroRNAs and phylogenomics resolve the relationships of Tardigrada and suggest that velvet worms are the sister group of Arthropoda. Proc Natl Acad Sci U S A. 2011;108(38):15920–4. doi: 10.1073/pnas.1105499108 ; 21896763 10.1073/pnas.1105499108 PMC3179045

[3] Borner J, Rehm P, Schill RO, Ebersberger I, Burmester T. A transcriptome approach to ecdysozoan phylogeny. Mol Phylogenet Evol. 2014;80:79–87. doi: 10.1016/j.ympev.2014.08.001 . 25124096 10.1016/j.ympev.2014.08.001

[4] Edgecombe GD. Arthropod phylogeny: an overview from the perspectives of morphology, molecular data and the fossil record. Arthropod Struct Dev. 2010;39(2–3):74–87. doi: 10.1016/j.asd.2009.10.002 . 19854297 10.1016/j.asd.2009.10.002

[5] Nielsen C. The triradiate sucking pharynx in animal phylogeny. Invertebrate Biology. 2013;132(1):1–13. doi: 10.1111/ivb.12010

[6] Degma P, Bertolani R, Guidetti R. Actual checklist of Tardigrada species 2016 [cited 2017 FEB 16]. 2016/12/15:[1–47]. http://www.tardigrada.modena.unimo.it./miscellanea/Actual checklist of Tardigrada.pdf.

[7] Clegg JS. Cryptobiosis—a peculiar state of biological organization. Comp Biochem Physiol B Biochem Mol Biol. 2001;128(4):613–24. . 11290443 10.1016/s1096-4959(01)00300-1

[8] Horikawa DD, Cumbers J, Sakakibara I, Rogoff D, Leuko S, Harnoto R, et al Analysis of DNA repair and protection in the Tardigrade Ramazzottius varieornatus and Hypsibius dujardini after exposure to UVC radiation. PLoS ONE. 2013;8(6):e64793 doi: 10.1371/journal.pone.0064793 ; 23762256 10.1371/journal.pone.0064793 PMC3675078

[9] Altiero T, Guidetti R, Caselli V, Cesari M, Rebecchi L. Ultraviolet radiation tolerance in hydrated and desiccated eutardigrades. J Zool Syst Evol Res. 2011;49:104–10. doi: 10.1111/j.1439-0469.2010.00607.x

[10] Hengherr S, Worland MR, Reuner A, Brummer F, Schill RO. Freeze tolerance, supercooling points and ice formation: comparative studies on the subzero temperature survival of limno-terrestrial tardigrades. J Exp Biol. 2009;212(Pt 6):802–7. doi: 10.1242/jeb.025973 . 19251996 10.1242/jeb.025973

[11] Jonsson KI, Harms-Ringdahl M, Torudd J. Radiation tolerance in the eutardigrade Richtersius coronifer. Int J Radiat Biol. 2005;81(9):649–56. doi: 10.1080/09553000500368453 . 16368643 10.1080/09553000500368453

[12] May RM, Maria M, Gumard J. Action différentielle des rayons x et ultraviolets sur le tardigrade Macrobiotus areolatus, a l’état actif et desséché. Bull Biol Fr Belg. 1964;98:349–36719.

[13] Persson D, Halberg KA, Jorgensen A, Ricci C, Mobjerg N, Kristensen RM. Extreme stress tolerance in tardigrades: surviving space conditions in low earth orbit. J Zool Syst Evol Res. 2011;49:90–7. doi: 10.1111/j.1439-0469.2010.00605.x

[14] Ramløv H, Westh P. Cryptobiosis in the Eutardigrade Adorybiotus (Richtersius) coronifer: Tolerance to Alcohols, Temperature and de novo Protein Synthesis. Zool Anz. 2001;240(3–4):517–23. doi: 10.1078/0044-5231-00062

[15] Ono F, Mori Y, Takarabe K, Fujii A, Saigusa M, Matsushima Y, et al Effect of ultra-high pressure on small animals, tardigrades and Artemia. Cogent Physics. 2016;3(1):1167575 doi: 10.1080/23311940.2016.1167575

[16] Beltrán-Pardo EA, Jönsson I, Wojcik A, Haghdoost S, Bermúdez Cruz RM, Bernal Villegas JE. Sequence analysis of the DNA-repair gene rad51 in the tardigrades Milnesium cf. tardigradum, Hypsibius dujardini and Macrobiotus cf. harmsworthi. J Limnol. 2013;72(s1):80–91. doi: 10.4081/jlimnol.2013.s1.e10

[17] Mali B, Grohme MA, Forster F, Dandekar T, Schnolzer M, Reuter D, et al Transcriptome survey of the anhydrobiotic tardigrade Milnesium tardigradum in comparison with Hypsibius dujardini and Richtersius coronifer. BMC Genomics. 2010;11(168):168 doi: 10.1186/1471-2164-11-168 ; 20226016 10.1186/1471-2164-11-168 PMC2848246

[18] Gusev O, Suetsugu Y, Cornette R, Kawashima T, Logacheva MD, Kondrashov AS, et al Comparative genome sequencing reveals genomic signature of extreme desiccation tolerance in the anhydrobiotic midge. Nat Commun. 2014;5(4784):1–9. doi: 10.1038/ncomms5784 ; 25216354 10.1038/ncomms5784 PMC4175575

[19] Boothby TC, Tenlen JR, Smith FW, Wang JR, Patanella KA, Nishimura EO, et al Evidence for extensive horizontal gene transfer from the draft genome of a tardigrade. Proc Natl Acad Sci U S A. 2015;112(52):15976–81. doi: 10.1073/pnas.1510461112 ; 26598659 10.1073/pnas.1510461112 PMC4702960

[20] Koutsovoulos G, Kumar S, Laetsch DR, Stevens L, Daub J, Conlon C, et al No evidence for extensive horizontal gene transfer in the genome of the tardigrade Hypsibius dujardini. Proc Natl Acad Sci U S A. 2016;113(18):5053–8. doi: 10.1073/pnas.1600338113 ; 27035985 10.1073/pnas.1600338113 PMC4983863

[21] Arakawa K. No evidence for extensive horizontal gene transfer from the draft genome of a tardigrade. Proc Natl Acad Sci U S A. 2016;113(22):E3057 doi: 10.1073/pnas.1602711113 ; 27173901 10.1073/pnas.1602711113 PMC4896722

[22] Hashimoto T, Horikawa DD, Saito Y, Kuwahara H, Kozuka-Hata H, Shin IT, et al Extremotolerant tardigrade genome and improved radiotolerance of human cultured cells by tardigrade-unique protein. Nat Commun. 2016;7:12808 doi: 10.1038/ncomms12808 ; 27649274 10.1038/ncomms12808 PMC5034306

[23] Kondo K, Kubo T, Kunieda T. Suggested Involvement of PP1/PP2A Activity and De Novo Gene Expression in Anhydrobiotic Survival in a Tardigrade, Hypsibius dujardini, by Chemical Genetic Approach. PLoS ONE. 2015;10(12):e0144803 doi: 10.1371/journal.pone.0144803 ; 26690982 10.1371/journal.pone.0144803 PMC4686906

[24] Horikawa DD, Kunieda T, Abe W, Watanabe M, Nakahara Y, Yukuhiro F, et al Establishment of a rearing system of the extremotolerant tardigrade Ramazzottius varieornatus: a new model animal for astrobiology. Astrobiology. 2008;8(3):549–56. doi: 10.1089/ast.2007.0139 . 18554084 10.1089/ast.2007.0139

[25] Tenlen JR, McCaskill S, Goldstein B. RNA interference can be used to disrupt gene function in tardigrades. Dev Genes Evol. 2013;223(3):171–81. doi: 10.1007/s00427-012-0432-6 ; 23187800 10.1007/s00427-012-0432-6 PMC3600081

[26] Horikawa D. The tardigrade Ramazzottius varieornatus as a model of extremotolerant animals. J Jpn Soc Extremophiles. 2008;7.2(1):25–8. doi: 10.3118/jjse.7.2.25

[27] Rizzo AM, Negroni M, Altiero T, Montorfano G, Corsetto P, Berselli P, et al Antioxidant defences in hydrated and desiccated states of the tardigrade Paramacrobiotus richtersi. Comp Biochem Physiol B Biochem Mol Biol. 2010;156(2):115–21. doi: 10.1016/j.cbpb.2010.02.009 . 20206711 10.1016/j.cbpb.2010.02.009

[28] Jonsson KI, Schill RO. Induction of Hsp70 by desiccation, ionising radiation and heat-shock in the eutardigrade Richtersius coronifer. Comp Biochem Physiol B Biochem Mol Biol. 2007;146(4):456–60. doi: 10.1016/j.cbpb.2006.10.111 . 17261378 10.1016/j.cbpb.2006.10.111

[29] Reuner A, Hengherr S, Mali B, Forster F, Arndt D, Reinhardt R, et al Stress response in tardigrades: differential gene expression of molecular chaperones. Cell Stress Chaperones. 2010;15(4):423–30. doi: 10.1007/s12192-009-0158-1 ; 19943197 10.1007/s12192-009-0158-1 PMC3082643

[30] Schill RO, Steinbruck GH, Kohler HR. Stress gene (hsp70) sequences and quantitative expression in Milnesium tardigradum (Tardigrada) during active and cryptobiotic stages. J Exp Biol. 2004;207(Pt 10):1607–13. . 15073193 10.1242/jeb.00935

[31] Hengherr S, Heyer AG, Kohler HR, Schill RO. Trehalose and anhydrobiosis in tardigrades—evidence for divergence in responses to dehydration. FEBS J. 2008;275(2):281–8. doi: 10.1111/j.1742-4658.2007.06198.x . 18070104 10.1111/j.1742-4658.2007.06198.x

[32] Moore DS, Hansen R, Hand SC. Liposomes with diverse compositions are protected during desiccation by LEA proteins from Artemia franciscana and trehalose. Biochim Biophys Acta. 2016;1858(1):104–15. doi: 10.1016/j.bbamem.2015.10.019 . 26518519 10.1016/j.bbamem.2015.10.019

[33] Yamaguchi A, Tanaka S, Yamaguchi S, Kuwahara H, Takamura C, Imajoh-Ohmi S, et al Two novel heat-soluble protein families abundantly expressed in an anhydrobiotic tardigrade. PLoS ONE. 2012;7(8):e44209 doi: 10.1371/journal.pone.0044209 ; 22937162 10.1371/journal.pone.0044209 PMC3429414

[34] Tanaka S, Tanaka J, Miwa Y, Horikawa DD, Katayama T, Arakawa K, et al Novel mitochondria-targeted heat-soluble proteins identified in the anhydrobiotic Tardigrade improve osmotic tolerance of human cells. PLoS ONE. 2015;10(2):e0118272 doi: 10.1371/journal.pone.0118272 ; 25675104 10.1371/journal.pone.0118272 PMC4326354

[35] Kikawada T, Saito A, Kanamori Y, Nakahara Y, Iwata K, Tanaka D, et al Trehalose transporter 1, a facilitated and high-capacity trehalose transporter, allows exogenous trehalose uptake into cells. Proc Natl Acad Sci U S A. 2007;104(28):11585–90. doi: 10.1073/pnas.0702538104 ; 17606922 10.1073/pnas.0702538104 PMC1905927

[36] da Costa Morato Nery D, da Silva CG, Mariani D, Fernandes PN, Pereira MD, Panek AD, et al The role of trehalose and its transporter in protection against reactive oxygen species. Biochim Biophys Acta. 2008;1780(12):1408–11. doi: 10.1016/j.bbagen.2008.05.011 . 18601980 10.1016/j.bbagen.2008.05.011

[37] Madin KAC, Crowe JH. Anhydrobiosis in nematodes: Carbohydrate and lipid metabolism during dehydration. Journal of Experimental Zoology. 1975;193(3):335–42. doi: 10.1002/jez.1401930309

[38] Clegg JS. Metabolic studies of crytobiosis in encysted embryos of Artemia salina. Comparative Biochemistry and Physiology. 1967;20(3):801–9. doi: 10.1016/0010-406x(67)90054-0

[39] Bemm F, Weiss CL, Schultz J, Forster F. Genome of a tardigrade: Horizontal gene transfer or bacterial contamination? Proc Natl Acad Sci U S A. 2016;113(22):E3054–6. doi: 10.1073/pnas.1525116113 ; 27173902 10.1073/pnas.1525116113 PMC4896698

[40] Delmont TO, Eren AM. Identifying contamination with advanced visualization and analysis practices: metagenomic approaches for eukaryotic genome assemblies. PeerJ. 2016;4:e1839 doi: 10.7717/peerj.1839 ; 27069789 10.7717/peerj.1839 PMC4824900

[41] Boothby TC, Goldstein B. Reply to Bemm et al. and Arakawa: Identifying foreign genes in independent Hypsibius dujardini genome assemblies. Proc Natl Acad Sci U S A. 2016;113(22):E3058–61. doi: 10.1073/pnas.1601149113 ; 27173900 10.1073/pnas.1601149113 PMC4896697

[42] Arakawa K, Yoshida Y, Tomita M. Genome sequencing of a single tardigrade *Hypsibius dujardini* individual. Sci Data. 2016;3:160063 doi: 10.1038/sdata.2016.63 ; 27529330 10.1038/sdata.2016.63 PMC4986543

[43] Chin CS, Peluso P, Sedlazeck FJ, Nattestad M, Concepcion GT, Clum A, et al Phased diploid genome assembly with single-molecule real-time sequencing. Nat Methods. 2016;13(12):1050–4. doi: 10.1038/nmeth.4035 . 27749838 10.1038/nmeth.4035 PMC5503144

[44] Kajitani R, Toshimoto K, Noguchi H, Toyoda A, Ogura Y, Okuno M, et al Efficient de novo assembly of highly heterozygous genomes from whole-genome shotgun short reads. Genome Res. 2014;24(8):1384–95. doi: 10.1101/gr.170720.113 ; 24755901 10.1101/gr.170720.113 PMC4120091

[45] Gabriel WN, McNuff R, Patel SK, Gregory TR, Jeck WR, Jones CD, et al The tardigrade Hypsibius dujardini, a new model for studying the evolution of development. Dev Biol. 2007;312(2):545–59. doi: 10.1016/j.ydbio.2007.09.055 . 17996863 10.1016/j.ydbio.2007.09.055

[46] Kumar S, Jones M, Koutsovoulos G, Clarke M, Blaxter M. Blobology: exploring raw genome data for contaminants, symbionts and parasites using taxon-annotated GC-coverage plots. Front Genet. 2013;4:237 doi: 10.3389/fgene.2013.00237 ; 24348509 10.3389/fgene.2013.00237 PMC3843372

[47] Boetzer M, Pirovano W. SSPACE-LongRead: scaffolding bacterial draft genomes using long read sequence information. BMC Bioinformatics. 2014;15(211):211 doi: 10.1186/1471-2105-15-211 ; 24950923 10.1186/1471-2105-15-211 PMC4076250

[48] Parra G, Bradnam K, Korf I. CEGMA: a pipeline to accurately annotate core genes in eukaryotic genomes. Bioinformatics. 2007;23(9):1061–7. doi: 10.1093/bioinformatics/btm071 . 17332020 10.1093/bioinformatics/btm071

[49] Simao FA, Waterhouse RM, Ioannidis P, Kriventseva EV, Zdobnov EM. BUSCO: assessing genome assembly and annotation completeness with single-copy orthologs. Bioinformatics. 2015;31(19):3210–2. doi: 10.1093/bioinformatics/btv351 . 26059717 10.1093/bioinformatics/btv351

[50] Lagesen K, Hallin P, Rodland EA, Staerfeldt HH, Rognes T, Ussery DW. RNAmmer: consistent and rapid annotation of ribosomal RNA genes. Nucleic Acids Res. 2007;35(9):3100–8. doi: 10.1093/nar/gkm160 ; 17452365 10.1093/nar/gkm160 PMC1888812

[51] Lowe TM, Eddy SR. tRNAscan-SE: a program for improved detection of transfer RNA genes in genomic sequence. Nucleic Acids Res. 1997;25(5):955–64. doi: 10.1093/nar/25.5.0955 ; 9023104 10.1093/nar/25.5.955 PMC146525

[52] Friedlander MR, Mackowiak SD, Li N, Chen W, Rajewsky N. miRDeep2 accurately identifies known and hundreds of novel microRNA genes in seven animal clades. Nucleic Acids Res. 2012;40(1):37–52. doi: 10.1093/nar/gkr688 ; 21911355 10.1093/nar/gkr688 PMC3245920

[53] Kozomara A, Griffiths-Jones S. miRBase: annotating high confidence microRNAs using deep sequencing data. Nucleic Acids Res. 2014;42(Database issue):D68–73. doi: 10.1093/nar/gkt1181 ; 24275495 10.1093/nar/gkt1181 PMC3965103

[54] Hoff KJ, Lange S, Lomsadze A, Borodovsky M, Stanke M. BRAKER1: Unsupervised RNA-Seq-Based Genome Annotation with GeneMark-ET and AUGUSTUS. Bioinformatics. 2016;32(5):767–9. doi: 10.1093/bioinformatics/btv661 . 26559507 10.1093/bioinformatics/btv661 PMC6078167

[55] Holt C, Yandell M. MAKER2: an annotation pipeline and genome-database management tool for second-generation genome projects. BMC Bioinformatics. 2011;12:491 doi: 10.1186/1471-2105-12-491 ; 22192575 10.1186/1471-2105-12-491 PMC3280279

[56] Grabherr MG, Haas BJ, Yassour M, Levin JZ, Thompson DA, Amit I, et al Full-length transcriptome assembly from RNA-Seq data without a reference genome. Nat Biotechnol. 2011;29(7):644–52. doi: 10.1038/nbt.1883 ; 21572440 10.1038/nbt.1883 PMC3571712

[57] Aken BL, Achuthan P, Akanni W, Amode MR, Bernsdorff F, Bhai J, et al Ensembl 2017. Nucleic Acids Res. 2017;45(D1):D635–D42. doi: 10.1093/nar/gkw1104 ; 27899575 10.1093/nar/gkw1104 PMC5210575

[58] Yates A, Beal K, Keenan S, McLaren W, Pignatelli M, Ritchie GR, et al The Ensembl REST API: Ensembl Data for Any Language. Bioinformatics. 2015;31(1):143–5. doi: 10.1093/bioinformatics/btu613 ; 25236461 10.1093/bioinformatics/btu613 PMC4271150

[59] Hubley R, Finn RD, Clements J, Eddy SR, Jones TA, Bao W, et al The Dfam database of repetitive DNA families. Nucleic Acids Res. 2016;44(D1):D81–9. doi: 10.1093/nar/gkv1272 ; 26612867 10.1093/nar/gkv1272 PMC4702899

[60] UniProt C. UniProt: a hub for protein information. Nucleic Acids Res. 2015;43(Database issue):D204–12. doi: 10.1093/nar/gku989 ; 25348405 10.1093/nar/gku989 PMC4384041

[61] Popendorf K, Tsuyoshi H, Osana Y, Sakakibara Y. Murasaki: a fast, parallelizable algorithm to find anchors from multiple genomes. PLoS ONE. 2010;5(9):e12651 doi: 10.1371/journal.pone.0012651 ; 20885980 10.1371/journal.pone.0012651 PMC2945767

[62] Emms DM, Kelly S. OrthoFinder: solving fundamental biases in whole genome comparisons dramatically improves orthogroup inference accuracy. Genome Biol. 2015;16:157 doi: 10.1186/s13059-015-0721-2 ; 26243257 10.1186/s13059-015-0721-2 PMC4531804

[63] Laetsch DR. KinFin v0.8.1. 2017.

[64] Keller O, Kollmar M, Stanke M, Waack S. A novel hybrid gene prediction method employing protein multiple sequence alignments. Bioinformatics. 2011;27(6):757–63. doi: 10.1093/bioinformatics/btr010 . 21216780 10.1093/bioinformatics/btr010

[65] Boschetti C, Carr A, Crisp A, Eyres I, Wang-Koh Y, Lubzens E, et al Biochemical diversification through foreign gene expression in bdelloid rotifers. PLoS Genet. 2012;8(11):e1003035 doi: 10.1371/journal.pgen.1003035 ; 23166508 10.1371/journal.pgen.1003035 PMC3499245

[66] Buchfink B, Xie C, Huson DH. Fast and sensitive protein alignment using DIAMOND. Nature Methods. 2015;12(1):59–60. doi: 10.1038/nmeth.3176 25402007 10.1038/nmeth.3176

[67] Altschul SF, Madden TL, Schaffer AA, Zhang J, Zhang Z, Miller W, et al Gapped BLAST and PSI-BLAST: a new generation of protein database search programs. Nucleic Acids Res. 1997;25(17):3389–402. ; 9254694 10.1093/nar/25.17.3389 PMC146917

[68] Camacho C, Coulouris G, Avagyan V, Ma N, Papadopoulos J, Bealer K, et al BLAST+: architecture and applications. BMC Bioinformatics. 2009;10:421 doi: 10.1186/1471-2105-10-421 ; 20003500 10.1186/1471-2105-10-421 PMC2803857

[69] Stamatakis A. RAxML version 8: a tool for phylogenetic analysis and post-analysis of large phylogenies. Bioinformatics. 2014;30(9):1312–3. doi: 10.1093/bioinformatics/btu033 ; 24451623 10.1093/bioinformatics/btu033 PMC3998144

[70] Moriya Y, Itoh M, Okuda S, Yoshizawa AC, Kanehisa M. KAAS: an automatic genome annotation and pathway reconstruction server. Nucleic Acids Res. 2007;35(Web Server issue):W182–5. doi: 10.1093/nar/gkm321 ; 17526522 10.1093/nar/gkm321 PMC1933193

[71] Cornette R, Kikawada T. The induction of anhydrobiosis in the sleeping chironomid: current status of our knowledge. IUBMB Life. 2011;63(6):419–29. doi: 10.1002/iub.463 . 21547992 10.1002/iub.463

[72] Grohme MA, Mali B, Welnicz W, Michel S, Schill RO, Frohme M. The Aquaporin Channel Repertoire of the Tardigrade Milnesium tardigradum. Bioinform Biol Insights. 2013;7:153–65. doi: 10.4137/BBI.S11497 ; 23761966 10.4137/BBI.S11497 PMC3666991

[73] Oliveira RP, Porter Abate J, Dilks K, Landis J, Ashraf J, Murphy CT, et al Condition-adapted stress and longevity gene regulation by Caenorhabditis elegans SKN-1/Nrf. Aging Cell. 2009;8(5):524–41. doi: 10.1111/j.1474-9726.2009.00501.x ; 19575768 10.1111/j.1474-9726.2009.00501.x PMC2776707

[74] Rokas A, Holland PWH. Rare genomic changes as a tool for phylogenetics. Trends in Ecology & Evolution. 2000;15(11):454–9. doi: 10.1016/s0169-5347(00)01967-4 11050348 10.1016/s0169-5347(00)01967-4

[75] de Rosa R, Grenier JK, Andreeva T, Cook CE, Adoutte A, Akam M, et al Hox genes in brachiopods and priapulids and protostome evolution. Nature. 1999;399(6738):772–6. doi: 10.1038/21631 . 10391241 10.1038/21631

[76] Grenier JK, Garber TL, Warren R, Whitington PM, Carroll S. Evolution of the entire arthropod Hox gene set predated the origin and radiation of the onychophoran/arthropod clade. Current Biology. 1997;7(8):547–53. doi: 10.1016/s0960-9822(06)00253-3 9259556 10.1016/s0960-9822(06)00253-3

[77] Janssen R, Eriksson BJ, Tait NN, Budd GE. Onychophoran Hox genes and the evolution of arthropod Hox gene expression. Front Zool. 2014;11(1):22 doi: 10.1186/1742-9994-11-22 ; 24594097 10.1186/1742-9994-11-22 PMC4015684

[78] Smith FW, Boothby TC, Giovannini I, Rebecchi L, Jockusch EL, Goldstein B. The Compact Body Plan of Tardigrades Evolved by the Loss of a Large Body Region. Curr Biol. 2016;26(2):224–9. doi: 10.1016/j.cub.2015.11.059 . 26776737 10.1016/j.cub.2015.11.059

[79] Aboobaker A, Blaxter M. Hox gene evolution in nematodes: novelty conserved. Current Opinion in Genetics & Development. 2003;13(6):593–8. doi: 10.1016/j.gde.2003.10.009 14638320 10.1016/j.gde.2003.10.009

[80] Aboobaker AA, Blaxter ML. Hox Gene Loss during Dynamic Evolution of the Nematode Cluster. Current Biology. 2003;13(1):37–40. doi: 10.1016/s0960-9822(02)01399-4 12526742 10.1016/s0960-9822(02)01399-4

[81] Aboobaker A, Blaxter M. The Nematode Story: Hox Gene Loss and Rapid Evolution In: Deutsch JS, editor. Hox Genes: Studies from the 20th to the 21st Century. New York, NY: Springer New York; 2010 p. 101–10. 10.1007/978-1-4419-6673-5_7 20795325

[82] Mitreva M, Blaxter ML, Bird DM, McCarter JP. Comparative genomics of nematodes. Trends Genet. 2005;21(10):573–81. doi: 10.1016/j.tig.2005.08.003 . 16099532 10.1016/j.tig.2005.08.003

[83] Yoshinaga K, Yoshioka H, Kurosaki H, Hirasawa M, Uritani M, Hasegawa K. Protection by trehalose of DNA from radiation damage. Biosci Biotechnol Biochem. 1997;61(1):160–1. . 9028044 10.1271/bbb.61.160

[84] Herdeiro RS, Pereira MD, Panek AD, Eleutherio EC. Trehalose protects Saccharomyces cerevisiae from lipid peroxidation during oxidative stress. Biochim Biophys Acta. 2006;1760(3):340–6. doi: 10.1016/j.bbagen.2006.01.010 . 16510250 10.1016/j.bbagen.2006.01.010

[85] Gross V, Mayer G. Neural development in the tardigrade Hypsibius dujardini based on anti-acetylated alpha-tubulin immunolabeling. Evodevo. 2015;6:12 doi: 10.1186/s13227-015-0008-4 ; 26052416 10.1186/s13227-015-0008-4 PMC4458024

[86] Martin C, Gross V, Pflüger H-J, Stevenson PA, Mayer G. Assessing segmental versus non-segmental features in the ventral nervous system of onychophorans (velvet worms). BMC Evolutionary Biology. 2017;17(1):3 doi: 10.1186/s12862-016-0853-3 28049417 10.1186/s12862-016-0853-3 PMC5209844

[87] Sulston JE, Schierenberg E, White JG, Thomson JN. The embryonic cell lineage of the nematode Caenorhabditis elegans. Developmental Biology. 1983;100(1):64–119. doi: 10.1016/0012-1606(83)90201-4 6684600 10.1016/0012-1606(83)90201-4

[88] Andrews S. FastQC a quality-control tool for high-throughput sequence data. 2015 [cited 2015 May 21]. http://www.bioinformatics.babraham.ac.uk/projects/fastqc/.

[89] Edgar RC. Search and clustering orders of magnitude faster than BLAST. Bioinformatics. 2010;26(19):2460–1. doi: 10.1093/bioinformatics/btq461 . 20709691 10.1093/bioinformatics/btq461

[90] Bankevich A, Nurk S, Antipov D, Gurevich AA, Dvorkin M, Kulikov AS, et al SPAdes: a new genome assembly algorithm and its applications to single-cell sequencing. J Comput Biol. 2012;19(5):455–77. doi: 10.1089/cmb.2012.0021 ; 22506599 10.1089/cmb.2012.0021 PMC3342519

[91] O'Leary NA, Wright MW, Brister JR, Ciufo S, Haddad D, McVeigh R, et al Reference sequence (RefSeq) database at NCBI: current status, taxonomic expansion, and functional annotation. Nucleic Acids Res. 2016;44(D1):D733–45. doi: 10.1093/nar/gkv1189 ; 26553804 10.1093/nar/gkv1189 PMC4702849

[92] Langmead B, Salzberg SL. Fast gapped-read alignment with Bowtie 2. Nat Methods. 2012;9(4):357–9. doi: 10.1038/nmeth.1923 ; 22388286 10.1038/nmeth.1923 PMC3322381

[93] English AC, Richards S, Han Y, Wang M, Vee V, Qu J, et al Mind the gap: upgrading genomes with Pacific Biosciences RS long-read sequencing technology. PLoS ONE. 2012;7(11):e47768 doi: 10.1371/journal.pone.0047768 ; 23185243 10.1371/journal.pone.0047768 PMC3504050

[94] Walker BJ, Abeel T, Shea T, Priest M, Abouelliel A, Sakthikumar S, et al Pilon: an integrated tool for comprehensive microbial variant detection and genome assembly improvement. PLoS ONE. 2014;9(11):e112963 doi: 10.1371/journal.pone.0112963 ; 25409509 10.1371/journal.pone.0112963 PMC4237348

[95] Kim D, Pertea G, Trapnell C, Pimentel H, Kelley R, Salzberg SL. TopHat2: accurate alignment of transcriptomes in the presence of insertions, deletions and gene fusions. Genome Biol. 2013;14(4):R36 doi: 10.1186/gb-2013-14-4-r36 ; 23618408 10.1186/gb-2013-14-4-r36 PMC4053844

[96] Borodovsky M, Lomsadze A. Eukaryotic gene prediction using GeneMark.hmm-E and GeneMark-ES. Curr Protoc Bioinformatics. 2011;Chapter 4:Unit 4 6 1–10. doi: 10.1002/0471250953.bi0406s35 ; 21901742 10.1002/0471250953.bi0406s35 PMC3204378

[97] Li H, Durbin R. Fast and accurate short read alignment with Burrows-Wheeler transform. Bioinformatics. 2009;25(14):1754–60. doi: 10.1093/bioinformatics/btp324 ; 19451168 10.1093/bioinformatics/btp324 PMC2705234

[98] Li H, Handsaker B, Wysoker A, Fennell T, Ruan J, Homer N, et al The Sequence Alignment/Map format and SAMtools. Bioinformatics. 2009;25(16):2078–9. doi: 10.1093/bioinformatics/btp352 ; 19505943 10.1093/bioinformatics/btp352 PMC2723002

[99] Okonechnikov K, Conesa A, Garcia-Alcalde F. Qualimap 2: advanced multi-sample quality control for high-throughput sequencing data. Bioinformatics. 2016;32(2):292–4. doi: 10.1093/bioinformatics/btv566 ; 26428292 10.1093/bioinformatics/btv566 PMC4708105

[100] Quinlan AR, Hall IM. BEDTools: a flexible suite of utilities for comparing genomic features. Bioinformatics. 2010;26(6):841–2. doi: 10.1093/bioinformatics/btq033 ; 20110278 10.1093/bioinformatics/btq033 PMC2832824

[101] Mistry J, Finn RD, Eddy SR, Bateman A, Punta M. Challenges in homology search: HMMER3 and convergent evolution of coiled-coil regions. Nucleic Acids Res. 2013;41(12):e121 doi: 10.1093/nar/gkt263 ; 23598997 10.1093/nar/gkt263 PMC3695513

[102] Finn RD, Coggill P, Eberhardt RY, Eddy SR, Mistry J, Mitchell AL, et al The Pfam protein families database: towards a more sustainable future. Nucleic Acids Res. 2016;44(D1):D279–85. doi: 10.1093/nar/gkv1344 ; 26673716 10.1093/nar/gkv1344 PMC4702930

[103] Goujon M, McWilliam H, Li W, Valentin F, Squizzato S, Paern J, et al A new bioinformatics analysis tools framework at EMBL-EBI. Nucleic Acids Res. 2010;38(Web Server issue):W695–9. doi: 10.1093/nar/gkq313 ; 20439314 10.1093/nar/gkq313 PMC2896090

[104] Price AL, Jones NC, Pevzner PA. De novo identification of repeat families in large genomes. Bioinformatics. 2005;21 Suppl 1:i351–8. doi: 10.1093/bioinformatics/bti1018 . 15961478 10.1093/bioinformatics/bti1018

[105] Smit A, Hubley R, Green P. RepeatMasker Open-4.0 2013–2015. http://www.repeatmasker.org.

[106] Pearson WR, Lipman DJ. Improved tools for biological sequence comparison. Proc Natl Acad Sci U S A. 1988;85(8):2444–8. ; 3162770 10.1073/pnas.85.8.2444 PMC280013

[107] Katoh K, Standley DM. MAFFT multiple sequence alignment software version 7: improvements in performance and usability. Mol Biol Evol. 2013;30(4):772–80. doi: 10.1093/molbev/mst010 ; 23329690 10.1093/molbev/mst010 PMC3603318

[108] Love MI, Huber W, Anders S. Moderated estimation of fold change and dispersion for RNA-seq data with DESeq2. Genome Biol. 2014;15(12):550 doi: 10.1186/s13059-014-0550-8 ; 25516281 10.1186/s13059-014-0550-8 PMC4302049

[109] Bray NL, Pimentel H, Melsted P, Pachter L. Near-optimal probabilistic RNA-seq quantification. Nat Biotechnol. 2016;34(5):525–7. doi: 10.1038/nbt.3519 . 27043002 10.1038/nbt.3519

[110] Okuda S, Yamada T, Hamajima M, Itoh M, Katayama T, Bork P, et al KEGG Atlas mapping for global analysis of metabolic pathways. Nucleic Acids Res. 2008;36(Web Server issue):W423–6. doi: 10.1093/nar/gkn282 ; 18477636 10.1093/nar/gkn282 PMC2447737

[111] Darling AC, Mau B, Blattner FR, Perna NT. Mauve: multiple alignment of conserved genomic sequence with rearrangements. Genome Res. 2004;14(7):1394–403. doi: 10.1101/gr.2289704 ; 15231754 10.1101/gr.2289704 PMC442156

[112] Price MN, Dehal PS, Arkin AP. FastTree 2—approximately maximum-likelihood trees for large alignments. PLoS ONE. 2010;5(3):e9490 doi: 10.1371/journal.pone.0009490 ; 20224823 10.1371/journal.pone.0009490 PMC2835736

[113] Rambaut A. FigTree. 2016.

[114] Howe KL, Bolt BJ, Shafie M, Kersey P, Berriman M. WormBase ParaSite—a comprehensive resource for helminth genomics. Mol Biochem Parasitol. 2016 doi: 10.1016/j.molbiopara.2016.11.005 . 27899279 10.1016/j.molbiopara.2016.11.005 PMC5486357

[115] Howe KL, Bolt BJ, Cain S, Chan J, Chen WJ, Davis P, et al WormBase 2016: expanding to enable helminth genomic research. Nucleic Acids Res. 2016;44(D1):D774–80. doi: 10.1093/nar/gkv1217 ; 26578572 10.1093/nar/gkv1217 PMC4702863

[116] Sievers F, Wilm A, Dineen D, Gibson TJ, Karplus K, Li W, et al Fast, scalable generation of high-quality protein multiple sequence alignments using Clustal Omega. Mol Syst Biol. 2011;7(539):539 doi: 10.1038/msb.2011.75 ; 21988835 10.1038/msb.2011.75 PMC3261699

[117] Huang X. CAP3: A DNA Sequence Assembly Program. Genome Research. 1999;9(9):868–77. doi: 10.1101/gr.9.9.868 10508846 10.1101/gr.9.9.868 PMC310812

[118] Jiang H, Lei R, Ding SW, Zhu S. Skewer: a fast and accurate adapter trimmer for next-generation sequencing paired-end reads. BMC Bioinformatics. 2014;15:182 doi: 10.1186/1471-2105-15-182 ; 24925680 10.1186/1471-2105-15-182 PMC4074385

[119] Haas BJ, Papanicolaou A. TransDecoder (Find Coding Regions Within Transcripts).

[120] Capella-Gutierrez S, Silla-Martinez JM, Gabaldon T. trimAl: a tool for automated alignment trimming in large-scale phylogenetic analyses. Bioinformatics. 2009;25(15):1972–3. doi: 10.1093/bioinformatics/btp348 ; 19505945 10.1093/bioinformatics/btp348 PMC2712344

[121] Kuck P, Longo GC. FASconCAT-G: extensive functions for multiple sequence alignment preparations concerning phylogenetic studies. Front Zool. 2014;11(1):81 doi: 10.1186/s12983-014-0081-x ; 25426157 10.1186/s12983-014-0081-x PMC4243772

[122] Lartillot N, Philippe H. A Bayesian mixture model for across-site heterogeneities in the amino-acid replacement process. Mol Biol Evol. 2004;21(6):1095–109. doi: 10.1093/molbev/msh112 . 15014145 10.1093/molbev/msh112

[123] Challis RJ, Kumar S, Stevens L, Blaxter M. EasyMirror and EasyImport: Simplifying the setup of a custom Ensembl database and webserver for any species. PeerJ Preprints. 2016;4(e2401v1). doi: 10.7287/peerj.preprints.2401v1

[124] Arakawa K, Mori K, Ikeda K, Matsuzaki T, Kobayashi Y, Tomita M. G-language Genome Analysis Environment: a workbench for nucleotide sequence data mining. Bioinformatics. 2003;19(2):305–6. . 12538262 10.1093/bioinformatics/19.2.305

[125] Arakawa K, Tomita M. G-language system as a platform for large-scale analysis of high-throughput omics data. J Pestic Sci. 2006;31(3):282–8. doi: 10.1584/jpestics.31.282

