# Supplementary material for: Comparative genomics of the tardigrades Hypsibius dujardini and Ramazzottius varieornatus
Source: PLoS Biol. 2017 Jul 27;15(7):e2002266. doi: 10.1371/journal.pbio.2002266 (PMC5531438; doi:10.1371/journal.pbio.2002266)
Supplement: S1 Fig — (DOCX) [file pbio.2002266.s001.docx]

S1 Fig. DNA sequencing coverage of the H. dujardini genome

Single individual DNA sequencing data (DRR055040) were mapped to the *H. dujardini*  genome with BWA MEM, and the genomic coverage was calculated with Qualimap bamqc. Scaffolds were concatenated, sorted by length (longest first).
