# Supplementary material for: Comparative genomics of the tardigrades Hypsibius dujardini and Ramazzottius varieornatus
Source: PLoS Biol. 2017 Jul 27;15(7):e2002266. doi: 10.1371/journal.pbio.2002266 (PMC5531438; doi:10.1371/journal.pbio.2002266)
Supplement: S2 Fig — (DOCX) [file pbio.2002266.s002.docx]

S2 Fig. tRNA genes in *Hypsibius dujardini* and *Ramazzottius varieornatus*

tRNA loci were predicted with tRNA-Scan SE. Each codon is colored by the number of tRNA loci found.
