# Supplementary material for: Comparative genomics of the tardigrades Hypsibius dujardini and Ramazzottius varieornatus
Source: PLoS Biol. 2017 Jul 27;15(7):e2002266. doi: 10.1371/journal.pbio.2002266 (PMC5531438; doi:10.1371/journal.pbio.2002266)
Supplement: S3 Fig — (DOCX) [file pbio.2002266.s003.docx]

S3 Fig. Comparisons of genic features between *Hypsibius dujardini* and *Ramazzottius varieornatus*


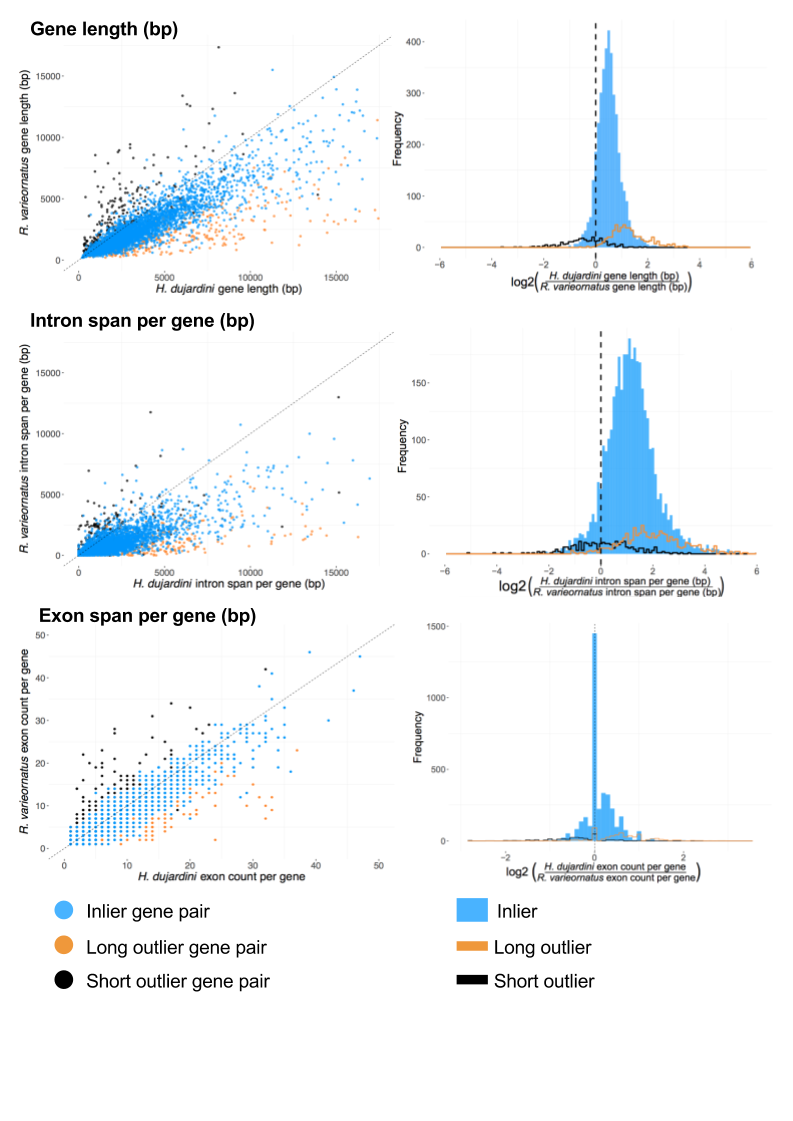


Comparisons of genes structures in 4728 single-copy orthologues between *H. dujardini* and *R. varieornatus.* Outliers are defined as genes in *H. dujardini* which have CDS lengths 20% longer (long outliers; orange; 576 genes) or 20% shorter (short outliers; black; 294 genes) than their orthologues in *R. varieornatus.* Left panels show ascatter plots, with *H. dujardini* on the X and *R. varieornatus* on the Y axes. Right panels show frequency histograms of the ratios of genic feature lengths per gene. A positive log2 ratio indicates a trend towards a larger count or span in *H. dujardini*.
