# Supplementary material for: Comparative genomics of the tardigrades Hypsibius dujardini and Ramazzottius varieornatus
Source: PLoS Biol. 2017 Jul 27;15(7):e2002266. doi: 10.1371/journal.pbio.2002266 (PMC5531438; doi:10.1371/journal.pbio.2002266)
Supplement: S4 Fig — (DOCX) [file pbio.2002266.s004.docx]

S4 Fig. Clustered HGT loci in *H. dujardini* and *R. varieornatus*


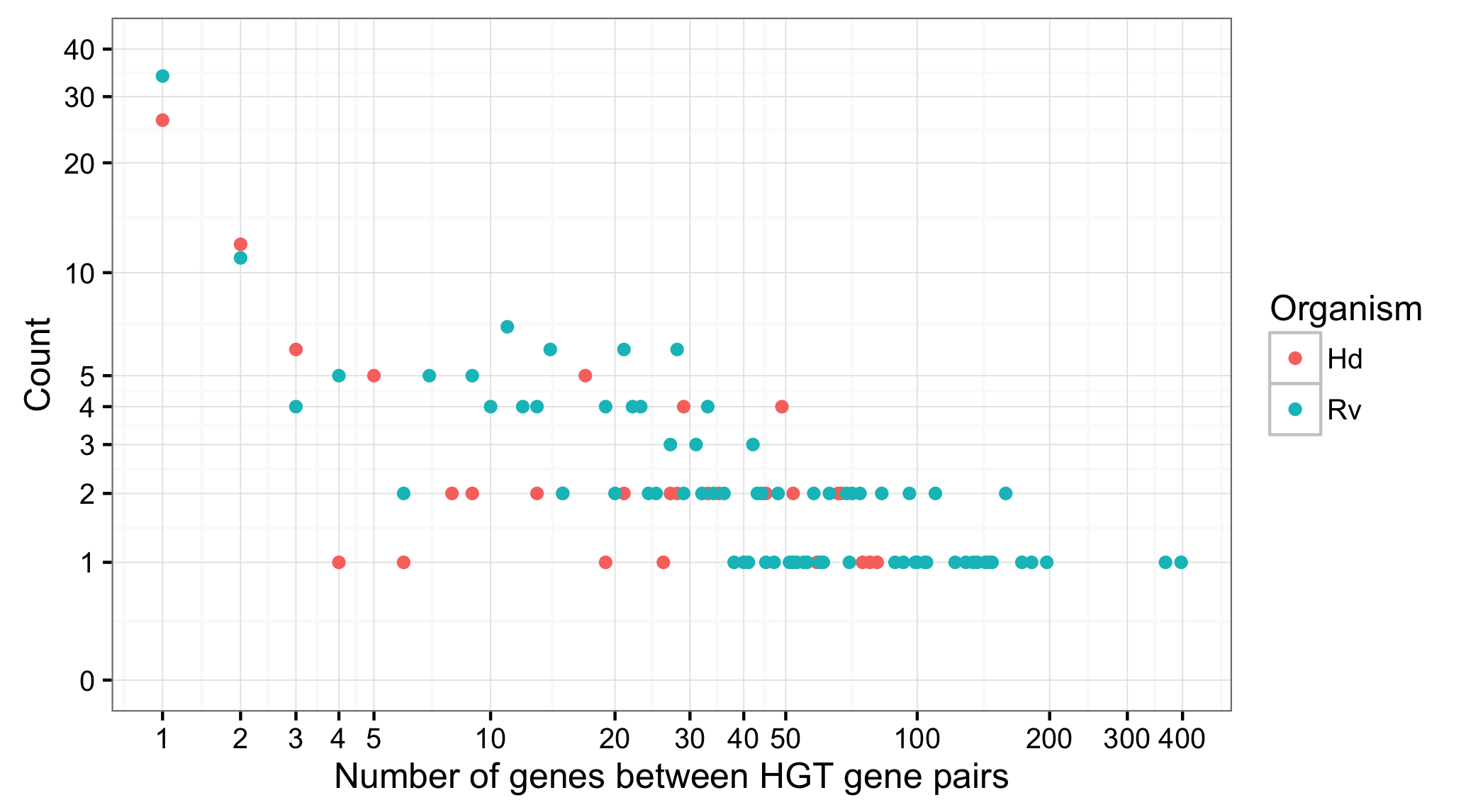


HGT candidates are clustered in the genomes of *H. dujardini* and *R. varieornatus*. The number of genes between each HGT locus was calculated, and a frequency of separations calculated. A separation of 1 indicates that the genes are neighbours. Hd=*H. dujardini*, Rv=*R. varieornatus*. Data available at https://github.com/abs-yy/Hypsibius_dujardini_manuscript/blob/master/data/FigS4_hgt_neighborhood.txt.
