# Supplementary material for: Comparative genomics of the tardigrades Hypsibius dujardini and Ramazzottius varieornatus
Source: PLoS Biol. 2017 Jul 27;15(7):e2002266. doi: 10.1371/journal.pbio.2002266 (PMC5531438; doi:10.1371/journal.pbio.2002266)
Supplement: S5 Fig — (DOCX) [file pbio.2002266.s005.docx]

S5 Fig. Biochemical pathways acquired or supplemented by HGT in *H. dujardini* and *R. varieornatus*.

Trehalose and ascorbate synthesis pathways were reconstructed with KAAS and KEGG mapper.

A Ascorbate synthesis. Many genes in this pathway were derived through HGT (red arrows).

B Trehalose synthesis. *H. dujardini* lacks TPS loci (grey lozenges) but contains treA trehalase loci (blue lozenges). Both enzymes are present in *R. varieornatus*.
