# Supplementary material for: Comparative genomics of the tardigrades Hypsibius dujardini and Ramazzottius varieornatus
Source: PLoS Biol. 2017 Jul 27;15(7):e2002266. doi: 10.1371/journal.pbio.2002266 (PMC5531438; doi:10.1371/journal.pbio.2002266)
Supplement: S6 Fig — (DOCX) [file pbio.2002266.s006.docx]

S6 Fig. Phylogeny of protection-related proteins

The amino acid sequences of CAHS, SAHS, MAHS, RvLEAM and Dsup genes in *H. dujardini* and *R. varieornatus* were aligned with ClustalW2, and a phylogenetic tree was inferred with FastTree with 1,000 bootstraps. Each clade was annotated with the corresponding subtype as defined in *R. varieornatus* (CAHS1:g673, CAHS2:g675, CAHS3:g884, SAHS1:g1671, SAHS2:g1676, MAHS:g6834, RvLEAM:g2978, Dsup:g4591).
