# Supplementary material for: Comparative genomics of the tardigrades Hypsibius dujardini and Ramazzottius varieornatus
Source: PLoS Biol. 2017 Jul 27;15(7):e2002266. doi: 10.1371/journal.pbio.2002266 (PMC5531438; doi:10.1371/journal.pbio.2002266)
Supplement: S1 Table — (DOCX) [file pbio.2002266.s007.docx]

S1 Table. Data used in this study

(A) DNA sequencing

| Origin | Previous work | This work | Boothby, *et al.* PNAS, 2015 | | |
| --- | --- | --- | --- | --- | --- |
| Accession ID | DRR055040 | SRX2495681 | SRX1474871 | SRX1474929 | SRX1474950 |
| Platform | MiSeq | PacBio | HiSeq 2000 | HiSeq 2000 | HiSeq 2000 |
| Number of Reads | 51,607,261 | 779,905 | 87,744,967 | 64,049,630 | 45,733,238 |
| Number of raw bases (Gbase) | 15.5 | 5.88 | 8.8 | 6.4 | 4.6 |
| Read Length | 300 b paired | (variable) | 100 b paired | 100 b paired | 100 b paired |
| Maximum length | NA | 49,455 | NA | NA | NA |
| N50 length | NA | 10,657 | NA | NA | NA |
| Average length | NA | 7,536 | NA | NA | NA |
| Insert length | 477.1 | NA | 347.6 | 496.9 | 749.3 |

We generated new sequencing data using PacBio SMRT technology. In addition, we have used sequence data from Boothby *et al* [1] for assembly, and single individual sequencing data from our previous report [2].

**(B) *Hypsibius dujardini* RNA-Seq**

| #Individuals | Platform | sample | #replicate | Sample ID | #Reads | #Accession |
| --- | --- | --- | --- | --- | --- | --- |
| 10,000 | HiSeq2000 | Active | 1 | Active_1 | 25,172,359 | SRX2528369 |
|  |  |  | 2 | Active_2 | 26,497,216 | SRX2528370 |
|  |  |  | 3 | Active_3 | 28,141,582 | SRX2528371 |
|  |  | Tun | 1 | Tun_1 | 25,782,478 | SRX2528372 |
|  |  |  | 2 | Tun_2 | 27,832,551 | SRX2528373 |
|  |  |  | 3 | Tun_3 | 27,001,002 | SRX2528374 |
| 30 | NextSeq500 | Active | 1 | act-1 | 11,399,144 | SRX2528375 |
|  |  |  | 2 | act-2 | 10,744,670 | SRX2528376 |
|  |  |  | 3 | act-3 | 10,939,323 | SRX2528377 |
|  |  | Tun | 1 | tun-1 | 10,325,677 | SRX2528378 |
|  |  |  | 2 | tun-2 | 10,689,489 | SRX2528379 |
|  |  |  | 3 | tun-3 | 10,455,913 | SRX2528380 |
|  |  | Egg 1st day after laying | 1 | H-E1-1 | 8,822,054 | SRX2528333 |
|  |  |  | 2 | H-E1-2 | 10,286,604 | SRX2528334 |
|  |  |  | 3 | H-E1-3 | 8,319,242 | SRX2528335 |
|  |  | Egg 2nd day after laying | 1 | H-E2-1 | 11,794,526 | SRX2528336 |
|  |  |  | 2 | H-E2-2 | 11,086,054 | SRX2528337 |
|  |  |  | 3 | H-E2-3 | 10,151,210 | SRX2528338 |
|  |  | Egg 3rd day after laying | 1 | H-E3-1 | 10,057,550 | SRX2528339 |
|  |  |  | 2 | H-E3-2 | 9,253,951 | SRX2528340 |
|  |  |  | 3 | H-E3-3 | 11,871,780 | SRX2528341 |
|  |  | Egg 4th day after laying | 1 | H-E4-1 | 11,622,113 | SRX2528342 |
|  |  |  | 2 | H-E4-2 | 12,386,383 | SRX2528343 |
|  |  |  | 3 | H-E4-3 | 9,654,776 | SRX2528344 |
|  |  | Egg 5th day after laying | 1 | H-E5-1 | 11,921,100 | SRX2528345 |
|  |  |  | 2 | H-E5-2 | 11,569,382 | SRX2528346 |
|  |  |  | 3 | H-E5-3 | 10,503,387 | SRX2528347 |
|  |  | Juvenile 1st day | 1 | H-B1-1 | 12,440,551 | SRX2528348 |
|  |  |  | 2 | H-B1-2 | 12,306,138 | SRX2528349 |
|  |  |  | 3 | H-B1-3 | 12,734,126 | SRX2528350 |
|  |  | Juvenile 2nd day | 1 | H-B2-1 | 13,107,156 | SRX2528351 |
|  |  |  | 2 | H-B2-2 | 14,437,609 | SRX2528352 |
|  |  |  | 3 | H-B2-3 | 13,870,809 | SRX2528353 |
|  |  | Juvenile 3rd day | 1 | H-B3-1 | 8,360,076 | SRX2528354 |
|  |  |  | 2 | H-B3-2 | 6,542,790 | SRX2528355 |
|  |  |  | 3 | H-B3-3 | 9,775,113 | SRX2528356 |
|  |  | Juvenile 4th day | 1 | H-B4-1 | 9,824,335 | SRX2528357 |
|  |  |  | 2 | H-B4-2 | 16,666,875 | SRX2528358 |
|  |  |  | 3 | H-B4-3 | 15,995,271 | SRX2528359 |
|  |  | Juvenile 5th day | 1 | H-B5-1 | 6,928,823 | SRX2528360 |
|  |  |  | 2 | H-B5-2 | 8,857,975 | SRX2528361 |
|  |  |  | 3 | H-B5-3 | 12,901,947 | SRX2528362 |
|  |  | Juvenile 6th day | 1 | H-B6-1 | 9,843,726 | SRX2528363 |
|  |  |  | 2 | H-B6-2 | 12,913,346 | SRX2528364 |
|  |  |  | 3 | H-B6-3 | 11,745,564 | SRX2528365 |
|  |  | Juvenile 7th day | 1 | H-B7-1 | 12,384,307 | SRX2528366 |
|  |  |  | 2 | H-B7-2 | 9,182,107 | SRX2528367 |
|  |  |  | 3 | H-B7-3 | 13,626,269 | SRX2528368 |
| 5,000 | HiSeq2000 | miRNA-Seq | 1 | HD_miRNA | 32,254,413 | SRX2495676 |

**(C) *Ramazzottius varieornatus* RNA-Seq**

| #Individuals | Platform | sample | #rep | Sample ID | #Reads | #Accession |
| --- | --- | --- | --- | --- | --- | --- |
| 1~2.5 | NextSeq500 | Active-Fast | 1 | Y-active_slow_1 | 12,146,289 | SRX2528399 |
|  |  |  | 2 | Y-active_slow_2 | 11,076,841 | SRX2528400 |
|  |  |  | 3 | Y-active_slow_3 | 11,211,443 | SRX2528401 |
|  |  | Tun-Fast | 1 | Y-tun_slow_ 1 | 11,781,529 | SRX2528402 |
|  |  |  | 2 | Y-tun_slow_2 | 11,966,104 | SRX2528403 |
|  |  |  | 3 | Y-tun_slow_3 | 12,361,848 | SRX2528404 |
| 30 |  | Active-Slow | 1 | Y-active_fast_1 | 31,330,380 | SRX2528405 |
|  |  |  | 2 | Y-active_fast_2 | 35,320,831 | SRX2528406 |
|  |  |  | 3 | Y-active_fast_3 | 36,895,441 | SRX2528407 |
|  |  | Tun-Slow | 1 | Y-tun_fast_1 | 35,469,871 | SRX2528408 |
|  |  |  | 2 | Y-tun_fast_ 2 | 38,879,671 | SRX2528409 |
|  |  |  | 3 | Y-tun_fast_ 3 | 31,835,650 | SRX2528410 |
|  |  | Egg 1st day after laying | 1 | Y-E1-1 | 11,688,367 | SRX2528381 |
|  |  |  | 2 | Y-E1-2 | 13,064,048 | SRX2528382 |
|  |  |  | 3 | Y-E1-3 | 13,389,666 | SRX2528383 |
|  |  | Egg 2nd day after laying | 1 | Y-E2-1 | 12,702,879 | SRX2528384 |
|  |  |  | 2 | Y-E2-2 | 14,385,811 | SRX2528385 |
|  |  |  | 3 | Y-E2-3 | 13,101,271 | SRX2528386 |
|  |  | Egg 3rd day after laying | 1 | Y-E3-1 | 14,348,899 | SRX2528387 |
|  |  |  | 2 | Y-E3-2 | 13,640,410 | SRX2528388 |
|  |  |  | 3 | Y-E3-3 | 8,817,117 | SRX2528389 |
|  |  | Egg 4th day after laying | 1 | Y-E4-1 | 12,606,663 | SRX2528390 |
|  |  |  | 2 | Y-E4-2 | 15,271,225 | SRX2528391 |
|  |  |  | 3 | Y-E4-3 | 12,517,722 | SRX2528392 |
|  |  | Egg 5th day after laying | 1 | Y-E5-1 | 12,599,958 | SRX2528393 |
|  |  |  | 2 | Y-E5-2 | 14,476,417 | SRX2528394 |
|  |  |  | 3 | Y-E5-3 | 17,324,895 | SRX2528395 |
|  |  | Juvenile 1st day | 1 | Y-B1-1 | 4,811,886 | SRX2528396 |
|  |  |  | 2 | Y-B1-2 | 6,210,798 | SRX2528397 |
|  |  |  | 3 | Y-B1-3 | 5,637,785 | SRX2528398 |

### **Reference**

1. Boothby TC, Tenlen JR, Smith FW, Wang JR, Patanella KA, Nishimura EO, et al. Evidence for extensive horizontal gene transfer from the draft genome of a tardigrade. Proc Natl Acad Sci U S A. 2015;112(52):15976-15981. doi: 10.1073/pnas.1510461112.

2. Arakawa K, Yoshida Y, Tomita M. Genome sequencing of a single tardigrade *Hypsibius dujardini* individual. Sci Data. 2016;3:160063. doi: 10.1038/sdata.2016.63.
