# Supplementary material for: Comparative genomics of the tardigrades Hypsibius dujardini and Ramazzottius varieornatus
Source: PLoS Biol. 2017 Jul 27;15(7):e2002266. doi: 10.1371/journal.pbio.2002266 (PMC5531438; doi:10.1371/journal.pbio.2002266)
Supplement: S2 Table — (DOCX) [file pbio.2002266.s008.docx]

S2 Table. Mapping statistics of DNA sequencing data

| Origin* | Accession ID | Mapped Reads | Coverage  (1^st^ quantile, Median, 3^rd^ quantile) | # Insertions/Deletions |
| --- | --- | --- | --- | --- |
| UNC | SRR2986339 | 47.6M (54.24%) | 36/48/56 | 6.85M/7.70M |
|  | SRR2986435 | 53.8M (83.95%) | 40/53/62 | 1.84M/2.02 |
|  | SRR2986451 | 39.2M (85.68%) | 26/37/46 | 2.82M/2.90M |
| Edinburgh | ERR1147177 | 116.0M (77.15%) | 61/98/134 | 3.2M/3.32M |
|  | ERR1147178 | 99.2M (80.03%) | 22/50/70 | 2.30M/2.37M |
| Keio | DRR055040 | 50.0M (96.91%) | 66/109/153 | 8.59M/8/48 |

UNC = University of North Carolina [1]; Edinburgh = University of Edinburgh [2]; Keio = Keio University (this work and [3])

### **Reference**

1. Boothby TC, Tenlen JR, Smith FW, Wang JR, Patanella KA, Nishimura EO, et al. Evidence for extensive horizontal gene transfer from the draft genome of a tardigrade. Proc Natl Acad Sci U S A. 2015;112(52):15976-15981. doi: 10.1073/pnas.1510461112.

2. Koutsovoulos G, Kumar S, Laetsch DR, Stevens L, Daub J, Conlon C, et al. No evidence for extensive horizontal gene transfer in the genome of the tardigrade Hypsibius dujardini. Proc Natl Acad Sci U S A. 2016;113(18):5053-5058. doi: 10.1073/pnas.1600338113.

3. Arakawa K, Yoshida Y, Tomita M. Genome sequencing of a single tardigrade *Hypsibius dujardini* individual. Sci Data. 2016;3:160063. doi: 10.1038/sdata.2016.63.
