# Supplementary material for: Comparative genomics of the tardigrades Hypsibius dujardini and Ramazzottius varieornatus
Source: PLoS Biol. 2017 Jul 27;15(7):e2002266. doi: 10.1371/journal.pbio.2002266 (PMC5531438; doi:10.1371/journal.pbio.2002266)
Supplement: S3 Table — (DOCX) [file pbio.2002266.s009.docx]

S3 Table. Repeat content of the genomes of *Hypsibius dujardini* and *Ramazzottius varieornatus*

| Category | Term | *H. dujardini* | *R. varieornatus* |
| --- | --- | --- | --- |
| Simple | #elements | 65,638 | 3,301 |
|  | #length (bp) | 5,391,682 | 137,297 |
|  | %genome | 5.18 | 0.25 |
| Unclassified | #elements | 158,522 | 65,730 |
|  | #length (bp) | 24,232,698 | 11,020,138 |
|  | %genome | 23.27 | 19.74 |
| Total | #elements | 224,160 | 69,031 |
|  | #length (bp) | 29,624,380 | 11,157,435 |
|  | %genome | 28.45 | 19.99 |
