# Supplementary material for: Comparative genomics of the tardigrades Hypsibius dujardini and Ramazzottius varieornatus
Source: PLoS Biol. 2017 Jul 27;15(7):e2002266. doi: 10.1371/journal.pbio.2002266 (PMC5531438; doi:10.1371/journal.pbio.2002266)
Supplement: S4 Table — (DOCX) [file pbio.2002266.s010.docx]

S4 Table. Low-complexity and possible telomeric repeats in the *H. dujardini* genome

| Scaffold* | Start | End | Repeat | Length from End | Length |
| --- | --- | --- | --- | --- | --- |
| scaffold0088 | 327857 | 336800 | TTGATGGGTT | 49 | 8943 |
| scaffold0114 | 15 | 7307 | ATCAAAACCC | 15 | 7292 |
| scaffold0012 | 1 | 5955 | CATCAAAACC | 1 | 5954 |
| scaffold0363 | 14 | 4481 | ATCAAAACCC | 14 | 4467 |
| scaffold0321 | 157 | 4157 | AAAACCCATC | 157 | 4000 |
| scaffold0005 | 52 | 3367 | CAAAACCCAT | 52 | 3315 |
| scaffold0128 | 239844 | 242599 | GGTTTTGATG | 823 | 2755 |
| scaffold0001 | 7702 | 9727 | AAACCCATCA | 7702 | 2025 |
| scaffold0192 | 164482 | 165621 | GGGTTTTGAT | 49 | 1139 |
| scaffold0343 | 22767 | 23510 | TTTTGATGGG | 22767 | 743 |
| scaffold0023 | 54017 | 54373 | ATCAAAACCC | 54017 | 356 |
| scaffold0287 | 57943 | 58286 | ATGGGTTTTG | 36758 | 343 |
| scaffold0212 | 65340 | 65622 | TTTTGATGGG | 65340 | 282 |
| scaffold0093 | 201990 | 202227 | TTGATGGGTT | 107275 | 237 |
| scaffold0072 | 51074 | 51288 | TGGGTTTTGA | 51074 | 214 |
| scaffold0070 | 189916 | 190113 | CATCAAAACC | 189916 | 197 |
| scaffold0031 | 383706 | 383897 | GATGGGTTTT | 227004 | 191 |
| scaffold0090 | 105640 | 105790 | TCAAAACCCA | 105640 | 150 |
| scaffold0092 | 52492 | 52623 | ATGGGTTTTG | 52492 | 131 |
| scaffold0005 | 3799 | 3922 | ATCAAAACCC | 3799 | 123 |
| scaffold0036 | 185539 | 185660 | ATCAAAACCC | 185539 | 121 |
| scaffold0036 | 17528 | 17649 | ATCAAAACCC | 17528 | 121 |
| scaffold0070 | 137262 | 137380 | AAACCCATCA | 137262 | 118 |
| scaffold0117 | 133706 | 133813 | ATCAAAACCC | 120682 | 107 |
| scaffold0171 | 121075 | 121176 | AAACCCATCA | 66249 | 101 |
| scaffold0268 | 65742 | 65843 | ATCAAAACCC | 36714 | 101 |

Regions close to scaffold ends are highlighted in yellow; these may represent telomeric ends.
