# Supplementary material for: Comparative genomics of the tardigrades Hypsibius dujardini and Ramazzottius varieornatus
Source: PLoS Biol. 2017 Jul 27;15(7):e2002266. doi: 10.1371/journal.pbio.2002266 (PMC5531438; doi:10.1371/journal.pbio.2002266)
Supplement: S5 Table — (DOCX) [file pbio.2002266.s011.docx]

S5 Table. Mapping proportions of RNA-Seq data

(A) Anhydrobiosis samples

| ***Hypsibius dujardini*** | | | | |
| --- | --- | --- | --- | --- |
| **10k Individuals** | | *H. dujardini* transcriptome | *H. dujardini* genome |  |
| act-1 | | 35,536,088 (70.08%) | 51,790,544 (92.95%) |  |
| act-2 | | 38,335,541 (71.78%) | 55,208,279 (93.93%) |  |
| act-3 | | 40,570,560 (71.53%) | 58,667,531 (94.03%) |  |
| tun-1 | | 37,104,384 (71.41%) | 54,280,566 (94.77%) |  |
| tun-2 | | 39,611,771 (70.58%) | 58,602,174 (94.87%) |  |
| tun-3 | | 38,118,839 (69.87%) | 57,146,961 (95.28%) |  |
| **30 Individuals** | | *H. dujardini* transcriptome |  |  |
| act-1 | | 4,276,18 (66.19%) |  |  |
| act-2 | | 5,604,724 (52.04%) |  |  |
| act-3 | | 2,628,272 (24.98%) |  |  |
| tun-1 | | 6,410,871 (61.75%) |  |  |
| tun-2 | | 4,524,015 (61.87%) |  |  |
| tun-3 | | 5,562,719 (61.55%) |  |  |
| ***Ramazzottius varieornatus*** | | | | |
| **Slow-dry** | *R.* varieornatus transcriptome | | *R. varieornatus* genome | Hashimoto *et al.* gene model [1] |
| act-1 | 6,414,974 (52.68%) | | 11,459,570 (90.77%) | 6,592920 (54.14%) |
| act-2 | 6,052,043 (54.49%) | | 10,525516 (91.22%) | 6,199,903 (55.82%) |
| act-3 | 5,587,457 (49.73%) | | 10,581005 (90.96%) | 5,735,575 (51.04%) |
| tun-1 | 5,327,210 (45.14%) | | 10,816699 (88.77%) | 5,484,698 (46.47%) |
| tun-2 | 5,593,452 (46.66%) | | 11,003,773 (88.82%) | 5,754,535 (48.00%) |
| tun-3 | 6,108,721 (49.31%) | | 11,614,986 (90.71%) | 6,287,399 (50.75%) |
| **Fast-dry** | *R.* varieornatus transcriptome | | *R. varieornatus* genome | Hashimoto *et al.* gene model [1] |
| act-1 | 15,088,390 (48.06%) | |  |  |
| act-2 | 17,576,037 (49.66%) | |  |  |
| act-3 | 17,942,032 (48.53%) | |  |  |
| tun-1 | 11,430,186 (32.18%) | |  |  |
| tun-2 | 13,773,138 (35.37%) | |  |  |
| tun-3 | 12,529,323 (39.30%) | |  |  |

(B) Developmental samples

| Development | *Hypsibius dujardini* | | *Ramazzottius varieornatus* | |
| --- | --- | --- | --- | --- |
|  | *Transcriptome* | *Genome* | *Transcriptome* | *Genome* |
| E1-1 | 4,378,145 (49.51%) | 7,962,696 (87.17%) | 5,191,424 (44.32%) | 9,436,055 (77.92%) |
| E1-2 | 5,253,306 (50.92%) | 9,311,381 (87.33%) | 5,299,355 (40.49%) | 9,693,795 (71.78%) |
| E1-3 | 3,958,477 (47.46%) | 7,341,290 (84.97%) | 5,051,568 (37.66%) | 9,497,572 (68.66%) |
| E2-1 | 4,818,034 (40.76%) | 10,213,359 (83.77%) | 2,591,777 (20.38%) | 5,835,138 (45.13%) |
| E2-2 | 4,777,963 (42.98%) | 9,834,385 (85.84%) | 3,098,601 (21.52%) | 6,685,797 (45.59%) |
| E2-3 | 4,525,795 (44.45%) | 8,777,764 (83.40%) | 2,514,170 (19.17%) | 5,823252 (43.70%) |
| E3-1 | 4,529,697 (44.92%) | 8,738,778 (83.96%) | 3,504,071 (24.40%) | 7,761,912 (53.03%) |
| E3-2 | 3,661,789 (39.49%) | 7,335,588 (76.93%) | 2,935,065 (21.50%) | 6,961,489 (50.14%) |
| E3-3 | 4,941,305 (41.53%) | 10,139,902 (82.78%) | 1,551,655 (17.59%) | 3,826,220 (42.74%) |
| E4-1 | 4,,446,480 (38.18%) | 8,251,277 (68.94%) | 2,286,988 (18.13%) | 5,335,818 (41.70%) |
| E4-2 | 5,057,162 (40.74%) | 9,514,858 (74.52%) | 1,805,923 (11.82%) | 4,599,738 (29.82%) |
| E4-3 | 3,622,851 (37.44%) | 7,249,760 (73.06%) | 1,451,975 (11.59%) | 3,634,522 (28.75%) |
| E5-1 | 5,008,318 (41.91%) | 9,568,902 (77.70%) | 9,29,244 (7.37%) | 2,786,443 (21.98%) |
| E5-2 | 5,415,199 (46.66%) | 10,173,646 (84.90%) | 2,99,688 (2.07%) | 1,680,860 (11.58%) |
| E5-3 | 4,816,060 (45.72%) | 9,050,720 (83.26%) | 6,30,014 (3.64%) | 2,605,069 (14.98%) |
| B1-1 | 6,372,485 (51.09%) | 11,862,133 (91.80%) | 2,043,701 (42.39%) | 3,563,016 (71.91%) |
| B1-2 | 3,753,075 (30.46%) | 7,981,613 (63.46%) | 2,871,856 (46.14%) | 4832,785 (75.38%) |
| B1-3 | 4,574,819 (35.86%) | 9,315,606 (71.27%) | 2,684,092 (47.50%) | 4,539,890 (77.94%) |
| B2-1 | 2,727,779 (20.79%) | 6,397,464 (48.10%) | NA | NA |
| B2-2 | 2,293,308 (15.87%) | 5,574,352 (38.18%) | NA | NA |
| B2-3 | 2,244,975 (16.17%) | 5,219,405 (37.19%) | NA | NA |
| B3-1 | 3,009,036 (35.94%) | 6,134,894 (71.71%) | NA | NA |
| B3-2 | 1,800,045 (27.48%) | 3,885,947 (58.34%) | NA | NA |
| B3-3 | 3,070,949 (31.38%) | 6,447,666 (64.60%) | NA | NA |
| B4-1 | 3,213,530 (32.66%) | 6,355,544 (63.35%) | NA | NA |
| B4-2 | 3,718,153 (22.29%) | 8,188,239 (48.43%) | NA | NA |
| B4-3 | 3,808,703 (23.79%) | 8,102,210 (49.89%) | NA | NA |
| B5-1 | 2,366,172 (34.10%) | 4,775,355 (67.40%) | NA | NA |
| B5-2 | 2,507,768 (28.28%) | 5,510,508 (61.17%) | NA | NA |
| B5-3 | 3,797,968 (29.40%) | 7,968,128 (60.63%) | NA | NA |
| B6-1 | 4,176,593 (42.35%) | 8,477,432 (83.80%) | NA | NA |
| B6-2 | 5,616,490 (43.40%) | 11,116,147 (83.69%) | NA | NA |
| B6-3 | 4,280,233 (36.37%) | 8,557,430 (71.17%) | NA | NA |
| B7-1 | 5,380,476 (43.35%) | 10,719,826 (84.09%) | NA | NA |
| B7-2 | 4,177,942 (45.40%) | 8,118,862 (85.79%) | NA | NA |
| B7-3 | 5,987,512 (43.85%) | 11,144,850 (79.27%) | NA | NA |

**Reference**

1. Hashimoto T, Horikawa DD, Saito Y, Kuwahara H, Kozuka-Hata H, Shin IT, et al. Extremotolerant tardigrade genome and improved radiotolerance of human cultured cells by tardigrade-unique protein. Nat Commun. 2016;7:12808. doi: 10.1038/ncomms12808.
