# Supplementary material for: Comparative genomics of the tardigrades Hypsibius dujardini and Ramazzottius varieornatus
Source: PLoS Biol. 2017 Jul 27;15(7):e2002266. doi: 10.1371/journal.pbio.2002266 (PMC5531438; doi:10.1371/journal.pbio.2002266)
Supplement: S6 Table — (DOCX) [file pbio.2002266.s012.docx]

S6 Table. Mapping proportion of the *Hypsibius dujardini* Trinity assembled transcriptome

| ***Hypsibius dujardini*** |  |  |  |
| --- | --- | --- | --- |
| **10k Individuals** | Total number of transcripts assembled | Number (proportion) mapped to the *H. dujardini* transcriptome | Number (proportion) mapped to the *H. dujardini* genome |
| act-1 | 66886 | 43608 (65.20%) | 59283 (88.63%) |
| act-2 | 68941 | 45168 (65.52%) | 60998 (88.48%) |
| act-3 | 73982 | 52217 (70.58%) | 68767 (99.74%) |
| tun-1 | 63670 | 43667 (68.58%) | 59503 (93.46%) |
| tun-2 | 77919 | 54768 (61.59%) | 74304 (95.37%) |
| tun-3 | 149853 | 72078 (48.10%) | 117883 (78.67%) |
