# Supplementary material for: Comparative genomics of the tardigrades Hypsibius dujardini and Ramazzottius varieornatus
Source: PLoS Biol. 2017 Jul 27;15(7):e2002266. doi: 10.1371/journal.pbio.2002266 (PMC5531438; doi:10.1371/journal.pbio.2002266)
Supplement: S7 Table — (DOCX) [file pbio.2002266.s013.docx]

S7 Table

A. Proteomes used for protein family clustering

| **Index*** | **Species** | **GeneBuild ID / Accession** | **Source** |
| --- | --- | --- | --- |
| 0 | *Anopheles gambiae* | 2014-08-VectorBase | EnsemblMetazoa |
| 1 | *Apis mellifera* | 2014-05-BeeBase | EnsemblMetazoa |
| 2 | *Acyrthosiphon pisum* | 2013-07-AphidBase | EnsemblMetazoa |
| 3 | *Ascaris suum* | PRJNA80881 | WormbaseParasite5 |
| 4 | *Brugia malayi* | PRJNA10729 | WormbaseParasite5 |
| 5 | *Bursaphelenchus xylophilus* | PRJEA64437 | WormbaseParasite5 |
| 6 | *Caenorhabditis elegans* | PRJNA13758 | WormbaseParasite5 |
| 7 | *Cimex lectularius* | v0.5.3 | I5K |
| 8 | *Capitella teleta* | 2012-12-JGI | EnsemblMetazoa |
| 38 | *Drosophila melanogaster* | r6_09 | FLYBASE |
| 9 | *Dendroctonus ponderosae* | 2013-04-EnsemblMetazoa | EnsemblMetazoa |
| 10 | *Daphnia pulex* | 2011-02-EnsemblMetazoa | EnsemblMetazoa |
| 11 | *Hypsibius dujardini* | nHd.3.0 | this study |
| 12 | *Ixodes scapularis* | 2014-08-VectorBase | EnsemblMetazoa |
| 13 | *Meloidogyne hapla* | PRJNA29083 |  |
| 14 | *Nasonia vitripennis* | 2010-12-NasoniaBase | EnsemblMetazoa |
| 15 | *Octopus bimaculoides* | 2016-03-OIST | EnsemblMetazoa |
| 16 | *Priapulus caudatus* | GCF_000485595 | NCBI |
| 17 | *Pediculus humanus* | 2014-04-VectorBase | EnsemblMetazoa |
| 18 | *Plectus murrayi* | nPm.2.0 | ngenomes.org |
| 19 | *Pristionchus pacificus* | PRJNA12644 | WormbaseParasite5 |
| 20 | *Plutella xylostella* | DBM_FJ_v1_1 | LEPBASE |
| 37 | *Ramazzottius varieornatus* | nRv.1.1 | this study |
| 22 | *Solenopsis invicta* | 2013-10-AntGenomesPortal | EnsemblMetazoa |
| 23 | *Strigamia maritima* | 2013-02-EG | EnsemblMetazoa |
| 24 | *Tribolium castaneum* | 2012-09-24 | EnsemblMetazoa |
| 25 | *Trichuris muris* | PRJEB126 | WormbaseParasite5 |
| 26 | *Trichinella spiralis* | PRJNA12603 | WormbaseParasite5 |
| 27 | *Tetranychus urticae* | 2012-11-ORCAE | EnsemblMetazoa |

* The index assigned for OrthoFinder clustering

B. Transcriptome data used for clustering and phylogenomics.

| Species | Phylum | Data Type | Action | Link |
| --- | --- | --- | --- | --- |
| *Echiniscus testudo* | Tardigrada | assembled transcripts | predicted proteins using TransDecoder | ftp.ncbi.nlm.nih.gov/sra/wgs_aux/GD/AL/GDAL01/GDAL01.1.fsa_nt.gz |
| *Milnesium tardigradum* | Tardigrada | assembled transcripts | predicted proteins using TransDecoder | https://www.ncbi.nlm.nih.gov/nuccore/?term=PRJNA34121 |
| *Euperipatoides kanangrensis* | Onycophora | ESTs | assembled with CAP3; predicted proteins using TransDecoder | ftp://ftp-private.ncbi.nlm.nih.gov/pub/TraceDB/euperipatoides_kanangrensis/fasta.euperipatoides_kanangrensis.001.gz |
| *Peripatopsis sedgwicki* | Onycophora | ESTs | assembled with CAP3; predicted proteins using TransDecoder | https://www.ncbi.nlm.nih.gov/nucest/?term=Peripatopsis+sedgwicki%5Borganism%5D |
| *Peripatopsis capensis* | Onycophora | raw Illumina reads | trimmed using Skewer; assembled with Trinity; predicted proteins using TransDecoder | https://www.ncbi.nlm.nih.gov/bioproject/PRJNA236598 |
| *Pycnophyes kielensis* | Kinorhyncha | assembled transcripts | predicted proteins using TransDecoder | ftp://ftp.ncbi.nlm.nih.gov/sra/wgs_aux/GD/AN/GDAN01/GDAN01.1.fsa_nt.gz |
| *Echinoderes horni* | Kinorhyncha | ESTs | assembled with CAP3; predicted proteins using TransDecoder | ftp://ftp-private.ncbi.nlm.nih.gov/pub/TraceDB/echinoderes_horni/fasta.echinoderes_horni.001.gz |
| *Halicryptus spinulosus* | Priapulida | assembled transcripts | predicted proteins using TransDecoder | ftp://ftp.ncbi.nlm.nih.gov/sra/wgs_aux/GD/AM/GDAM01/GDAM01.1.fsa_nt.gz |
