# Supplementary material for: Comparative genomics of the tardigrades Hypsibius dujardini and Ramazzottius varieornatus
Source: PLoS Biol. 2017 Jul 27;15(7):e2002266. doi: 10.1371/journal.pbio.2002266 (PMC5531438; doi:10.1371/journal.pbio.2002266)
Supplement: S8 Table — (DOCX) [file pbio.2002266.s014.docx]

S8 Table. HGT content calculation of Ecdysozoa

| Category | Augustus3  model | Organism | ENSEMBL (DL:2016.09.20) | | | *Ab initio* (Augustus 3.2.2) | | |
| --- | --- | --- | --- | --- | --- | --- | --- | --- |
|  |  |  | # Gene | HGT | % | # Gene | HGT | % |
| Arthropod | aedes | *Aedes aegypti* | 15,796 | 182/11906 | 1.53 | 103,215 | 6364/70452 | 9.03 |
| Arthropod | honeybee1 | *Apis mellifera* | 15,314 | 485/9773 | 4.96 | 14,115 | 897/10073 | 8.90 |
| Arthropod | bombus_impatiens1 | *Bombus impatiens* | 15,896 | 1166/11329 | 10.29 | 18,793 | 1487/10910 | 13.63 |
| Arthropod | culex | *Culex quinquefasciatus* | 18,968 | 203/14015 | 1.45 | 25,343 | 297/18296 | 2.17 |
| Arthropod | NA | *Daphnia magna* * | 29,127 | 1343/17234 | 7.79 | NA | NA | NA |
| Arthropod | fly | *Drosophila ananassae* | 15,069 | 91/10336 | 0.88 | 21,842 | 1816/15694 | 11.58 |
| Arthropod | fly | *Drosopohila erecta* | 15,044 | 85/10121 | 0.84 | 15,447 | 616/11491 | 5.36 |
| Arthropod | fly | *Drosophila grimshawi* | 14,982 | 90/10448 | 0.86 | 15,293 | 263/11015 | 2.39 |
| Arthropod | fly | *Drosophila melanogaster* | 13,918 | 74/19191 | 0.73 | 15,535 | 532/11401 | 4.67 |
| Arthropod | fly | *Drosophila mojavensis* | 14,594 | 86/9918 | 0.87 | 15,677 | 372/11338 | 3.28 |
| Arthropod | fly | *Drosophila persimilis* | 16,874 | 82/10800 | 0.76 | 21,673 | 786/14385 | 5.46 |
| Arthropod | fly | *Drosophila pseudoobscura* | 15,864 | 96/10649 | 0.90 | 16,705 | 2452/11387 | 2.21 |
| Arthropod | fly | *Drosophila sechellia* | 16,465 | 71/10787 | 0.66 | 22,119 | 1151/16788 | 6.86 |
| Arthropod | fly | *Drosophila simulans* | 15,413 | 70/9820 | 0.71 | 16,148 | 362/11393 | 3.18 |
| Arthropod | fly | *Drosophila virilis* | 14,491 | 82/10081 | 0.81 | 15,991 | 512/11862 | 4.32 |
| Arthropod | fly | *Drosophila willistoni* | 15,512 | 156/10638 | 1.47 | 16,942 | 738/12259 | 6.02 |
| Arthropod | fly | *Drosophila yakuba* | 16,077 | 85/10463 | 0.81 | 17,774 | 539/12469 | 4.32 |
| Arthropod | heliconius_melpomene1 | *Heliconius_melpomene* | 12,669 | 132/9421 | 1.40 | 20,333 | 289/14640 | 1.97 |
| Arthropod | nasonia | *Nasonia vitripennis* | 17,083 | 223/12130 | 1.92 | 26,010 | 538/15457 | 3.48 |
| Arthropod | rhodnius | *Rhodnius prolixus* | 15,438 | 498/10733 | 4.63 | 52,161 | 1833/39130 | 4.68 |
| Arthropod | tribolium2012 | *Tribolium castaneum* | 16,524 | 124/1115 | 1.12 | 16,160 | 105/10442 | 1.00 |
| Nematode | caenorhabditis | *Caenorhabditis brenneri* | 30,660 | 253/14748 | 1.70 | 38,953 | 518/17298 | 2.99 |
| Nematode | caenorhabditis | *Caenorhabditis briggsae* | 21,814 | 239/10936 | 2.19 | 20,745 | 242/11335 | 2.13 |
| Nematode | caenorhabditis | *Caenorhabditis elegans* | 20,362 | 223/10574 | 2.11 | 18,177 | 215/10278 | 2.09 |
| Nematode | caenorhabditis | *Caenorhabditis japonica* | 29,931 | 315/15260 | 2.06 | 29,556 | 352/15842 | 2.22 |
| Nematode | caenorhabditis | *Caenorhabditis remanei* | 31,437 | 766/14483 | 5.29 | 30,506 | 1288/14291 | 9.01 |
| Nematode | trichinella | *Trichinella spiralis* | 16,380 | 47/8616 | 0.55 | 11,310 | 41/8079 | 0.51 |
| Tardigrade | BRAKER | *Hypsibius dujardini* | NA | NA | NA | 19913 | 463/12616 | 3.67 |
| Tardigrade | BRAKER | *Ramazzottius varieornatus* * | 19521 | 242/10760 | 2.25 | 13917 | 220/9894 | 2.22 |

* Genomes of these species were not registered in ENSEMBL, we used the released CDS sequences from each genome project
