# Supplementary material for: Comparative genomics of the tardigrades Hypsibius dujardini and Ramazzottius varieornatus
Source: PLoS Biol. 2017 Jul 27;15(7):e2002266. doi: 10.1371/journal.pbio.2002266 (PMC5531438; doi:10.1371/journal.pbio.2002266)
Supplement: S9 Table — (DOCX) [file pbio.2002266.s015.docx]

S9 Table. Tardigrade-specific, protection-related proteins

| Gene ID | Scaffold | Category | %Identity | Length | E-value | Bitscore |
| --- | --- | --- | --- | --- | --- | --- |
| bHd17608.1 | scaffold0022 | CAHS1 | 54.55 | 165 | 5.0E-45 | 158 |
| bHd17663.1 | scaffold0023 | CAHS1 | 49.46 | 184 | 2.0E-45 | 161 |
| bHd04182.1 | scaffold0087 | CAHS1 | 61.99 | 221 | 7.0E-65 | 209 |
| bHd04184.1 | scaffold0087 | CAHS1 | 78.18 | 55 | 4.0E-19 | 85.5 |
| bHd06166.1 | scaffold0123 | CAHS1 | 45.13 | 195 | 2.0E-51 | 174 |
| bHd16038.1 | scaffold0013 | CAHS2 | 56.55 | 168 | 8.0E-57 | 187 |
| bHd17504.1 | scaffold0022 | CAHS2 | 53.33 | 105 | 3.0E-28 | 117 |
| bHd17505.1 | scaffold0022 | CAHS2 | 47.40 | 192 | 3.0E-47 | 162 |
| bHd17506.1 | scaffold0022 | CAHS2 | 67.71 | 192 | 1.0E-82 | 253 |
| bHd18862.1 | scaffold0032 | CAHS2 | 51.63 | 184 | 9.0E-41 | 148 |
| bHd01486.1 | scaffold0050 | CAHS2 | 55.09 | 167 | 8.0E-54 | 179 |
| bHd02925.1 | scaffold0069 | CAHS3 | 57.33 | 75 | 2.0E-20 | 90.5 |
| bHd19902.1 | scaffold0018 | MAHS | 42.51 | 167 | 5.0E-24 | 107 |
| bHd16514.1 | scaffold0016 | RvLEAM | 41.45 | 234 | 3.0E-40 | 147 |
| bHd00493.1 | scaffold0002 | SAHS1 | 36.30 | 135 | 2.0E-24 | 100 |
| bHd07979.1 | scaffold0005 | SAHS1 | 34.50 | 171 | 2.0E-25 | 103 |
| bHd10755.1 | scaffold0239 | SAHS1 | 45.88 | 170 | 2.0E-44 | 152 |
| bHd10756.1 | scaffold0239 | SAHS1 | 38.01 | 171 | 2.0E-32 | 121 |
| bHd10757.1 | scaffold0239 | SAHS1 | 38.01 | 171 | 2.0E-32 | 121 |
| bHd10758.1 | scaffold0239 | SAHS1 | 54.23 | 142 | 3.0E-47 | 159 |
| bHd10759.1 | scaffold0239 | SAHS1 | 54.68 | 139 | 2.0E-49 | 164 |
| bHd10762.1 | scaffold0239 | SAHS1 | 47.37 | 171 | 5.0E-47 | 159 |
| bHd10763.1 | scaffold0239 | SAHS1 | 46.47 | 170 | 3.0E-47 | 159 |
| bHd10764.1 | scaffold0239 | SAHS1 | 69.63 | 135 | 2.0E-65 | 205 |
