# Supplementary material for: Comparative genomics of the tardigrades Hypsibius dujardini and Ramazzottius varieornatus
Source: PLoS Biol. 2017 Jul 27;15(7):e2002266. doi: 10.1371/journal.pbio.2002266 (PMC5531438; doi:10.1371/journal.pbio.2002266)
Supplement: S10 Table — (DOCX) [file pbio.2002266.s016.docx]

S10 Table. Synapomorphies identified under different systematic hypotheses

| **cluster_id** | **proteins** | **hypothesis*** | **taxon coverage** | **proportion of nematode proteomes present (n=9)** | **proportion of arthropode proteomes present (n=15)** | **proportion of tardigrade proteomes present (n=2)** |
| --- | --- | --- | --- | --- | --- | --- |
| OG0000436 | 104 | Panarthropoda | 1.00 | 0.00 | 1.00 | 1.00 |
| OG0001236 | 54 | Panarthropoda | 1.00 | 0.00 | 1.00 | 1.00 |
| OG0002592 | 36 | Panarthropoda | 1.00 | 0.00 | 1.00 | 1.00 |
| OG0006538 | 19 | Panarthropoda | 1.00 | 0.00 | 1.00 | 1.00 |
| OG0006541 | 19 | Panarthropoda | 1.00 | 0.00 | 1.00 | 1.00 |
| OG0006869 | 17 | Panarthropoda | 1.00 | 0.00 | 1.00 | 1.00 |
| OG0005117 | 27 | Panarthropoda | 0.88 | 0.00 | 0.93 | 0.50 |
| OG0005941 | 22 | Panarthropoda | 0.77 | 0.00 | 0.73 | 1.00 |
| OG0006662 | 18 | Panarthropoda | 0.82 | 0.00 | 0.80 | 1.00 |
| OG0006889 | 17 | Panarthropoda | 0.71 | 0.00 | 0.73 | 0.50 |
| OG0006940 | 17 | Panarthropoda | 0.82 | 0.00 | 0.87 | 0.50 |
| OG0006941 | 17 | Panarthropoda | 0.71 | 0.00 | 0.67 | 1.00 |
| OG0006951 | 17 | Panarthropoda | 0.71 | 0.00 | 0.67 | 1.00 |
| OG0007141 | 16 | Panarthropoda | 0.82 | 0.00 | 0.80 | 1.00 |
| OG0007285 | 15 | Panarthropoda | 0.71 | 0.00 | 0.67 | 1.00 |
| OG0007290 | 15 | Panarthropoda | 0.82 | 0.00 | 0.80 | 1.00 |
| OG0007298 | 15 | Panarthropoda | 0.88 | 0.00 | 0.87 | 1.00 |
| OG0007328 | 15 | Panarthropoda | 0.71 | 0.00 | 0.67 | 1.00 |
| OG0007463 | 14 | Panarthropoda | 0.77 | 0.00 | 0.73 | 1.00 |
| OG0007689 | 13 | Panarthropoda | 0.71 | 0.00 | 0.67 | 1.00 |
| OG0005423 | 26 | Nematoda+ Tardigrada | 0.82 | 0.89 | 0.00 | 0.50 |
| OG0006414 | 20 | Nematoda+ Tardigrada | 0.82 | 0.78 | 0.00 | 1.00 |
| OG0007199 | 16 | Nematoda+ Tardigrada | 0.91 | 1.00 | 0.00 | 0.50 |
| OG0007812 | 13 | Nematoda+ Tardigrada | 0.82 | 0.78 | 0.00 | 1.00 |
| OG0008368 | 11 | Nematoda+ Tardigrada | 0.82 | 0.78 | 0.00 | 1.00 |

* Panarthropoda = Tardigrada+Arthropoda
