# Supplementary material for: Comparative genomics of the tardigrades Hypsibius dujardini and Ramazzottius varieornatus
Source: PLoS Biol. 2017 Jul 27;15(7):e2002266. doi: 10.1371/journal.pbio.2002266 (PMC5531438; doi:10.1371/journal.pbio.2002266)
Supplement: S11 Table — (DOCX) [file pbio.2002266.s017.docx]

**S11 Table. Software used in this study.**

| Tool name | Ref. | Version | Relevant parameters * | Commentary on usage or web source |
| --- | --- | --- | --- | --- |
| Raw data processing and filtering | | | | |
| FastQC | [1] | v0.11.3 |  |  |
| Skewer | [2] | 0.2.2 | -n -q 30 -l 51 -m pe |  |
| bwa | [3] | 0.7.12-r1039 |  |  |
| blobtools | [4] | v0.9.19 |  | https://github.com/DRL/blobtools |
| samtools | [5] | Version: 1.2 (using htslib 1.2.1) | [view] -@ 30 –bS  [sort] -@ 30  [index] None |  |
| ncbi-blast+ | [6] | ncbi-blast-2.4.0+ |  |  |
| Genome assembly | | | | |
| Usearch | [7] | v. 8.0.1517 |  |  |
| SPAdes | [8] | v. 3.8.1 | -k 21,33,55,77,99,127, --only-assembler , --careful |  |
| Bowtie2 | [9] | v. 2.2.4 | None |  |
| Platanus | [10] | v. 1.2.3 | -u 0.2 |  |
| Falcon | [11] | v.0.2.2 | [Error correction]  daligner cutoff of 4,000 bp and -k16 -e0.70 -s1000 -t16 -l1000 -h64 -w7  [Second daligner]  -k20 -e.96 -s1000 -t32 -l1500 -h256  [Final assembly]  min coverage of 2, max coverage of 80, max diff coverage of 40 |  |
| SSPACE-LongReads | [12] | v. 1.1 | -m 50 |  |
| PBJelly | [13] | v. 13.10 | -m 50 |  |
| Pilon | [14] | v. 1.17 |  |  |
| Qualimap | [15] | v. 2.2 | qualimap bamqc -bam input.mem.sorted.bam -outformat pdf --java-mem-size=16G |  |
| CEGMA | [16] | v. 2.5 | None |  |
| BUSCO | [17] | v. 2.0.1 | Lineage : eukaryota_odb9, genome mode -sp fly |  |
| Phylogenetic analyses | | | | |
| RAxML | [18] | 8.2.8 | -b 12345 -# 100 -T 62 -p 12345 -m PROTGAMMAGTR |  |
| ClustalW2 | [19] | v2.1 |  |  |
| PhyloBayes | [20] | pb_mpi1.7a |  |  |
| MAFFT | [21] | v7.271 (2016/1/6) |  |  |
| FastTree | [22] | 2.1.8 SSE3 | -boot 1000 |  |
| trimal | [23] | v1.4.rev15 build[2013-12-17] |  |  |
| fasconcat-G | [24] | FASconCAT_v1.0.pl |  |  |
| FigTree | [25] | v. 1.4.2 |  |  |
| Annotation and databasing | | | | |
| ENSEMBL | [26] | Version 85 |  |  |
| EasyMirror and EasyImport | [27] | Version 0.9 |  |  |
| Hmmsearch | [28] | HMMER 3.1b2 (February 2015); | --cpu 32 --domE 1e-15 |  |
| Braker | [29] | v1.9 |  |  |
| Augustus | [30] | v3.2.2 |  |  |
| GeneMark-ES | [31] | v.4.21 |  |  |
| RepeatScout | [32] | Version 1.0.5 |  |  |
| RepeatMasker | [33] | version open-4.0.5 |  |  |
| tRNAscan-SE | [34] | tRNAscan-SE 1.3.1 (January 2012) |  |  |
| RNAmmer | [35] | v1.2 | -multi -S euk -m lsu,ssu,tsu |  |
| KAAS | [36] | Automatic Annotation Server Ver. 2.1 | Representative set for GENES | Via web interface. |
| Legacy blast | [37] | v2.2.22 | -e 1e-15 |  |
| Diamond | [38] | v1.8.2 | -e 10 --sensitive |  |
| InterProScan | [19] | interproscan-5.19-58.0 | --goterms -appl TIGRFAM-15.0,ProDom-2006.1,SMART-7.1,SignalP-EUK-4.1,PrositePatterns-20.119,PRINTS-42.0,SuperFamily-1.75,Pfam-29.0,PrositeProfiles-20.119 -f TSV | https://github.com/ebi-pf-team/interproscan |
| miRDeep | [39] | v.2.0.0.8 |  |  |
| SSEARCH | [19] | v36.3.8e Sep, 2016(preload9) |  |  |
| Genome comparison | | | | |
| murasaki | [40] | Murasaki version 1.68.6 (LARGESEQ) | -p[28:36] -M 100 |  |
| Mauve | [41] | mauve_snapshot_2015-02-13/ |  | Progressive alignment with GUI |
| Databases used in annotation** | | | | |
| Swiss-Prot | [42] | 2016/5/23 |  |  |
| TrEMBL | [42] | 2016/1/17 |  |  |
| Pfam-A | [43] | 2016/7/22 |  |  |
| Dfam | [44] | 2016/09/26 |  |  |
| miRBase | [45] | 2016/12/20 |  |  |
| Transcriptome analyses | | | | |
| Trinity | [46] | 2.2.0 | Default params |  |
| TransDecoder | [47] | 3.0.0 | TransDecoder.LongOrfs (default)  TransDecoder.Predict --retain_blastp_hits --single_best_orf |  |
| Tophat2 | [48] | v2.1.1 | tophat -o output -p 30 ref.fa a.r1.fq a.r2.fq |  |
| DESeq2 | [49] | 1.8.2 |  |  |
| Bowtie2 | [9] | version 2.2.8 |  | Used within TopHat2 |
| Kallisto | [50] | kallisto 0.42.4 | [index] None  [quant-single] --bias -b 100 --single -l 200 -s 50  [quant-paired] --bias --b 100 |  |
| Gene family analyses | | | | |
| Gephi | [51] | v0,9.1 | “Scaling” = 10000.0, “Stronger Gravity” = True, “Gravity” = 1.0, “Dissuade hubs” = False, “LinLog mode” = True, “Prevent overlap” = False, “Edge Weight Influence” = 1.0 | https://gephi.org/ |
| KinFin | [52] | v0.8.2 |  | https://github.com/DRL/kinfin |
| OrthoFinder | [53] | v1.1.2 | Using the following inflation values: 1.1, 1.5, 2.0, 2.5, 3.5, 4.0, 4.5, 5.0 | https://github.com/davidemms/OrthoFinder |
|  |  |  |  |  |
| Others | | | | |
|  |  |  |  |  |
| G-language Genome Analysis Environment | [54, 55] | v.1.9.1 |  |  |

* Where no entry is made, the program was used with default settings.

** For databases, the date of download is given as version.

**Reference**

1. Andrews S. FastQC a quality-control tool for high-throughput sequence data. 2015 [cited 2015 May 21]. Available from: <http://www.bioinformatics.babraham.ac.uk/projects/fastqc/>.

2. Jiang H, Lei R, Ding SW, Zhu S. Skewer: a fast and accurate adapter trimmer for next-generation sequencing paired-end reads. BMC Bioinformatics. 2014;15:182. doi: 10.1186/1471-2105-15-182. PubMed PMID: 24925680; PubMed Central PMCID: PMCPMC4074385.

3. Li H, Durbin R. Fast and accurate short read alignment with Burrows-Wheeler transform. Bioinformatics. 2009;25(14):1754-60. doi: 10.1093/bioinformatics/btp324. PubMed PMID: 19451168; PubMed Central PMCID: PMCPMC2705234.

4. Kumar S, Jones M, Koutsovoulos G, Clarke M, Blaxter M. Blobology: exploring raw genome data for contaminants, symbionts and parasites using taxon-annotated GC-coverage plots. Front Genet. 2013;4:237. doi: 10.3389/fgene.2013.00237. PubMed PMID: 24348509; PubMed Central PMCID: PMCPMC3843372.

5. Li H, Handsaker B, Wysoker A, Fennell T, Ruan J, Homer N, et al. The Sequence Alignment/Map format and SAMtools. Bioinformatics. 2009;25(16):2078-9. doi: 10.1093/bioinformatics/btp352. PubMed PMID: 19505943; PubMed Central PMCID: PMCPMC2723002.

6. Camacho C, Coulouris G, Avagyan V, Ma N, Papadopoulos J, Bealer K, et al. BLAST+: architecture and applications. BMC Bioinformatics. 2009;10:421. doi: 10.1186/1471-2105-10-421. PubMed PMID: 20003500; PubMed Central PMCID: PMCPMC2803857.

7. Edgar RC. Search and clustering orders of magnitude faster than BLAST. Bioinformatics. 2010;26(19):2460-1. doi: 10.1093/bioinformatics/btq461. PubMed PMID: 20709691.

8. Bankevich A, Nurk S, Antipov D, Gurevich AA, Dvorkin M, Kulikov AS, et al. SPAdes: a new genome assembly algorithm and its applications to single-cell sequencing. J Comput Biol. 2012;19(5):455-77. doi: 10.1089/cmb.2012.0021. PubMed PMID: 22506599; PubMed Central PMCID: PMCPMC3342519.

9. Langmead B, Salzberg SL. Fast gapped-read alignment with Bowtie 2. Nat Methods. 2012;9(4):357-9. doi: 10.1038/nmeth.1923. PubMed PMID: 22388286; PubMed Central PMCID: PMCPMC3322381.

10. Kajitani R, Toshimoto K, Noguchi H, Toyoda A, Ogura Y, Okuno M, et al. Efficient de novo assembly of highly heterozygous genomes from whole-genome shotgun short reads. Genome Res. 2014;24(8):1384-95. doi: 10.1101/gr.170720.113. PubMed PMID: 24755901; PubMed Central PMCID: PMCPMC4120091.

11. Chin CS, Peluso P, Sedlazeck FJ, Nattestad M, Concepcion GT, Clum A, et al. Phased diploid genome assembly with single-molecule real-time sequencing. Nat Methods. 2016;13(12):1050-4. doi: 10.1038/nmeth.4035. PubMed PMID: 27749838.

12. Boetzer M, Pirovano W. SSPACE-LongRead: scaffolding bacterial draft genomes using long read sequence information. BMC Bioinformatics. 2014;15(211):211. doi: 10.1186/1471-2105-15-211. PubMed PMID: 24950923; PubMed Central PMCID: PMCPMC4076250.

13. English AC, Richards S, Han Y, Wang M, Vee V, Qu J, et al. Mind the gap: upgrading genomes with Pacific Biosciences RS long-read sequencing technology. PLoS One. 2012;7(11):e47768. doi: 10.1371/journal.pone.0047768. PubMed PMID: 23185243; PubMed Central PMCID: PMCPMC3504050.

14. Walker BJ, Abeel T, Shea T, Priest M, Abouelliel A, Sakthikumar S, et al. Pilon: an integrated tool for comprehensive microbial variant detection and genome assembly improvement. PLoS One. 2014;9(11):e112963. doi: 10.1371/journal.pone.0112963. PubMed PMID: 25409509; PubMed Central PMCID: PMCPMC4237348.

15. Okonechnikov K, Conesa A, Garcia-Alcalde F. Qualimap 2: advanced multi-sample quality control for high-throughput sequencing data. Bioinformatics. 2016;32(2):292-4. doi: 10.1093/bioinformatics/btv566. PubMed PMID: 26428292; PubMed Central PMCID: PMCPMC4708105.

16. Parra G, Bradnam K, Korf I. CEGMA: a pipeline to accurately annotate core genes in eukaryotic genomes. Bioinformatics. 2007;23(9):1061-7. doi: 10.1093/bioinformatics/btm071. PubMed PMID: 17332020.

17. Simao FA, Waterhouse RM, Ioannidis P, Kriventseva EV, Zdobnov EM. BUSCO: assessing genome assembly and annotation completeness with single-copy orthologs. Bioinformatics. 2015;31(19):3210-2. doi: 10.1093/bioinformatics/btv351. PubMed PMID: 26059717.

18. Stamatakis A. RAxML version 8: a tool for phylogenetic analysis and post-analysis of large phylogenies. Bioinformatics. 2014;30(9):1312-3. doi: 10.1093/bioinformatics/btu033. PubMed PMID: 24451623; PubMed Central PMCID: PMCPMC3998144.

19. Goujon M, McWilliam H, Li W, Valentin F, Squizzato S, Paern J, et al. A new bioinformatics analysis tools framework at EMBL-EBI. Nucleic Acids Res. 2010;38(Web Server issue):W695-9. doi: 10.1093/nar/gkq313. PubMed PMID: 20439314; PubMed Central PMCID: PMCPMC2896090.

20. Lartillot N, Philippe H. A Bayesian mixture model for across-site heterogeneities in the amino-acid replacement process. Mol Biol Evol. 2004;21(6):1095-109. doi: 10.1093/molbev/msh112. PubMed PMID: 15014145.

21. Katoh K, Standley DM. MAFFT multiple sequence alignment software version 7: improvements in performance and usability. Mol Biol Evol. 2013;30(4):772-80. doi: 10.1093/molbev/mst010. PubMed PMID: 23329690; PubMed Central PMCID: PMCPMC3603318.

22. Price MN, Dehal PS, Arkin AP. FastTree 2--approximately maximum-likelihood trees for large alignments. PLoS One. 2010;5(3):e9490. doi: 10.1371/journal.pone.0009490. PubMed PMID: 20224823; PubMed Central PMCID: PMCPMC2835736.

23. Capella-Gutierrez S, Silla-Martinez JM, Gabaldon T. trimAl: a tool for automated alignment trimming in large-scale phylogenetic analyses. Bioinformatics. 2009;25(15):1972-3. doi: 10.1093/bioinformatics/btp348. PubMed PMID: 19505945; PubMed Central PMCID: PMCPMC2712344.

24. Kuck P, Longo GC. FASconCAT-G: extensive functions for multiple sequence alignment preparations concerning phylogenetic studies. Front Zool. 2014;11(1):81. doi: 10.1186/s12983-014-0081-x. PubMed PMID: 25426157; PubMed Central PMCID: PMCPMC4243772.

25. Rambaut A. FigTree. 2016.

26. Aken BL, Achuthan P, Akanni W, Amode MR, Bernsdorff F, Bhai J, et al. Ensembl 2017. Nucleic Acids Res. 2017;45(D1):D635-D42. doi: 10.1093/nar/gkw1104. PubMed PMID: 27899575; PubMed Central PMCID: PMCPMC5210575.

27. Challis RJ, Kumar S, Stevens L, Blaxter M. EasyMirror and EasyImport: Simplifying the setup of a custom Ensembl database and webserver for any species. PeerJ Preprints. 2016;4(e2401v1 ). doi: 10.7287/peerj.preprints.2401v1.

28. Mistry J, Finn RD, Eddy SR, Bateman A, Punta M. Challenges in homology search: HMMER3 and convergent evolution of coiled-coil regions. Nucleic Acids Res. 2013;41(12):e121. doi: 10.1093/nar/gkt263. PubMed PMID: 23598997; PubMed Central PMCID: PMCPMC3695513.

29. Hoff KJ, Lange S, Lomsadze A, Borodovsky M, Stanke M. BRAKER1: Unsupervised RNA-Seq-Based Genome Annotation with GeneMark-ET and AUGUSTUS. Bioinformatics. 2016;32(5):767-9. doi: 10.1093/bioinformatics/btv661. PubMed PMID: 26559507.

30. Keller O, Kollmar M, Stanke M, Waack S. A novel hybrid gene prediction method employing protein multiple sequence alignments. Bioinformatics. 2011;27(6):757-63. doi: 10.1093/bioinformatics/btr010. PubMed PMID: 21216780.

31. Borodovsky M, Lomsadze A. Eukaryotic gene prediction using GeneMark.hmm-E and GeneMark-ES. Curr Protoc Bioinformatics. 2011;Chapter 4:Unit 4 6 1-10. doi: 10.1002/0471250953.bi0406s35. PubMed PMID: 21901742; PubMed Central PMCID: PMCPMC3204378.

32. Price AL, Jones NC, Pevzner PA. De novo identification of repeat families in large genomes. Bioinformatics. 2005;21 Suppl 1:i351-8. doi: 10.1093/bioinformatics/bti1018. PubMed PMID: 15961478.

33. Smit A, Hubley R, Green P. RepeatMasker Open-4.0 2013-2015. Available from: <http://www.repeatmasker.org>.

34. Lowe TM, Eddy SR. tRNAscan-SE: a program for improved detection of transfer RNA genes in genomic sequence. Nucleic Acids Res. 1997;25(5):955-64. doi: 10.1093/nar/25.5.0955. PubMed PMID: 9023104; PubMed Central PMCID: PMCPMC146525.

35. Lagesen K, Hallin P, Rodland EA, Staerfeldt HH, Rognes T, Ussery DW. RNAmmer: consistent and rapid annotation of ribosomal RNA genes. Nucleic Acids Res. 2007;35(9):3100-8. doi: 10.1093/nar/gkm160. PubMed PMID: 17452365; PubMed Central PMCID: PMCPMC1888812.

36. Moriya Y, Itoh M, Okuda S, Yoshizawa AC, Kanehisa M. KAAS: an automatic genome annotation and pathway reconstruction server. Nucleic Acids Res. 2007;35(Web Server issue):W182-5. doi: 10.1093/nar/gkm321. PubMed PMID: 17526522; PubMed Central PMCID: PMCPMC1933193.

37. Altschul SF, Madden TL, Schaffer AA, Zhang J, Zhang Z, Miller W, et al. Gapped BLAST and PSI-BLAST: a new generation of protein database search programs. Nucleic Acids Res. 1997;25(17):3389-402. PubMed PMID: 9254694; PubMed Central PMCID: PMCPMC146917.

38. Buchfink B, Xie C, Huson DH. Fast and sensitive protein alignment using DIAMOND. Nature Methods. 2015;12(1):59-60. PubMed PMID: WOS:000347668600019.

39. Friedlander MR, Mackowiak SD, Li N, Chen W, Rajewsky N. miRDeep2 accurately identifies known and hundreds of novel microRNA genes in seven animal clades. Nucleic Acids Res. 2012;40(1):37-52. doi: 10.1093/nar/gkr688. PubMed PMID: 21911355; PubMed Central PMCID: PMCPMC3245920.

40. Popendorf K, Tsuyoshi H, Osana Y, Sakakibara Y. Murasaki: a fast, parallelizable algorithm to find anchors from multiple genomes. PLoS One. 2010;5(9):e12651. doi: 10.1371/journal.pone.0012651. PubMed PMID: 20885980; PubMed Central PMCID: PMCPMC2945767.

41. Darling AC, Mau B, Blattner FR, Perna NT. Mauve: multiple alignment of conserved genomic sequence with rearrangements. Genome Res. 2004;14(7):1394-403. doi: 10.1101/gr.2289704. PubMed PMID: 15231754; PubMed Central PMCID: PMCPMC442156.

42. UniProt C. UniProt: a hub for protein information. Nucleic Acids Res. 2015;43(Database issue):D204-12. doi: 10.1093/nar/gku989. PubMed PMID: 25348405; PubMed Central PMCID: PMCPMC4384041.

43. Finn RD, Bateman A, Clements J, Coggill P, Eberhardt RY, Eddy SR, et al. Pfam: the protein families database. Nucleic Acids Res. 2014;42(Database issue):D222-30. doi: 10.1093/nar/gkt1223. PubMed PMID: 24288371; PubMed Central PMCID: PMCPMC3965110.

44. Hubley R, Finn RD, Clements J, Eddy SR, Jones TA, Bao W, et al. The Dfam database of repetitive DNA families. Nucleic Acids Res. 2016;44(D1):D81-9. doi: 10.1093/nar/gkv1272. PubMed PMID: 26612867; PubMed Central PMCID: PMCPMC4702899.

45. Kozomara A, Griffiths-Jones S. miRBase: annotating high confidence microRNAs using deep sequencing data. Nucleic Acids Res. 2014;42(Database issue):D68-73. doi: 10.1093/nar/gkt1181. PubMed PMID: 24275495; PubMed Central PMCID: PMCPMC3965103.

46. Grabherr MG, Haas BJ, Yassour M, Levin JZ, Thompson DA, Amit I, et al. Full-length transcriptome assembly from RNA-Seq data without a reference genome. Nat Biotechnol. 2011;29(7):644-52. doi: 10.1038/nbt.1883. PubMed PMID: 21572440; PubMed Central PMCID: PMCPMC3571712.

47. Haas BJ, Papanicolaou A. TransDecoder (Find Coding Regions Within Transcripts).

48. Kim D, Pertea G, Trapnell C, Pimentel H, Kelley R, Salzberg SL. TopHat2: accurate alignment of transcriptomes in the presence of insertions, deletions and gene fusions. Genome Biol. 2013;14(4):R36. doi: 10.1186/gb-2013-14-4-r36. PubMed PMID: 23618408; PubMed Central PMCID: PMCPMC4053844.

49. Love MI, Huber W, Anders S. Moderated estimation of fold change and dispersion for RNA-seq data with DESeq2. Genome Biol. 2014;15(12):550. doi: 10.1186/s13059-014-0550-8. PubMed PMID: 25516281; PubMed Central PMCID: PMCPMC4302049.

50. Bray NL, Pimentel H, Melsted P, Pachter L. Near-optimal probabilistic RNA-seq quantification. Nat Biotechnol. 2016;34(5):525-7. doi: 10.1038/nbt.3519. PubMed PMID: 27043002.

51. Mathieu B, Sebastien H, Mathieu J. Gephi: An Open Source Software for Exploring and Manipulating Networks. 2009 %9 network;network science;visualization;graph exploration;open source;free software;dynamic network;interactive interface;graph;force vector;java;OpenGL;3-D visualization;user-centric;graph layout;complex graph rendering;network analysis;webatlas %! Gephi: An Open Source Software for Exploring and Manipulating Networks2009.

52. Laetsch DR. KinFin v0.8.1. 2017. doi: 10.5281/zenodo.290589.

53. Emms DM, Kelly S. OrthoFinder: solving fundamental biases in whole genome comparisons dramatically improves orthogroup inference accuracy. Genome Biol. 2015;16:157. doi: 10.1186/s13059-015-0721-2. PubMed PMID: 26243257; PubMed Central PMCID: PMCPMC4531804.

54. Arakawa K, Mori K, Ikeda K, Matsuzaki T, Kobayashi Y, Tomita M. G-language Genome Analysis Environment: a workbench for nucleotide sequence data mining. Bioinformatics. 2003;19(2):305-6. PubMed PMID: 12538262.

55. Arakawa K, Tomita M. G-language system as a platform for large-scale analysis of high-throughput omics data. J Pestic Sci. 2006;31(3):282-8. doi: DOI 10.1584/jpestics.31.282. PubMed PMID: WOS:000240114400006.
